# Assessing the Utility of Multiplexed Liquid Chromatography-Mass Spectrometry for Gluten Detection in Australian Breakfast Food Products

**DOI:** 10.3390/molecules24203665

**Published:** 2019-10-11

**Authors:** Haili Li, Utpal Bose, Sally Stockwell, Crispin A. Howitt, Michelle Colgrave

**Affiliations:** 1 CSIRO Agriculture and Food, 306 Carmody Rd., St. Lucia, QLD 4067, Australia; haili8693@sina.com (H.L.); utpal.bose@csiro.au (U.B.); sally.stockwell@csiro.au (S.S.); 2 Institute of Animal Husbandry and Veterinary Science, Henan Academy of Agricultural Sciences, Zhengzhou 450002, China; 3 CSIRO Agriculture and Food, GPO Box 1700, Canberra, ACT 2601, Australia; crispin.howitt@csiro.au; 4 School of Science, Edith Cowan University, 270 Joondalup Dr., Joondalup, WA 6027, Australia

**Keywords:** Coeliac disease (CD), gluten, peptide markers, liquid chromatography-mass spectrometry (LC-MS)

## Abstract

Coeliac disease (CD) is an autoimmune disorder triggered by the ingestion of gluten that is associated with gastrointestinal issues, including diarrhea, abdominal pain, and malabsorption. Gluten is a general name for a class of cereal storage proteins of wheat, barley, and rye that are notably resistant to gastrointestinal digestion. After ingestion, immunogenic peptides are subsequently recognized by T cells in the gastrointestinal tract. The only treatment for CD is a life-long gluten-free diet. As such, it is critical to detect gluten in diverse food types, including those where one would not expect to find gluten. The utility of liquid chromatography-mass spectrometry (LC-MS) using cereal-specific peptide markers to detect gluten in heavily processed food types was assessed. A range of breakfast products, including breakfast cereals, breakfast bars, milk-based breakfast drinks, powdered drinks, and a savory spread, were tested. No gluten was detected by LC-MS in the food products labeled gluten-free, yet enzyme-linked immunosorbent assay (ELISA) measurement revealed inconsistencies in barley-containing products. In products containing wheat, rye, barley, and oats as labeled ingredients, gluten proteins were readily detected using discovery proteomics. Panels comprising ten cereal-specific peptide markers were analyzed by targeted proteomics, providing evidence that LC-MS could detect and differentiate gluten in complex matrices, including baked goods and milk-based products.

## 1. Introduction

Coeliac disease (CD) is a chronic, immune-mediated intestinal disorder characterized by an inflammatory response to dietary gluten that can develop in genetically susceptible individuals [1]. Gluten is a general name for a class of seed storage proteins from wheat (gliadins and glutenins), barley (hordeins), rye (secalins), and oats (avenins) that can be sub-divided into the alcohol-soluble prolamins and insoluble glutelins. 

Gluten contains a higher proportion of proline and glutamine, hence the term prolamin, than the average seed storage protein, and they also contain fewer tryptic sites, rendering them recalcitrant to digestion in the gastrointestinal tract. Gluten evades complete digestion by host proteases and gut microbiota, resulting in the generation of gluten immunogenic peptides [2,3]. CD has a recorded prevalence of 1–3% in Western populations, with 0.7% of the US population diagnosed with CD [4]. More recently, the prevalence of CD in Australia was noted to be 1.2% for men and 1.9% for women [5]. Symptomatic manifestations of CD include diarrhea, weight loss, abdominal distention, abdominal pain, bloating, and malabsorption of nutrients, which can lead to iron deficiency and anemia and/or premature metabolic bone disease [6]. Intestinal features of CD include villous atrophy, crypt hyperplasia, intraepithelial lymphocytosis, and an influx of immune cells in the lamina propria [7]. The long-standing CD can result in complications, including refractory coeliac sprue, enteropathy-associated T cell lymphoma, and small intestinal adenocarcinoma [8,9].

The management of CD relies upon a strict, lifelong gluten-free diet (GFD) [10]. A threshold of 20 mg/kg (or 20 ppm) of gluten for foods to be labeled as “gluten-free” has been established by the U.S. Food and Drug Administration (FDA) and the Codex Alimentarius Commission. Specifically, the FDA defined the term “gluten-free” meaning that the food is inherently gluten-free; or does not contain an ingredient that is: (1) a gluten-containing grain (e.g., spelt wheat); (2) derived from a gluten-containing grain that has not been processed to remove gluten (e.g., wheat flour); or (3) derived from a gluten-containing grain that has been processed to remove gluten (e.g., wheat starch), if the use of that ingredient results in the presence of 20 mg/kg or more gluten in the food. Also, any unavoidable presence of gluten, i.e., resulting from cross-contact, in the food must be less than 20 mg/kg. In Australia, “gluten-free” claims refer to no detectable gluten, whereas “low gluten” refers to foods containing <200 mg/kg gluten. Moreover, in Australia, oats are not currently considered safe for people with CD and have been included in this study.

Enzyme-linked immunosorbent assays (ELISAs) and lateral flow devices exist as validated methods for gluten detection and quantitation in foods [11]. However, questions remain over the quantitative accuracy in processed foods, especially those affected by protein hydrolysis [12]. In a recent study, the performance of 14 ELISA kits for gluten detection was evaluated in a series of relevant food matrices varying in complexity [13]. Their results showed that there is no single ELISA method that can accurately detect and quantify gluten in all the different matrices. A separate study employing five sandwich ELISA kits to quantify gluten derived from wheat, rye, and barley showed large discrepancies dependent on the grain source, notably, worst when comparing wheat and barley glutelins. While wheat prolamins were detected quite accurately by all antibodies, high variability between antibody specificities and sensitivities was observed for rye and barley prolamins and rye glutelins. The gluten content was either overestimated up to six times (rye) or underestimated up to seven times (barley), with underestimation representing a serious health risk for people with CD [14]. 

**Figure 1:**
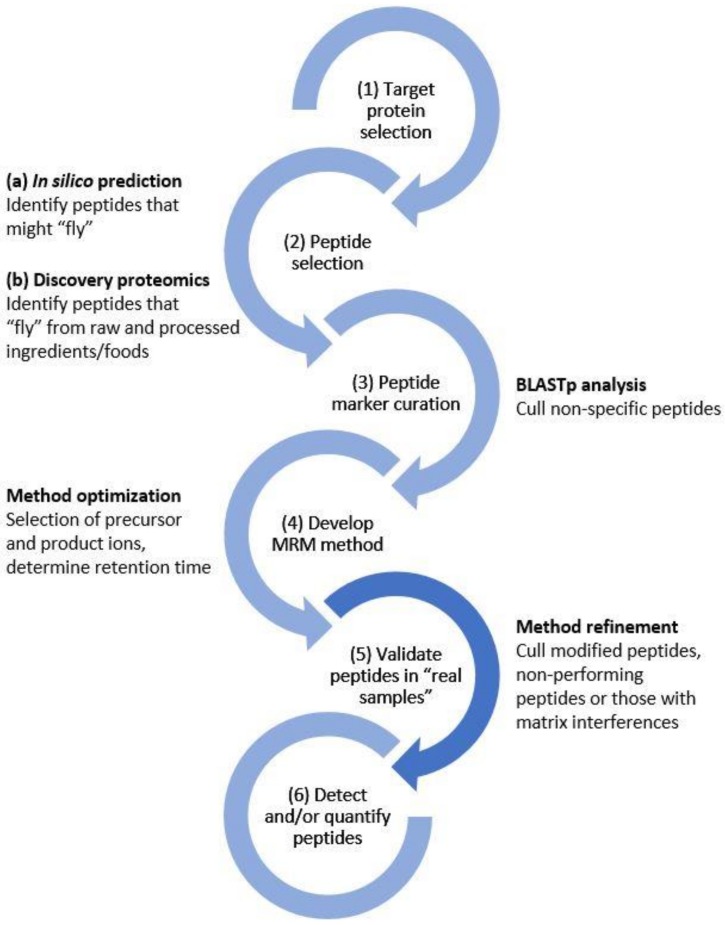
Generic workflow for the development of the LC-MRM-MS method. At each step, potential peptide markers were filtered according to whether they were experimentally detected (step 2), their specificity to the protein(s) of interest (step 3), their performance by LC-MS, the presence of modifications, or matrix effects (steps 4,5). In this study, the emphasis was placed on step (5) to ensure that peptide markers selected from raw ingredients would persist in a diverse set of heavily processed foods. MRM, multiple reaction monitoring.

Liquid chromatography-mass spectrometry (LC-MS) has been touted as a promising tool for disease biomarker elucidation and verification, owing to its sensitivity, precision, accuracy, and robust quantitative ability. Selected/multiple reaction monitoring (SRM/MRM) is a targeted proteomics approach that can be used for protein quantitation and validation of peptide markers for clinical applications [15], but also shows great promise in food testing applications [16]. LC-MRM-MS has been applied to gluten detection in food and beverages [17,18,19,20,21,22,23,24]. Figure 1 shows a generalized workflow for the development of an MRM method based on peptide markers specific to a target protein(s). The broad range of foods and the complexity of the matrices are a challenge that analytical methods must address. Moreover, the gluten proteins may be modified during processing by increased temperature and/or pressure. These modifications can result in changes to the primary structure affecting the peptide sequence (e.g., deamidation, oxidation, hydrolysis) or to the protein structure (e.g., the formation of cross-links). LC-MS offers potential in this area as it can simultaneously detect multiple peptides from a protein precursor enabling avoidance of modified sites. In this study, LC-MS was applied to detect gluten from wheat, rye, barley, and oats in a wide range of commercially available Australian breakfast food products. The objective was to determine whether peptide markers characterized in minimally processed grains (flours) would persist in a diverse set of heavily processed products.

## 2. Results and Discussion

Mass spectrometry (MS) is an accurate, reliable, and sensitive tool for peptide identification and quantitation and has great potential in the food sciences. It can be applied to the identification and characterization of bioactive peptides, but also, as is the case in this study, to detect potentially harmful proteins and their peptide fragments. As there is no treatment, people with CD or those with gluten intolerance need to select food products that are gluten-free to avoid intestinal damage and the raft of other related symptoms. As questions remain over the accuracy of ELISA measurement in highly processed food [12], this study has focused on the utility of LC-MS to detect grain-specific peptide markers in a range of Australian breakfast foods. Peptide markers that have previously [17,19,23,24] been determined to be specific to their source grain and show ideal analytical properties were investigated to ensure that they persist in heavily processed foods and were free from interference in complex matrices. By evaluating the utility of peptide markers in finished products, this approach represents savings in time and money, allowing peptide synthesis for absolute quantitation.

Using discovery proteomics, the gluten-enriched protein complement of the range of food products was first profiled. The spectral datasets arising from each type of food (BC, breakfast cereal; BB, breakfast bar; BM, milk-based breakfast drink; PD, powdered drink; or SS, savory spread) were compiled into one search per food type to generate a uniform set of protein accessions, and the results are summarized in Supplementary Tables S1–S5, respectively. A total of 720 (BC), 524 (BB), 336 (BM), 265 (PD), and 89 (SS) proteins were confidently identified when employing a 1% false discovery rate (FDR). In the dried products, 13–16% of the protein identifications were of gluten proteins: BCs, 94 gluten proteins (13% of total protein identifications); BBs, 75 gluten proteins (14%); and PDs, 42 gluten proteins (16%). In the milk-based products, only 2% (7 of the 336 proteins identified) were denoted as gluten proteins. In the savory spread, 7% (6 of the 89 proteins identified) were denoted as gluten proteins. Examining the food products individually, breakfast bar 1 (BB1) and milk-based breakfast drink 1 (BM1) were devoid of gluten protein identifications, affirming their status as gluten-free (GF) food products. In these two GF products, wherein rice was a labeled ingredient, rice glutelin proteins were the primary identifications, but other annotated allergenic proteins were identified (e.g., Uniprot: Q8H4M4 in BB1). Despite the nil detection of gluten by LC-MS, the result of the ELISA analysis (Table 1) showed a low level (14 mg/kg) for BB1. This level is below the “gluten-free” threshold of 20 mg/kg adhered to in the northern hemisphere, but this potential false positive would not adhere to the Australian requirement for nil detection. 

In the breakfast, cereals comprising a mixture of grains, proteins from all wheat gluten classes were detected. Additionally, barley hordeins, rye secalins, and oat avenins were identified, reflecting the ingredient lists (inclusive of barley, oats, and triticale). Likewise, in the breakfast bars, a diverse range of wheat gluten proteins, as well as gluten proteins from rye, barley, and oats, were detected. In BM2 and BM3, the gluten proteins identified included the B-hordeins (I6TRT5, Q3YAF9), D-hordein (I6TRS8), and γ-3-hordein (I6TEV2) from barley, and the avenins (P27919, I4EP67) from oats. In the powdered drinks made primarily from malted barley and wheat, the primary gluten identifications included the barley hordeins (B-, D-, and γ-), wheat α-/γ-gliadins, high molecular weight glutenins (HMW-GS),low molecular weight glutenins (LMW-GS), and wheat ALPs (avenin-like A proteins). 

**Figure 2:**
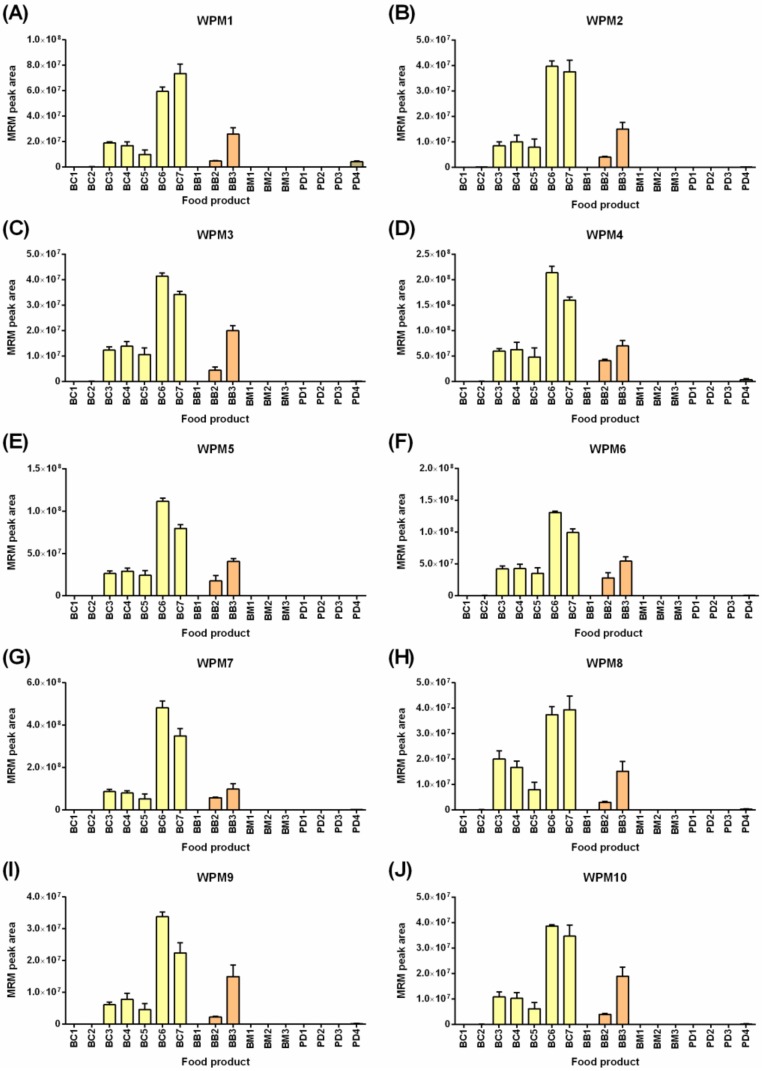
LC-MRM-MS analysis of 10 wheat peptide markers (WPMs) across a range of commercial Australian breakfast food products. The peak area + SD is plotted (*n* = 4). Panels (**A**) through (**J**) refer to the ten WPMs defined in Table S12.

Using the discovery data, the presence of the 37 high responding gluten peptides (10 from wheat, 7 from rye, 10 from barley, and 10 from oats) identified in previous studies were confirmed and subsequently used for LC-MRM-MS analysis. Figure 2 shows the results of the LC-MRM-MS analysis of 10 selected wheat peptides markers (WPMs). As expected, the WPMs were present in high abundance in breakfast cereals (BC3-BC7) and breakfast biscuits (BB2-BB3), which all bore wheat as a primary ingredient. Trace levels of wheat were also found in the barley-containing powdered drinks (PD1–3), wherein PD1 and PD2 did not state wheat as an ingredient, and PD3 named wheat starch. None of these powdered drinks bore a “gluten-free” claim as they contain barley as a primary ingredient. This study intended to confirm the applicability of the peptide markers across a broad range of products, and this was achieved. All wheat gluten peptides monitored were detected in this range of food products, and the pattern of abundance was noted to be highly similar. Two-way ANOVA revealed no significant differences based on the peptide marker (0.18% of the variation, *p* = 0.22), indicating that the ten selected peptide markers were not extensively modified or degraded during processing. Future experiments exploring incursion of starting materials and monitoring the peptides across the processing pipeline will constitute the ultimate evidence of the robustness of these peptide markers. All ten WPMs were consistently detected in BC3-BC7, BB2-BB3, and at low levels in PD4. The α-gliadin derived marker (WPM1) was detected at the highest level in PD4, whereas the γ-gliadin marker (WPM4) and the HMW-GS marker (WPM7) yielded the highest MS response and as such are deemed the most sensitive peptide markers for wheat. All 10 WPMs were free from interferences in the diverse matrices tested.

**Figure 3:**
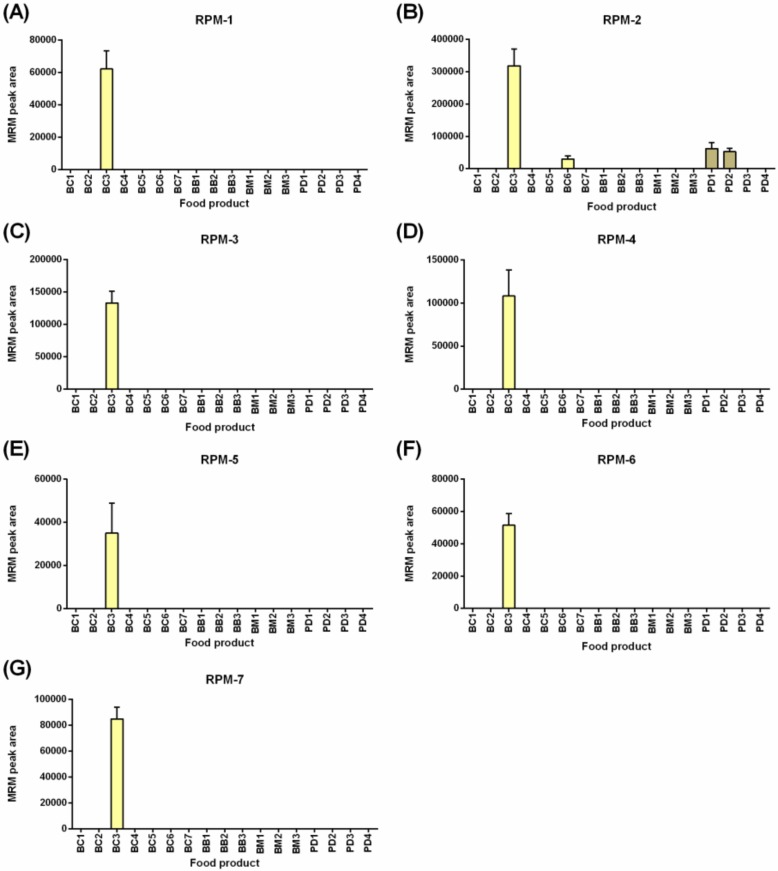
LC-MRM-MS analysis of seven rye peptide markers (RPMs) across a range of commercial Australian breakfast food products. The peak area + SD is plotted (*n* = 4). Panels (**A**) through (**G**) refer to the ten RPMs defined in Table S12.

Figure 3 shows the detection of seven rye gluten peptide markers (RPMs), noting that rye was not a labeled ingredient in any of the food products tested. Breakfast cereal BC3 contained triticale, a hybrid resulting from the crossing of wheat (Triticum) and rye (Secale). The levels of the RPMs were low with peak areas less than 3e^5^, 1000 times lower when compared to wheat detection at ~3e^8^ (Figure 2) in the same products, indicating a lower rate of inclusion in the products tested. The seven peptides were derived from three 75K γ-secalins (E5KZQ2, E5KZQ5, and E5KZQ6). All seven RPMs were detected in BC3, and only RPM-2, the most sensitively detected peptide (area 3e^5^), allowed detection in the food products, BC6, PD1, and PD2.

**Figure 4:**
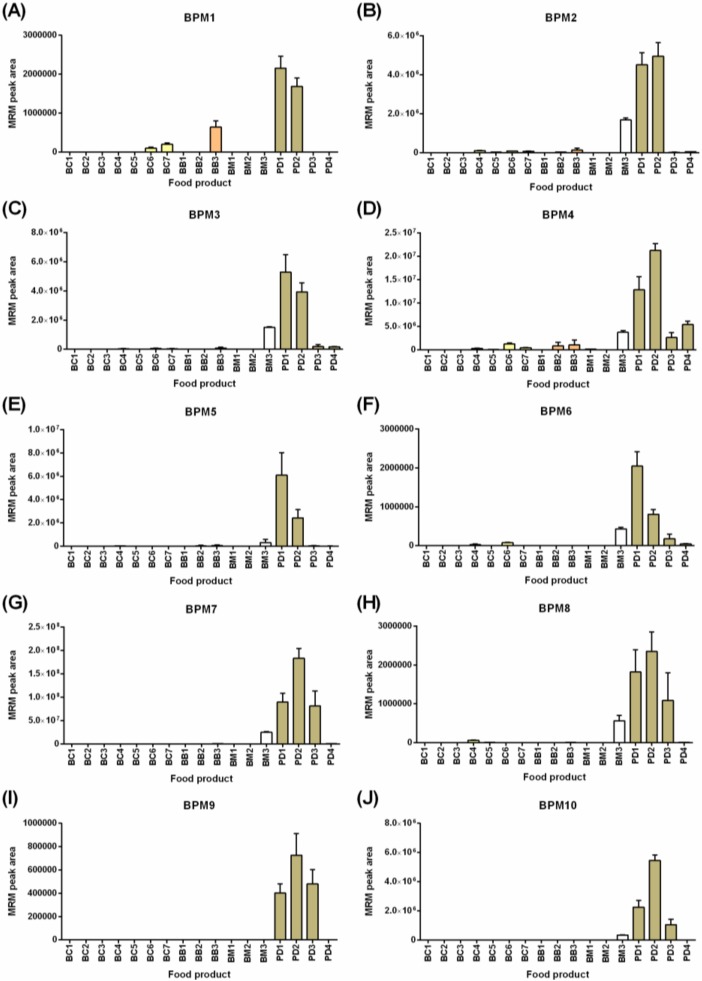
LC-MRM-MS analysis of 10 barley peptide markers (BPMs) across a range of commercial Australian breakfast food products. The peak area + SD is plotted (*n* = 4). Panels (**A**) through (**J**) refer to the ten BPMs defined in Table S12.

Figure 4 shows the detection of the ten selected barley peptide markers (BPMs) in the food products tested with the highest levels noted in the powdered drinks and breakfast milk (BM3) correlating with barley as primary ingredients in these products. The gluten families detected included the avenin-like A proteins (ALP, Figure 4A); B-hordeins (Figure 4B–F); D-hordein (Figure 4G–H); and γ3-hordeins (Figure 4I,J). Greater variation in the pattern of detection of the barley peptide markers was noted than was seen for the panel of wheat peptide markers. All ten BPMs were detected in powdered drinks (PD1 and PD2), but in other products, the number of peptide markers detected ranged from one to nine. Barley was detected in all four breakfast cereals by 4–7 BPMs. This was consistent with barley malt extract being named as an ingredient as is typically used in cereals for imparting flavor and/or color. Analysis of both BB2 and BM2 (products containing wheat and oats) also revealed barley as an unnamed ingredient with four peptide markers detected in BB2 and an average barley composition of ~0.6–0.8% relative to the products tested with the highest response (barley-containing powdered drinks). The inconsistent detection of all peptide markers across the products could be the result of (i) different barley varieties (or extracts) used as the starting ingredient, or (ii) modification to the proteins and hence peptides during the food manufacturing processes. This indicates that accurate detection of barley gluten requires a panel approach utilizing multiple markers rather than reliance on a single peptide marker. The breakfast cereals and breakfast bars showed lower levels of many of the barley peptide markers, with the ALP peptide (BPM1) detected at higher relative levels in BC3-BC7 and BB3 compared to the two powdered drinks (PD1, PD2), indicating that the more soluble ALPs might persist in barley extracts. In fact, barley was uniquely detected in one of the 17 food products (BC5) using BPM1. The combination of five barley peptide markers (BPM1–BPM4 and BPM7) was proposed as the minimum panel to qualitatively detect barley in all products tested. It should be noted that across the range of breakfast cereals, bars, and in breakfast milk (BM3), the ingredient was noted as barley malt extract rather than whole-grain, which might explain some of the variations noted. The last observation was that the matrix of milk resulted in a retention time shift of 0.2–0.3 min earlier. Despite this, the order of intensity of the transitions remained consistent, and the consistent shift of all peptides provided evidence of their correct assignment.

**Figure 5:**
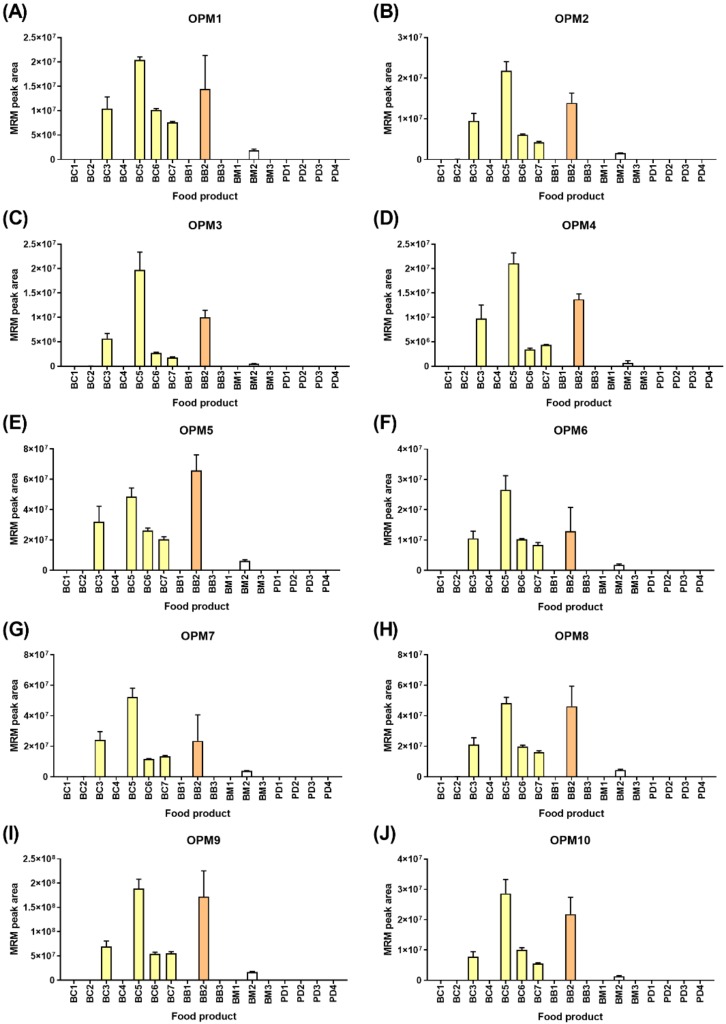
LC-MRM-MS analysis of 10 oat peptide markers (OPMs) across a range of commercial Australian breakfast food products. The peak area + SD is plotted (*n* = 4). Panels (**A**) through (**J**) refer to the ten OPMs defined in Table S12.

Figure 5 shows the detection of the ten oat peptide markers (OPMs) in the food products, wherein all ten peptide markers were derived from avenins. The detection of oats correlated with all products, wherein oats were a named ingredient with lower levels noted in breakfast milk BM2, where the named ingredient was oat flavors rather than whole grain. All ten peptide markers were consistently detected across the six oat-containing food products. Additionally, 9/10 OPMs were detected at consistently low values in BC2 (range 0.07–0.16% relative to BC5). Likewise, lower levels of 3/10 peptide markers were detected in the breakfast bar BB3 that did not specify oats as an ingredient. As was the case with wheat, the pattern of peptide marker detection was highly similar. Two-way ANOVA revealed no significant differences based on the peptide marker (0.19% of the variation, *p* = 0.41), indicating that the ten selected peptide markers were not extensively modified or degraded during processing.

The same commercial food products were tested by sandwich ELISA (Table 1). In agreement, the two breakfast cereals (BC1, BC2) for which there were no significant levels of wheat, rye, or barley by LC-MS yielded values below the lower limit of quantitation (LLOQ) by ELISA. All of the wheat-containing breakfast cereals (BC3–BC7) and breakfast bars (BB2–BB3) yielded gluten measurements above the upper limit of quantitation (ULOQ, >80 mg/kg), as would be expected. Similarly, the barley-containing powdered drinks (PD1, PD2) read above the ULOQ, and PD3 gave an ELISA measurement of 72 mg/kg and moderate levels of barley. The measurement of gluten content in PD4 did not reflect the pattern of LC-MS detection, wherein the levels of barley were very low in PD4, yet ELISA yielded a value >80 mg/kg. As discussed above, the breakfast bar (BB1) labeled “gluten-free” for which no gluten was detected by LC-MS yielded an ELISA response equating to ~14 mg/kg. While this measurement would be considered gluten-free in some jurisdictions (USA, Europe), this result would be contradictory to Australian labeling legislation. The incongruous results from LC-MS and ELISA indicated that the latter measurement might, in fact, be a false positive. The level of barley detected in BM3 (a breakfast milk product) was on average similar to PD3; however, the ELISA measurement was notably lower (~32 mg/kg *cf* 72 mg/kg), indicating that the presence of milk, lipids, or other ingredients might interfere with antibody-antigen binding.

**Figure 6:**
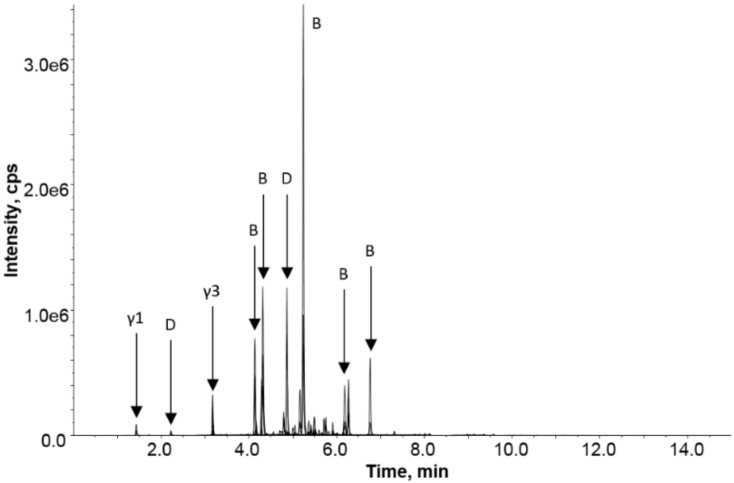
LC-MRM-MS analysis of the savory spread (SS) reveals the detection of barley, as expected, based on barley malt extract—A primary ingredient. The extracted ion chromatogram for barley peptide markers is depicted as intensity (counts/s) versus retention time (min).

A question often raised in the community is the safety of products, such as yeast extracts, that are commonly raised on wheat or barley. To address this question, a targeted proteomic analysis of a diluted extract of a savory spread was undertaken. Figure 6 shows the LC-MRM-MS chromatogram, revealing the detection of barley gluten in the savory spread. Most classes of barley gluten were detected after trypsin digestion with strong responses noted for a range of B-hordeins and the single D-hordein and lower responses for the γ-hordeins. It should be noted that the data presented is not quantitative, owing to the different response factors of the detected peptides, but it qualitatively shows that all protein families were detectable in the product. The savory spread contained not only barley malt extract as an ingredient but also yeast that had been grown on barley and wheat. The data collected did not reveal any unique wheat peptide markers, although several of the barley peptides detected could be commonly found in barley and wheat. 

Over 1000 peptide epitopes, derived from 72 Coeliac-inducing proteins, that elicit CD or activate major histocompatibility complex (MHC) class II-restricted T cells in people with CD, have been extracted from the literature and are presented in an online searchable database [25]. In the current study, peptides, such as RPLFQLVQGQGIIQPQQPAQLEVIR from γ-gliadin (*Triticum aestivum*, B6UKP1) were identified in breakfast cereals (BC), breakfast bars (BB), and powdered drinks (PD). The sequence comprises a known immunotoxic epitope (indicated by underlined sequence, i.e., VQGQGIIQPQQPAQL). Another example of a T cell stimulatory peptide [26] was the identification of QQPGQGQQPEQGQQPGQGQQGYYPTSPQQPGQGK, derived from high molecular weight glutenin subunit (*T. aestivum*, W6AX70) in both wheat-containing BCs and BBs. Another example was the identification in the breakfast cereals of gliadin/avenin-like seed protein (*T. aestivum*, D2KFH0, SDQPQQSFPQQPQQK) that contained the immunotoxic epitope found in γ1-secalin [27].

In this study, a total of 440 gluten peptides were identified across a diverse range of breakfast food products that contained wheat, barley, oats, and/or rye. Discovery proteomics allowed the detection of several CD-toxic peptide epitopes present in gliadin, and targeted proteomics was shown to be a useful tool for detecting the presence of gluten in heavily processed food products. The consistency of detection (pattern of expression and technical variation <15%) of the ten wheat and ten oat peptide markers implied that the markers selected were suitable as proxies for gluten detection. It should be noted that the detection of the WPMs was primarily within dried, baked goods wherein wheat was a primary ingredient. Likewise, the OPMs were mostly found in the same goods category, with only one milk product (BM2) showing high levels of oats. The pattern of detection of the BPMs was more variable, but these were also present in the milk product BM3 or in the powdered drinks that notably contained higher levels of oil and or cocoa that may impact their detection. Moreover, barley is commonly used as a malt extract rather than whole-grain, which could also affect protein conformation and/or accessibility as malting can involve protease release and protein degradation. Future studies should focus on incurring particular classes of food with the raw ingredients to determine the effects of each matrix on peptide marker detection. Nevertheless, this study has highlighted the utility of LC-MS for the detection of wheat, rye, barley, and oats in finished, commercial food products.

## 3. Materials and Methods 

### 3.1. Food Products

Eighteen food products that might be considered typical of an Australian breakfast were obtained from local supermarkets and included 7 breakfast cereals (BC), 3 breakfast bars (BB), 3 breakfast milks (BM), 4 powdered drinks (PD), and a single savory spread (SS). Of these, two were GF (BB1 and BM1), and 16 contained either wheat, rye, barley, or oats. The ingredients list for the food products tested is presented as Supplementary Table S6.

### 3.2. Gluten Extraction and Protein Digestion

The dried products, breakfast cereals, and breakfast bars were ground to a fine powder in a clean, sterile mortar and pestle. After grinding, 0.1 g of each dried product and powdered drink were weighed (*n* = 4 technical replicates). To each sample, 1 mL of 55% isopropyl alcohol (IPA), 2% dithiothreitol (DTT) was added and incubated at 50 °C for 30 min to extract gluten. Gluten digestion was undertaken, as described previously [28]. In brief, 100 µL of gluten extract was diluted in 100 µL of 8 M urea, 100 mM Tris-HCl, pH 8.5 (UA buffer) and loaded onto a 10 kDa molecular weight cut-off (MWCO) centrifugal filter (Merck Millipore, Sydney, Australia) and centrifuged at 20,800× *g* for 15 min at room temperature (RT). The filter (and protein >10 kDa) was washed with 200 µL of UA buffer and centrifuged at 20,800× *g* for 15 min at RT. For cysteine alkylation, iodoacetamide (IAM) (100 µL, 50 mM IAM in UA buffer) was added and incubated in the dark for 20 min at RT before centrifugation (20,800× *g*, 15 min) with two subsequent wash/centrifugation steps with 200 µL of 50 mM ammonium bicarbonate. The trypsin (sequencing grade, Promega, Alexandria, Australia) solution (200 µL, 10 µg/mL in 50 mM ammonium bicarbonate and 1 mM CaCl_2_) was loaded onto the filter and incubated for 18 h at 37 °C in a wet chamber. The tryptic peptides were collected by centrifugation (20,800× *g*, 15 min) followed by an additional wash with 200 µL of 50 mM ammonium bicarbonate and 1 mM CaCl_2_. The combined filtrates were lyophilized and stored at −20 °C.

The savory spread was prepared by weighing 0.4 g spread (*n* = 4 technical replicates), which was dissolved in 400 µL of 50 µM ammonium bicarbonate with vortex mixing and sonication for 5 min at room temperature. Of this solution, 100 µL was taken and diluted in 100 µL of 50 µM ammonium bicarbonate. The breakfast milks (BM, *n* = 4 technical replicates) were prepared by taking 100 µL of milk after thorough mixing and were diluted in 100 µL of 50 mM ammonium bicarbonate. Both the BMs and SS were processed, as described previously [29], wherein 200 µL of the solution was loaded onto a 10 kDa filter, centrifuged at 20,800× *g* for 15 min at room temperature (RT). The filters (and protein >10 kDa) were washed three times with wash buffer (200 µL of 50 mM ammonium bicarbonate) with centrifugation (20,800× *g*, 15 min). The BM/SS were reduced (200 µL, 10 mM DTT) with incubation for 60 min at 25 °C before centrifugation (20,800× *g*, 15 min). For cysteine alkylation, 50 mM iodoacetamide (IAM) was added and incubated in the dark (20 min, 25 °C) before centrifugation (20,800× *g*, 15 min) and two wash/centrifugation steps. The filters were transferred to fresh tubes, and trypsin (Promega, Alexandria, Australia) (200 µL, 25 µg/mL in 50 mM ammonium bicarbonate, 1 mM CaCl_2_) was added and incubated (37 °C, 18 h). The filtrates (digested peptides) were collected by centrifugation (20,800× *g*, 15 min) and washed. The combined filtrates were lyophilized and stored at −20 °C.

### 3.3. Proteomic Profiling

The digested peptides were reconstituted in 50 µL of 1% formic acid (FA), and chromatographic separation (2 μL) on an Ekspert nanoLC415 (Eksigent, Dublin, CA, USA) directly coupled to a 6600 TripleTOF MS (SCIEX, Redwood City, CA, USA). The peptides were desalted for 5 min on a ChromXP C18 (3 μm, 120 Å, 10 mm × 0.3 mm) trap column at a flow rate of 10 μL/min 0.1% FA and separated on a ChromXP C18 (3 μm, 120 Å, 150 mm × 0.3 mm) column at a flow rate of 5 μL/min. A linear gradient from 3–25% solvent B over 38 min was employed followed by 5 min from 25% B to 32% B; 2 min 32% B to 80% B; 3 min at 80% B; 1 min at 80–3% B; and 8 min re-equilibration. The solvents were: (A) 5% DMSO, 0.1% FA, 94.9% water; (B) 5% DMSO, 0.1% FA, 90% acetonitrile, 4.9% water. The instrument parameters were: ion spray voltage 5500 V, curtain gas 25 psi, GS1 12 psi, and GS2 15 psi, heated interface 200 °C. Data were acquired in information-dependent acquisition (IDA) mode comprising a time-of-flight (TOF)-MS survey scan followed by 30 MS/MS, each with a 40 ms accumulation time. The first stage MS analysis was performed in positive ion mode, mass range *m/z* 400−1250, and 0.25 s accumulation time. Tandem mass spectra were acquired on precursor ions >150 counts/s with charge state 2−5 and dynamic exclusion for 15 s with a 100 ppm mass tolerance. Spectra were acquired over the mass range of *m/z* 100−1500 using the manufacturer’s rolling collision energy (CE) based on the size and charge of the precursor ion. Protein identification was undertaken using ProteinPilot™ 5.0 software (SCIEX) with searches conducted against the Poaceae subset of the Uniprot database appended with a contaminant database (Common Repository of Adventitious Proteins). Protein identification reports, based on a combined database search of all samples, were exported to Excel: Supplementary Tables S1, breakfast cereals; S2, breakfast bars; S3, breakfast milks; S4, powdered drinks; and S5, savory spread.

### 3.4. Confirmation of Prototypic Peptides for Food Products

The gluten derived peptides identified are listed in the Supplementary Tables (S7, breakfast cereals; S8, breakfast bars; S9, breakfast milks; S10, powdered drinks; and, S11, savory spread). From the identified peptides, those that were fully tryptic, contained no unusual cleavages and/or modifications (oxidation of Met and pyroglutamination of N-terminal Gln were allowed) and that showed high response in the MS (as judged by peak intensity) were selected for multiple reaction monitoring (MRM) method analysis to confirm the detection of cereal gluten in the samples. Three MRM transitions were used per peptide, and 37 peptide sequences were analyzed (Supplementary Table S12): 10 from wheat [19]; 7 from rye [23]; 10 from barley [17]; 10 from oats [24]. In the case where peptides contained modifiable amino acids (Met, N-terminal Gln), modified versions were also included, and the peak areas of the unmodified/modified forms were summed.

### 3.5. Targeted MS

Reduced and alkylated tryptic peptides (10 μL) were chromatographically separated on a Shimadzu Nexera UHPLC and analyzed on a QTRAP 6500 mass spectrometer (SCIEX), as described previously [30]. Relative quantitation was done using scheduled MRM scanning experiments with a 40 s detection window around the expected retention time (RT) and a 0.3 s cycle time. Peaks were integrated using MultiQuant v3.0 (SCIEX), wherein all three transitions were required to co-elute with a signal-to-noise (S/N) >5 and intensity >1000 counts per second (cps) for detection. Data were acquired using Analyst v1.5 software (SCIEX), and peak areas of the three MRM transitions were accomplished using MultiQuant v2.0.2 software. Graphs were generated in GraphPad Prism v6 (San Diego, CA, USA).

### 3.6. ELISA Measurement

The same set of samples were analyzed by sandwich ELISA using the Ridascreen Gliadin (R-Biopharm, Darmstadt, Germany). The analytical protocols provided by the kit manufacturer were strictly followed. Each of the samples was extracted and measured in duplicate on a single ELISA plate alongside the supplied standards (representing a gluten concentration of 5–80 mg/kg). The data analyses were performed using Microsoft Excel and Graphpad Prism. The results of absorbance readings were analyzed according to the kit manufacturer’s instructions using cubic polynomial regression for the standard curve. 

## 4. Conclusions

In this study, we identified and quantified gluten peptides from wheat, rye, barley, and oats across a diverse range of food products. Several peptide fragments comprising known immunotoxic epitopes were found in breakfast cereals, breakfast bars, and powdered drinks. A comparison to ELISA showed similar results for the wheat-containing products, yet inconsistencies were noted for the barley-containing ingredients. LC-MRM-MS was found to be a specific and reliable tool for the detection of gluten in food products that have been through extensive and diverse processing and exist in different forms (baked goods, powdered drinks, and beverages). 

## Figures and Tables

**Table 1:** ELISA measurement of commercial food products.

Sample ^a^	GF Label	Wheat ^b^	Rye ^b^	Barley ^b^	Oats ^b^	ELISA ^c^ (mg/kg)
BC1	No	ND	ND	ND	ND	1.4
BC2	No	+	ND	ND	+	2.3
BC3	No	+++	++++	+	+++	>80
BC4	No	+++	ND	+	ND	>80
BC5	No	+	ND	+	++++	>80
BC6	No	++++	+	++	+++	>80
BC7	No	++++	ND	+	+++	>80
BB1	Yes	ND	ND	ND	ND	14
BB2	No	++	ND	+	++++	>80
BB3	No	+++	ND	++	+	>80
BM1	Yes	ND	ND	ND	ND	0.7
BM2	No	ND	ND	+	++	7.6
BM3	No	ND	ND	++	+	31.9
PD1	No	ND	+	++++	ND	>80
PD2	No	ND	+	++++	ND	>80
PD3	No	ND	ND	++	ND	71.9
PD4	No	+	ND	+	ND	>80

^a^ The sample types are: BC, breakfast cereal; BB, breakfast bar; BM, breakfast milk, PD, powdered drink; SS, savory spread; GF, gluten-free. ^b^ ND refers to nil detected; + refers to trace level detection, equivalent to <5% of maximum signal detected; ++ refers to low level detection, equivalent to 5–25% of maximum signal detected; +++ refers to medium level detection, equivalent to 25–50% of maximum signal detected; ++++ refers to high level detection equivalent to >50% of maximum signal detected. ^c^ ELISA lower limit of quantitation (LLOQ) is 5 mg/kg and upper limit of quantitation (ULOQ) is 80 mg/kg.

## Supplementary Materials

The following are available online at https://www.mdpi.com/1420-3049/24/20/3665/s1, Table S1: Protein identifications in breakfast cereals (BC), Table S2: Protein identifications in breakfast bars (BB), Table S3: Protein identifications in milk-based breakfast drinks (BM), Table S4: Protein identifications in powdered drinks (PD), Table S5: Protein identifications in the savory spread (SS), Table S6: Ingredients and gluten peptide number identified in each food product type, Table S7: Gluten peptides detected in breakfast cereals (BC), Table S8: Gluten peptides detected in breakfast bars (BB), Table S9: Gluten peptides detected in milk-based breakfast drinks (BM), Table S10: Gluten peptides detected in powdered drinks (PD), Table S11: Gluten peptides detected in savory spread (SS), Table S12: MRM transitions for the wheat, rye, barley, and oat peptide markers assessed in food products.
