# Supplementary material for: Assessing the Utility of Multiplexed Liquid Chromatography-Mass Spectrometry for Gluten Detection in Australian Breakfast Food Products
Source: Molecules. 2019 Oct 11;24(20):3665. doi: 10.3390/molecules24203665 (PMC6832297; doi:10.3390/molecules24203665)
Supplement: Supplementary file 1 [file molecules-24-03665-s001.pdf]

Supplementary Table 1. Protein identifications in breakfast cereals (BC). Trypsin digestion peptides were identified after database searching against the Poaceae subset of the Uniprot database appended with the Common Repository of Adventitious Protein (cRAP) database using a 1% global false discovery rate (FDR) threshold.

N	Unused	Total	% Cov (95)	Accession	Name	Species	Peptides (95%)
1	72.79	72.79	44.98	Q8W3W9	Low-molecular-weight glutenin subunit group 3 type II	*T. timopheevii subsp. araraticumcum aestivum*	139
2	63.52	63.52	69.88	A0A1D5YFA7	Beta-amylase	*T. timopheevii subsp. araraticumcum aestivum*	53
3	61.48	61.48	53.11	A2WVB9	Putative uncharacterized protein	*O. sativa subsp. indica*	114
4	56.15	56.15	35.41	W5EST8	Uncharacterized protein	*T. timopheevii subsp. araraticumcum aestivum*	36
5	54.95	54.98	45.97	W6AX70	High molecular weight glutenin subunit	*T. timopheevii subsp. araraticumcum aestivum*	108
6	50.35	50.35	42.82	A0A060MZP1	High molecular weight glutenin subunit	*T. timopheevii subsp. araraticumcum aestivum*	154
7	48.68	48.76	44.79	K7XE90	Alpha-gliadin	*T. timopheevii subsp. araraticumcum aestivum*	49
8	40.87	46.31	52.40	Q0E261	Glutelin	*O. sativa subsp. japonica*	89
9	38.51	44.56	40.00	G4Y3Y0	High-molecular-weight glutenin subunit Bx7.1	*T. timopheevii subsp. araraticumcum aestivum*	91
10	38.34	39.64	44.32	M8CT91	12S seed storage globulin 1	*O. sativa subsp. japonica*	28
11	33.18	33.18	71.07	M8CU50	Uncharacterized protein	*O. sativa subsp. japonica*	70
12	32.70	45.22	41.12	P12615	12S seed storage globulin 1	*A. sativa*	53
13	32.31	44.16	40.89	D2X6D9	Alpha-gliadin	*T. timopheevii subsp. araraticumcum turgidum subsp. durum*	93
14	32.18	32.28	57.64	W5FZ62	Uncharacterized protein	*T. timopheevii subsp. araraticumcum aestivum*	23
15	31.17	31.19	37.16	Q548E8	16 kDa gamma zein	*Zea mays*	37
16	30.19	43.72	51.02	T1T6C4	Glutelin	*O. sativa subsp. indica*	78
17	30.06	30.07	75.60	Q6S5B1	Alpha amylase inhibitor CM3	*T. timopheevii subsp. araraticumcum turgidum subsp. durum*	63
18	28.15	43.03	52.22	Q10JA8	Glutelin	*O. sativa subsp. japonica*	62

19	27.67	50.00	36.90	I6QQ39	Globulin-3A	*T. timopheevii subsp. araraticumcum aestivum*	36
20	27.64	27.65	24.26	K2C1_HUMAN			13
21	27.50	27.50	70.86	X2KYP9	Monomeric alpha-amylase inhibitor	*T. timopheevii subsp. araraticumcum aestivum*	25
22	26.28	28.29	79.31	P16851	Alpha-amylase/trypsin inhibitor CM2	*T. timopheevii subsp. araraticumcum aestivum*	28
23	25.66	27.76	42.60	Q548E9	27 kDa gamma-zein	*Zea mays*	53
24	25.48	25.50	44.04	W8E6F7	Gamma-gliadin	*O. sativa subsp. japonica*	50
25	24.87	41.93	39.08	A9QUS3	High molecular weight glutenin subunit	*T. timopheevii subsp. araraticumcum aestivum*	77
26	24.71	26.86	33.14	Q9ZWJ8	Glutelin	*O. sativa*	22
27	24.01	24.03	33.75	P06676	Zein-alpha 19C1	*Zea mays*	43
28	23.95	23.96	27.55	Q41844	Zein	*Zea mays*	34
29	23.40	23.48	28.05	A0A1D5YEH1	Uncharacterized protein	*T. timopheevii subsp. araraticumcum aestivum*	19
30	22.82	22.87	15.06	A0A1Q1BYA0	Pyruvate, phosphate dikinase	*Zea mays*	11
30	0.00	22.87	15.41	A0A1D6FQG3	Pyruvate orthophosphate dikinase2	*Zea mays*	11
31	22.80	22.85	60.11	Q946W0	15kD beta zein	*Zea mays*	71
32	21.85	21.89	13.11	W5B5R3	Sucrose synthase	*T. timopheevii subsp. araraticumcum aestivum*	12
33	21.35	21.36	23.12	M7YGL9	Alanine aminotransferase 2	*T. timopheevii subsp. araraticumcum urartu*	12
34	21.29	21.30	20.85	Q5NKP6	Starch synthase, chloroplastic/amyloplastic	*Zea mays*	11
35	21.27	21.27	42.78	A8HNF3	Z1A alpha zein protein	*Zea mays*	18
36	21.10	21.11	46.26	Q946Y9	Thaumatin-like protein TLP7	*H. vulgare*	12
37	20.95	20.98	54.23	A0A173DQZ7	Type-b avenin-like protein	*T. timopheevii subsp. araraticumcum aestivum*	42
38	20.72	20.76	21.34	A0A1D6ABF1	Uncharacterized protein	*T. timopheevii subsp. araraticumcum aestivum*	11
39	20.63	20.67	61.06	Q8LKV8	Seed globulin	*O. sativa subsp. japonica*	17

40	20.57	20.61	80.14	C8CAI4	Dimeric alpha-amylase inhibitor	*T. timopheevii subsp. araraticumcum aestivum*	31
41	20.27	38.68	37.37	Q1ZZT4	Low-molecular-weight glutenin subunit	*T. timopheevii subsp. araraticumcum aestivum*	52
42	20.24	20.31	29.61	Q8LK23	Peroxidase	*T. timopheevii subsp. araraticumcum aestivum*	15
43	20.13	20.14	40.00	Q946W1	50kD gamma zein	*Zea mays*	18
44	19.96	24.52	40.55	A0A1P8DT06	Alpha-gliadin storage protein	*T. timopheevii subsp. araraticumcum spelta*	50
45	19.49	19.51	65.56	A0A1B2LQE5	Alpha amylase trypsin inhibitor	*T. timopheevii subsp. araraticumcum spelta*	11
46	19.43	20.11	49.81	B6UKL5	Gamma-gliadin	*T. timopheevii subsp. araraticumcum turgidum*	52
47	19.15	19.22	28.68	B0FRH4	Aspartate aminotransferase	*T. timopheevii subsp. araraticumcum aestivum*	9
48	18.92	29.48	30.73	S4U5H9	High molecular weight glutenin subunit 1Dx	*O. sativa subsp. japonica*	75
49	18.51	18.52	28.73	A0A0E0E950	Uncharacterized protein	*O. meridionalis*	33
50	18.31	18.49	14.57	A0A1D6REH6	Uncharacterized protein	*T. timopheevii subsp. araraticumcum aestivum*	11
51	18.28	18.32	27.63	R9UNY9	Xylanase inhibitor protein	*T. timopheevii subsp. araraticumcum aestivum*	11
52	17.83	17.91	13.49	K1C10_HUMAN			8
53	17.78	17.89	19.16	Q0PG36	Glucose-1-phosphate adenylyltransferase	*T. timopheevii subsp. araraticumcum aestivum*	10
54	17.73	17.80	20.08	Q9M4Z1	Glucose-1-phosphate adenylyltransferase	*T. timopheevii subsp. araraticumcum aestivum*	9
55	17.51	17.58	15.46	K4FHN8	Protein disulfide-isomerase	*O. sativa subsp. japonica*	9
56	17.46	17.54	57.14	A0A0E0Q5N0	Uncharacterized protein	*O. rufipogon*	15
57	16.98	17.02	40.11	Q09114	Avenin-E	*A. sativa*	23
58	16.90	27.92	23.96	C3VN74	Low molecular weight glutenin	*T. timopheevii subsp. araraticumcum aestivum*	63
59	16.87	21.09	42.77	B6DQD5	Gamma-gliadin	*T. timopheevii subsp. araraticumcum spelta*	40
60	16.83	16.92	51.95	TRYP_PIG			60
61	16.63	16.72	18.30	K1C9_HUMAN			10

62	16.25	16.30	64.66	Q8GZB0	Non-specific lipid-transfer protein	*T. timopheevii subsp. araraticumcum aestivum*	22
63	16.14	32.89	30.48	K7WV92	Low molecular weight glutenin subunit	*T. timopheevii subsp. araraticumcum aestivum*	49
64	15.92	15.96	68.82	W4ZP51	Uncharacterized protein	*T. timopheevii subsp. araraticumcum aestivum*	18
65	15.44	21.80	26.02	A0A1D5ZZT8	Uncharacterized protein	*T. timopheevii subsp. araraticumcum aestivum*	17
66	15.20	17.67	31.49	W5I5X1	Uncharacterized protein	*T. timopheevii subsp. araraticumcum aestivum*	12
67	15.06	34.74	13.45	A0A0E0NCE4	Uncharacterized protein	*O. rufipogon*	30
68	14.95	59.58	61.71	A0A1D5XGF4	Beta-amylase	*T. timopheevii subsp. araraticumcum aestivum*	51
69	14.90	14.92	43.28	A0A1D6CWE1	Uncharacterized protein	*T. timopheevii subsp. araraticumcum aestivum*	38
70	14.84	14.88	12.04	Q9SAU8	HSP70	*T. timopheevii subsp. araraticumcum aestivum*	7
71	14.56	14.62	49.54	I7KM78	Gamma-gliadin	*T. timopheevii subsp. araraticumcum aestivum*	24
72	14.29	14.36	18.80	T1MSW5	Uncharacterized protein	*T. timopheevii subsp. araraticumcum urartu*	8
73	14.25	14.29	56.64	A0A1B2LQA9	A. alpha amylase trypsin inhibitor	*A. sativa*	9
74	14.24	14.28	36.70	Q9AXH7	1-Cys peroxiredoxin	*T. timopheevii subsp. araraticumcum turgidum subsp. durum*	7
75	14.17	14.26	#####	P82901	Non-specific lipid-transfer protein 2P	*T. timopheevii subsp. araraticumcum aestivum*	12
76	14.13	14.18	19.48	M8BDX3	Uncharacterized protein	*O. sativa subsp. japonica*	7
77	14.05	14.05	27.06	A8HNN7	Z1D alpha zein protein	*Zea mays*	14
78	14.02	14.29	71.33	Q5W6A3	Os05g0328800 protein	*O. sativa subsp. japonica*	30
79	14.00	18.51	31.25	A0A0E0AHA2	Uncharacterized protein	*O. meridionalis*	23
80	13.64	13.78	25.70	Q7X6I8	OJ000315_02.8 protein	*O. sativa subsp. japonica*	9
81	13.56	13.70	57.05	Q5Z9M9	Os06g0507200 protein	*O. sativa subsp. japonica*	22
82	13.22	13.33	24.86	W5D5L4	Fructose-bisphosphate aldolase	*T. timopheevii subsp. araraticumcum aestivum*	8
83	13.15	13.19	19.67	Q946V2	Legumin 1	*Zea mays*	7

84	13.14	18.56	21.80	Q9SYT3	22kD alpha zein 5	*Zea mays*	23
85	13.11	13.13	36.47	A0A1D5YGW1	Uncharacterized protein	*T. timopheevii subsp. araraticumcum aestivum*	7
86	13.01	19.11	32.16	M8C2Y1	Serpin-Z2B	*O. sativa subsp. japonica*	14
87	12.98	13.03	26.73	C3VNF1	Malate dehydrogenase	*T. timopheevii subsp. araraticumcum aestivum*	7
88	12.96	18.37	43.93	P27919	Avenin	*A. sativa*	23
89	12.88	14.62	34.54	I4EP67	Avenin	*T. timopheevii subsp. araraticumcum spelta*	27
90	12.81	12.83	65.00	Q2XX25	Non-specific lipid-transfer protein	*Zea mays subsp. parviglumis*	9
91	12.74	12.78	42.47	A2Y665	Putative uncharacterized protein	*O. sativa subsp. indica*	14
92	12.47	12.48	40.50	M8ANS4	Avenin-3	*T. timopheevii subsp. araraticumcum urartu*	14
93	12.43	36.19	43.06	B2BZC7	LMW-m glutenin subunit 0154A5-M	*T. timopheevii subsp. araraticumcum aestivum*	58
94	12.42	12.44	71.13	B9VRI3	Alpha-amylase inhibitor CM16 subunit	*T. timopheevii subsp. araraticumcum spelta*	34
95	12.30	12.42	10.94	M7YE46	Vicilin-like antimicrobial peptides 2-2	*T. timopheevii subsp. araraticumcum urartu*	8
96	12.19	12.20	24.72	A0A1D5Z6P0	rRNA N-glycosidase	*T. timopheevii subsp. araraticumcum aestivum*	6
97	12.16	14.39	15.21	A0A1D5VCN0	Uncharacterized protein	*T. timopheevii subsp. araraticumcum aestivum*	7
98	12.00	12.09	46.62	H2DLU3	Puroindoline b	*T. timopheevii subsp. araraticumcum aestivum*	20
99	11.99	12.06	28.71	A0A1D6BR50	Uncharacterized protein	*T. timopheevii subsp. araraticumcum aestivum*	9
100	11.96	28.28	33.33	I0IT55	Alpha/beta-gliadin	*T. timopheevii subsp. araraticumcum aestivum*	30
101	11.96	12.00	59.76	W5D637	Uncharacterized protein	*T. timopheevii subsp. araraticumcum aestivum*	6
102	11.89	13.43	15.12	I1PE38	Uncharacterized protein	*O. glaberrima*	7
103	11.85	12.11	34.14	G1JSL4	Peroxygenase 1	*A. sativa*	7
104	11.82	14.12	64.83	A0A1B2LQE8	A. alpha amylase trypsin inhibitor	*A. sativa*	10
105	11.77	18.57	92.74	Q5MD68	0.19 dimeric alpha-amylase inhibitor	*T. timopheevii subsp. araraticumcum aestivum*	29

106	11.75	11.93	28.38	W5EFT2	Uncharacterized protein	*T. timopheevii subsp. araraticumcum aestivum*	7
107	11.69	11.94	6.99	O04074	Starch branching enzyme 1	*T. timopheevii subsp. araraticumcum aestivum*	6
108	11.62	26.89	79.31	R7W9W1	Alpha-amylase/trypsin inhibitor CM1	*O. sativa subsp. japonica*	34
109	11.56	11.76	54.05	V5RL87	Puroindoline	*T. timopheevii subsp. araraticumcum aestivum*	15
110	11.38	14.23	13.20	Q9FE55	Protein disulfide-isomerase	*T. timopheevii subsp. araraticumcum turgidum subsp. durum*	7
111	11.26	13.47	58.82	A0A1D6C0D3	Uncharacterized protein	*T. timopheevii subsp. araraticumcum aestivum*	11
112	11.25	11.30	57.29	R7WGD7	Uncharacterized protein	*O. sativa subsp. japonica*	7
113	11.12	11.20	22.14	Q6H6C7	Phosphoglycerate kinase	*O. sativa subsp. japonica*	7
114	11.01	11.20	27.63	A0A1D5TYI6	Uncharacterized protein	*T. timopheevii subsp. araraticumcum aestivum*	7
115	11.00	11.04	44.14	M8BAK8	Uncharacterized protein	*O. sativa subsp. japonica*	8
116	10.86	10.89	40.63	I3NM41	Oleosin	*T. timopheevii subsp. araraticumcum aestivum*	14
117	10.68	12.79	61.05	I1PUP1	Uncharacterized protein	*O. glaberrima*	8
118	10.60	11.13	35.48	Q5BLQ9	Grain softness protein-1B1	*T. timopheevii subsp. araraticumcum aestivum*	11
119	10.44	14.53	13.71	A0A0D9WMW0	Sucrose synthase	*O. meridionalis*	10
120	10.40	10.41	43.71	Q41518	Single-stranded nucleic acid binding protein	*T. timopheevii subsp. araraticumcum aestivum*	7
121	10.29	10.36	19.28	W5D4F5	Uncharacterized protein	*T. timopheevii subsp. araraticumcum aestivum*	5
122	10.07	13.96	33.48	B6SII2	Putative uncharacterized protein	*Zea mays*	15
123	10.07	10.07	27.33	I1PEM1	Oleosin	*O. glaberrima*	10
124	10.00	10.00	45.30	M0V3U0	Non-specific lipid-transfer protein	*H. vulgare subsp. vulgare*	5
125	9.92	13.92	69.23	B6SI37	Embryo specific protein1	*Zea mays*	8
126	9.83	11.93	46.15	A0A1B2LQC0	A. alpha amylase trypsin inhibitor	*A. sativa*	7
127	9.80	9.85	15.25	M4Q9V0	Enolase	*T. timopheevii subsp. araraticumcum aestivum*	5

128	9.77	11.85	37.84	Q946V1	Hageman factor inhibitor	*Zea mays*	6
129	9.73	13.38	27.36	W5BB88	Uncharacterized protein	*T. timopheevii subsp. araraticumcum aestivum*	9
130	9.69	9.82	7.59	Q2QV45	70 kDa heat shock protein	*O. sativa subsp. japonica*	6
131	9.67	9.77	11.34	W5D4W6	Uncharacterized protein	*T. timopheevii subsp. araraticumcum aestivum*	6
132	9.53	55.95	52.91	A2Z708	Uncharacterized protein	*O. sativa subsp. indica*	99
133	9.49	9.52	36.84	Q93W25	Peptidyl-prolyl cis-trans isomerase	*T. timopheevii subsp. araraticumcum aestivum*	7
134	9.41	18.80	12.97	I1PVJ3	Pyruvate, phosphate dikinase	*O. glaberrima*	11
135	9.33	21.40	34.94	B6UKW0	Gamma-gliadin	*Aegilops bicornis*	38
136	9.33	9.37	19.02	A0A1D5VIX2	Uncharacterized protein	*T. timopheevii subsp. araraticumcum aestivum*	5
137	9.27	9.34	21.45	A0A1D5XF71	Uncharacterized protein	*T. timopheevii subsp. araraticumcum aestivum*	5
138	9.22	9.25	6.48	M8A571	Elongation factor 2	*T. timopheevii subsp. araraticumcum urartu*	5
139	9.08	9.16	6.39	Q7X834	OSJNBa0019G23.2 protein	*O. sativa subsp. japonica*	6
140	9.05	25.09	45.73	Q9ST58	Serpin-Z1C	*T. timopheevii subsp. araraticumcum aestivum*	17
141	8.98	38.38	40.89	R9XUP7	Alpha-gliadin	*T. timopheevii subsp. araraticumcum aestivum*	78
142	8.82	8.85	35.50	A0A1D6B1J0	Uncharacterized protein	*T. timopheevii subsp. araraticumcum aestivum*	5
143	8.79	8.82	25.23	A8HNP0	Z1D alpha zein protein	*Zea mays*	8
144	8.77	8.93	29.46	W4ZN68	Uncharacterized protein	*T. timopheevii subsp. araraticumcum aestivum*	11
145	8.71	17.91	52.44	Q0Q5D9	Globulin 1	*T. timopheevii subsp. araraticumcum aestivum*	13
146	8.58	27.84	37.26	F8SGQ3	Low-molecular-weight glutenin subunit	*T. timopheevii subsp. araraticumcum aestivum*	75
147	8.45	8.49	14.52	B6TEC1	Sorghum bicolortol dehydrogenase	*Zea mays*	6
148	8.39	31.59	57.04	A0A1D5ZBL7	Uncharacterized protein	*T. timopheevii subsp. araraticumcum aestivum*	21
149	8.35	8.37	16.02	I1P1X6	Glyceraldehyde-3-phosphate dehydrogenase	*O. glaberrima*	5

150	8.26	9.95	63.40	A1YQF0	Os05g0331532 protein	*O. sativa subsp. japonica*	21
151	8.24	38.65	31.88	Q38780	11S globulin	*A. sativa*	54
152	8.18	8.20	56.34	P81713	Bowman-Birk type trypsin inhibitor	*T. timopheevii subsp. araraticumcum aestivum*	8
153	8.16	60.72	62.91	W5EKI0	Beta-amylase	*T. timopheevii subsp. araraticumcum aestivum*	57
154	8.16	8.28	4.37	Q9FUU7	Starch branching enzyme 2	*T. timopheevii subsp. araraticumcum aestivum*	4
155	8.12	8.13	15.76	W5AC28	Uncharacterized protein	*T. timopheevii subsp. araraticumcum aestivum*	4
156	8.10	10.18	28.64	G8ZCW5	Avenin protein	*T. timopheevii subsp. araraticumcum spelta*	15
157	8.09	8.21	15.63	W5BE38	Oleosin	*T. timopheevii subsp. araraticumcum aestivum*	4
158	8.07	12.91	31.63	A0A0K2QJC8	Alpha/beta-gliadin	*T. timopheevii subsp. araraticumcum aestivum*	16
159	8.02	8.13	7.22	A0A1D5ST13	Uncharacterized protein	*T. timopheevii subsp. araraticumcum aestivum*	4
160	8.00	45.26	35.53	Q6WZC3	Low molecular weight glutenin subunit	*T. timopheevii subsp. araraticumcum aestivum*	60
161	8.00	12.00	59.76	M7ZCN8	Uncharacterized protein	*T. timopheevii subsp. araraticumcum urartu*	6
162	7.96	9.85	63.51	A0A1B2LQD4	A. alpha amylase trypsin inhibitor-2	*A. sativa*	7
163	7.93	8.00	30.91	Q8H0B8	Cold regulated protein	*T. timopheevii subsp. araraticumcum aestivum*	4
164	7.86	10.62	47.44	Q0IP02	Os12g0269200 protein	*O. sativa subsp. japonica*	40
165	7.81	7.86	27.04	A0A1D5SVU1	Uncharacterized protein	*T. timopheevii subsp. araraticumcum aestivum*	4
166	7.72	17.75	60.56	Q41888	Prolamin PPROL 17	*Zea mays*	54
167	7.69	7.79	11.14	W5FAY5	Uncharacterized protein	*T. timopheevii subsp. araraticumcum aestivum*	4
168	7.60	11.77	11.73	J3L8Y0	Uncharacterized protein	*O. brachyantha*	7
169	7.59	7.63	12.77	M8BYD7	Purple acid phosphatase 3	*O. sativa subsp. japonica*	4
170	7.45	7.52	13.28	Q9XF30	Putative uncharacterized protein	*O. sativa subsp. indica*	5
171	7.43	37.88	37.57	A0A1D5S0Z9	Uncharacterized protein	*T. timopheevii subsp. araraticumcum aestivum*	27

172	7.40	41.77	27.78	B7U6L5	Globulin 3B	*T. timopheevii subsp. araraticumcum aestivum*	26
173	7.40	7.44	36.90	A3KLI1	Dehydrin DHN1	*Zea mays*	6
174	7.39	7.43	37.09	Q7XC37	Nucleoside diphosphate kinase	*O. sativa subsp. japonica*	5
175	7.37	11.58	12.16	Q9ARI0	ADP-glucose pyrophosphorylase large subunit isoform	*O. sativa subsp. japonica*	6
176	7.33	7.50	24.18	A0A1B5GE57	Caleosin	*T. timopheevii subsp. araraticumcum aestivum*	4
177	7.28	10.96	59.60	B6UKM7	Gamma-gliadin	*T. timopheevii subsp. araraticumcum aestivum*	26
178	7.25	9.85	32.26	A0A0E0Q5F7	Uncharacterized protein	*O. rufipogon*	22
179	7.25	7.33	10.67	A7UME2	Xylanase inhibitor 725ACCN	*T. timopheevii subsp. araraticumcum aestivum*	4
180	7.24	7.37	17.76	W5BPU1	40S ribosomal protein SA	*T. timopheevii subsp. araraticumcum aestivum*	4
181	7.14	7.28	28.99	T1MEJ3	Uncharacterized protein	*T. timopheevii subsp. araraticumcum urartu*	5
182	7.09	7.13	10.79	I1HXL8	Tubulin beta chain	*Brachypodium distachyon*	4
183	7.01	7.05	15.83	Q6JBL5	Chitinase	*Zea mays subsp. parviglumis*	4
184	6.95	9.10	18.04	A0A0D3HQM8	Non-specific lipid-transfer protein	*O. meridionalis*	9
185	6.87	46.07	34.66	R9XVA5	LMW-GS	*T. timopheevii subsp. araraticumcum aestivum*	86
186	6.83	15.42	52.94	A0A1B2LQD8	A. alpha amylase trypsin inhibitor	*A. sativa*	9
187	6.79	8.72	13.95	W5BUF4	Caleosin	*T. timopheevii subsp. araraticumcum aestivum*	4
188	6.76	6.82	14.06	W5IA32	Formate dehydrogenase, mitochondrial	*T. timopheevii subsp. araraticumcum aestivum*	4
189	6.75	11.67	67.24	M8BYH8	Non-specific lipid-transfer protein	*O. sativa subsp. japonica*	16
190	6.71	6.79	33.77	B2FH40	16.9a kDa heat-shock protein	*T. timopheevii subsp. araraticumcum spelta*	4
191	6.67	11.24	36.27	V5M3L1	Avenin-like protein	*T. timopheevii subsp. araraticumcum aestivum*	20
192	6.66	6.70	7.47	W5FWT6	Aldehyde dehydrogenase 7B1	*T. timopheevii subsp. araraticumcum aestivum*	3
193	6.58	6.61	10.90	A0A1D5ZX81	Uncharacterized protein	*T. timopheevii subsp. araraticumcum aestivum*	4

194	6.48	31.96	37.98	A0A1P8DT36	Alpha-gliadin storage protein	*T. timopheevii subsp. araraticumcum spelta*	40
195	6.47	6.49	32.14	Q01I75	OSIGBa0101P20.7 protein	*O. sativa*	3
196	6.34	15.82	21.49	Q7XYX4	High-molecular-weight glutenin x-type subunit	*Elymus elongatus*	43
197	6.31	6.33	6.48	W5E0G4	Uncharacterized protein	*T. timopheevii subsp. araraticumcum aestivum*	4
198	6.30	13.80	28.07	Q38769	Permatin	*A. sativa*	7
199	6.26	13.08	13.46	Q9S768	Alanine aminotransferase	*O. sativa*	6
200	6.26	10.38	63.64	Q41540	CM 17 protein	*T. timopheevii subsp. araraticumcum aestivum*	25
201	6.24	25.72	59.28	M8BV45	Alpha-amylase/trypsin inhibitor CM3	*O. sativa subsp. japonica*	54
202	6.22	6.34	22.79	Q53WS1	Alpha 1 purothionin	*T. timopheevii subsp. araraticumcum aestivum*	5
203	6.20	14.23	68.82	Q53WS3	Em protein	*T. timopheevii subsp. araraticumcum aestivum*	11
204	6.20	13.06	56.40	D2KFH1	Avenin-like a4	*T. timopheevii subsp. araraticumcum aestivum*	24
205	6.18	9.86	35.37	Q9FVJ4	CDS_GSP-1	*O. sativa subsp. japonica*	10
206	6.18	8.23	22.82	Q946V3	Alpha globulin	*Zea mays*	4
207	6.17	6.32	8.70	M8B2K7	ATP synthase subunit beta	*O. sativa subsp. japonica*	3
208	6.14	10.49	45.80	A0A1D5XMK2	Uncharacterized protein	*T. timopheevii subsp. araraticumcum aestivum*	8
209	6.10	32.56	37.50	A0A0K2QJA2	Alpha/beta-gliadin	*T. timopheevii subsp. araraticumcum aestivum*	48
210	6.08	6.14	17.29	CAS1_BOVIN			4
211	6.06	8.08	38.55	A0A1D5Y5R8	Uncharacterized protein	*T. timopheevii subsp. araraticumcum aestivum*	5
212	6.05	10.16	43.84	A0A1D6DC72	Uncharacterized protein	*T. timopheevii subsp. araraticumcum aestivum*	17
213	6.04	12.50	52.50	Q40653	Allergenic protein	*O. sativa subsp. japonica*	27
214	6.03	6.08	25.22	A0A1D6ACI6	Uncharacterized protein	*T. timopheevii subsp. araraticumcum aestivum*	3
215	6.02	12.43	38.30	H9A6C4	Gamma-gliadin	*O. sativa subsp. japonica*	16

216	6.01	6.01	28.77	A0A1D5SGZ4	Uncharacterized protein	*T. timopheevii subsp. araraticumcum aestivum*	3
217	6.00	12.60	64.58	Q9FEK9	Lipid transfer protein	*T. timopheevii subsp. araraticumcum turgidum subsp. durum*	12
218	6.00	8.00	26.74	P21641	Oleosin Zm-II	*Zea mays*	4
219	6.00	7.85	50.53	M8BVH7	Putative non-specific lipid-transfer protein	*O. sativa subsp. japonica*	6
220	6.00	6.00	31.30	Q38770	Type V Thionin	*O. sativa subsp. japonica*	4
221	6.00	6.00	26.90	A0A1D6B171	Uncharacterized protein	*T. timopheevii subsp. araraticumcum aestivum*	4
222	6.00	6.00	16.51	A8HNN2	Z1D alpha zein protein	*Zea mays*	5
223	6.00	6.00	20.28	W5D5Z6	Uncharacterized protein	*T. timopheevii subsp. araraticumcum aestivum*	3
224	5.98	7.92	12.10	W5EI90	Uncharacterized protein	*T. timopheevii subsp. araraticumcum aestivum*	4
225	5.97	6.06	27.91	M7ZWD9	Nuclear transport factor 2	*T. timopheevii subsp. araraticumcum urartu*	3
226	5.96	20.68	38.29	A0A0S2GJR0	Low-molecular-weight glutenin subunit	*T. timopheevii subsp. araraticumcum aestivum*	53
227	5.91	33.77	51.96	K7X1L1	Alpha-gliadin	*T. timopheevii subsp. araraticumcum aestivum*	40
228	5.89	5.97	16.92	A0A1D5UB33	Uncharacterized protein	*T. timopheevii subsp. araraticumcum aestivum*	3
229	5.88	5.95	16.89	M8BUR6	Uncharacterized protein	*O. sativa subsp. japonica*	5
230	5.80	22.05	18.74	J9Q8Q6	High molecular weight glutenin subunit 1Ay protein	*T. timopheevii subsp. araraticumcum dicoccoides*	38
231	5.77	5.85	7.43	A0A077RS68	Uncharacterized protein	*T. timopheevii subsp. araraticumcum aestivum*	3
232	5.65	14.54	91.13	A4GG11	Dimeric alpha-amylase inhibitor	*Aegilops longissima*	26
233	5.64	5.70	23.21	Q68HV3	LEA protein 12	*O. sativa subsp. japonica*	3
234	5.54	5.62	23.21	F2DWT1	Predicted protein	*H. vulgare subsp. vulgare*	3
235	5.51	23.66	24.85	Q6ESW6	Glutelin	*O. sativa subsp. japonica*	40
236	5.47	5.55	19.67	D2KFH0	Gliadin/avenin-like seed protein	*T. timopheevii subsp. araraticumcum aestivum*	3

237	5.45	5.84	4.73	Q9AUV8	Alpha-1,4 glucan phosphorylase	*O. sativa subsp. japonica*	6
238	5.38	5.46	13.02	W5ERW2	Uncharacterized protein	*T. timopheevii subsp. araraticumcum aestivum*	3
239	5.37	11.24	28.32	W5DWP8	Uncharacterized protein	*T. timopheevii subsp. araraticumcum aestivum*	7
240	5.34	5.48	4.89	W5AC96	Carboxypeptidase	*T. timopheevii subsp. araraticumcum aestivum*	3
241	5.21	11.72	22.72	H6ULJ1	75k gamma secalin	*Secale strictum*	12
242	5.18	12.07	26.56	B6UGP0	Putative uncharacterized protein	*Zea mays*	20
243	5.18	7.32	46.94	R4I506	Vromindoline 1.3	*A. sativa*	10
244	5.09	10.30	58.82	Q6L5L7	Embryonic abundant protein	*Bromus inermis*	7
245	5.04	5.11	11.30	Q2XXG4	Ribosome inactivating protein 1	*Zea mays subsp. parviglumis*	3
246	4.98	5.12	13.75	M7Z0X1	Aldose reductase	*T. timopheevii subsp. araraticumcum urartu*	3
247	4.96	5.24	4.72	A0A1D6LPR1	Glycosyl hydrolase family 31 protein	*Zea mays*	3
248	4.95	5.04	27.10	W4ZXB7	Uncharacterized protein	*T. timopheevii subsp. araraticumcum aestivum*	3
249	4.93	42.84	41.16	A0A0E3Z7F7	Alpha-gliadin	*T. timopheevii subsp. araraticumcum aestivum*	49
250	4.86	4.92	33.80	W0NU33	Vromindoline VIN3	*T. timopheevii subsp. araraticumcum spelta*	6
251	4.84	4.94	15.75	O64392	T. timopheevii subsp. araraticumcum aestivumwin-1	*T. timopheevii subsp. araraticumcum aestivum*	4
252	4.65	13.57	49.31	A0A1B2LQC2	A. alpha amylase trypsin inhibitor	*A. sativa*	9
253	4.63	10.84	29.09	B7ERQ1	Peroxiredoxin	*O. sativa subsp. japonica*	5
254	4.59	4.62	4.29	A0A1D6D1Q3	Pyrophosphate--fructose 6-phosphate 1-phosphotransferase subunit beta	*T. timopheevii subsp. araraticumcum aestivum*	2
255	4.57	4.58	4.52	W5XK42	ATP synthase subunit alpha	*Aegilops longissima*	2
256	4.53	4.57	19.13	B6TJ90	Histone H4	*Zea mays*	4
257	4.53	4.55	20.98	N1QTW5	Trypsin inhibitor CMc	*O. sativa subsp. japonica*	6

258	4.51	4.54	13.17	A0A1D5ST87	Uncharacterized protein	*T. timopheevii subsp. araraticumcum aestivum*	2
259	4.48	55.05	30.14	D3UAL8	Low molecular weight glutenin subunit B3-3	*T. timopheevii subsp. araraticumcum aestivum*	89
260	4.41	12.36	10.25	W0G9U6	Starch synthase, chloroplastic/amyloplastic	*T. timopheevii subsp. araraticumcum urartu*	5
261	4.40	4.43	5.50	W5FGH0	Uncharacterized protein	*T. timopheevii subsp. araraticumcum aestivum*	2
262	4.38	41.99	38.22	P14812	12S seed storage globulin 2	*A. sativa*	51
263	4.35	10.51	42.32	Q548E7	19kD alpha zein B2	*Zea mays*	11
264	4.28	4.38	8.84	W5AB71	Uncharacterized protein	*T. timopheevii subsp. araraticumcum aestivum*	3
265	4.27	4.33	8.30	W4ZTE4	Uncharacterized protein	*T. timopheevii subsp. araraticumcum aestivum*	2
266	4.26	25.60	40.00	R4JAP5	Low-molecular-weight glutenin subunit	*T. timopheevii subsp. araraticumcum aestivum*	41
267	4.25	4.34	7.50	R9R4F6	Alpha amylase inhibitor	*O. sativa subsp. indica*	2
268	4.24	11.29	44.22	U6A2I2	Vromindoline VIN2	*T. timopheevii subsp. araraticumcum spelta*	12
269	4.23	34.29	24.55	A0A1D5XS09	Uncharacterized protein	*T. timopheevii subsp. araraticumcum aestivum*	24
270	4.23	6.48	22.15	I1QAS5	Nucleoside diphosphate kinase	*O. glaberrima*	3
271	4.22	4.28	18.54	Q0WX48	Thaumatin-like xylanase inhibitor	*T. timopheevii subsp. araraticumcum aestivum*	3
272	4.22	4.24	8.60	R7WEA5	GTP-binding nuclear protein	*O. sativa subsp. japonica*	2
273	4.18	19.15	34.42	Q9ST57	Serpin-Z2A	*T. timopheevii subsp. araraticumcum aestivum*	14
274	4.18	4.20	12.00	W5D067	Uncharacterized protein	*T. timopheevii subsp. araraticumcum aestivum*	2
275	4.17	15.39	40.65	Q94G96	Gamma-gliadin	*T. timopheevii subsp. araraticumcum aestivum*	37
276	4.16	6.25	9.79	A0A0E0P2F5	Uncharacterized protein	*O. rufipogon*	4
277	4.14	17.09	35.92	R9XU99	Gamma-gliadin	*T. timopheevii subsp. araraticumcum aestivum*	23
278	4.13	10.21	40.80	A0A1D6A2L5	Uncharacterized protein	*T. timopheevii subsp. araraticumcum aestivum*	7
279	4.12	30.82	34.95	O49258	12s globulin	*A. sativa*	46

280	4.12	4.13	7.29	M8BK19	Glucan endo-1,3-beta-glucosidase GIV	*O. sativa subsp. japonica*	2
281	4.11	19.03	74.68	W5D003	Uncharacterized protein	*T. timopheevii subsp. araraticumcum aestivum*	21
282	4.11	8.17	15.54	Q8GVI3	Alpha-amylase /trypsin inhibitor	*O. sativa subsp. japonica*	4
283	4.11	4.12	17.41	A0A1D6A149	Uncharacterized protein	*T. timopheevii subsp. araraticumcum aestivum*	3
284	4.10	12.80	27.02	Q41603	LMW glutenin	*T. timopheevii subsp. araraticumcum turgidum subsp. durum*	20
285	4.09	12.36	46.20	B8B8F6	Putative uncharacterized protein	*O. sativa subsp. indica*	10
286	4.09	5.80	6.89	Q7M1Z8	Globulin-2	*Zea mays*	3
287	4.08	10.80	46.82	Q2A780	Putative avenin-like a	*T. timopheevii subsp. araraticumcum spelta*	21
288	4.08	4.09	12.10	W4ZMR5	Uncharacterized protein	*T. timopheevii subsp. araraticumcum aestivum*	2
289	4.06	6.08	14.19	M8B2V2	Uncharacterized protein	*T. timopheevii subsp. araraticumcum urartu*	3
290	4.05	4.12	13.07	P93602	PUP88 protein; member of trypsin/a-amylase inhibitors family from cereals	*T. timopheevii subsp. araraticumcum aestivum*	2
291	4.03	20.29	24.75	A0A1D5Z1A1	Uncharacterized protein	*T. timopheevii subsp. araraticumcum aestivum*	19
292	4.03	4.03	12.95	I1HK96	Uncharacterized protein	*Brachypodium distachyon*	2
293	4.01	11.26	34.76	G8ZCU3	Avenin protein	*T. timopheevii subsp. araraticumcum spelta*	19
294	4.01	4.01	8.82	A0A1D5VJH0	Uncharacterized protein	*T. timopheevii subsp. araraticumcum aestivum*	2
295	4.00	41.32	37.96	Q0Q5D3	Y-type HMW glutenin	*T. timopheevii subsp. araraticumcum aestivum*	72
296	4.00	39.63	32.63	A0A023WGF7	Alpha-gliadin	*T. timopheevii subsp. araraticumcum aestivum*	40
297	4.00	32.19	38.06	K7X0N8	Alpha-gliadin	*T. timopheevii subsp. araraticumcum aestivum*	41
298	4.00	19.97	57.64	Q0Q5E3	Globulin 1	*T. timopheevii subsp. araraticumcum aestivum*	16
299	4.00	19.79	48.44	W5FN32	Uncharacterized protein	*T. timopheevii subsp. araraticumcum aestivum*	11
300	4.00	14.88	11.93	I1HT87	Uncharacterized protein	*Brachypodium distachyon*	7

301	4.00	14.58	51.19	Q2A784	Avenin-like a1	*T. timopheevii subsp. araraticumcum aestivum*	32
302	4.00	14.00	45.63	Q8H4M4	Allergenic protein	*O. sativa subsp. japonica*	10
303	4.00	10.05	30.49	Q9FVJ5	GSP-A1	*T. timopheevii subsp. araraticumcum aestivum*	9
304	4.00	10.01	31.85	I1Q921	Uncharacterized protein	*O. glaberrima*	10
305	4.00	9.68	16.61	Q93V80	Granule-bound starch synthase	*Zea mays*	4
306	4.00	8.41	63.27	Q2PCC3	Type 2 non specific lipid transfer protein	*T. timopheevii subsp. araraticumcum aestivum*	8
307	4.00	7.38	26.67	A0A1D6DG60	Uncharacterized protein	*T. timopheevii subsp. araraticumcum aestivum*	6
308	4.00	7.08	18.32	B4G1G1	Desiccation-related protein PCC13-62	*Zea mays*	6
309	4.00	6.00	31.31	A0A1D6F5A7	Putative non-specific lipid-transfer protein 2	*Zea mays*	5
310	4.00	5.38	12.12	W5GW81	Salt-induced YSK2 dehydrin 3	*T. timopheevii subsp. araraticumcum aestivum*	6
311	4.00	4.00	15.67	W5FAI9	Uncharacterized protein	*T. timopheevii subsp. araraticumcum aestivum*	2
312	4.00	4.00	20.16	W5EC46	Non-specific lipid-transfer protein	*T. timopheevii subsp. araraticumcum aestivum*	2
313	4.00	4.00	13.67	R7W9Z9	Uncharacterized protein	*O. sativa subsp. japonica*	2
314	4.00	4.00	8.43	R7W9M2	Putative aquaporin TIP3-1	*O. sativa subsp. japonica*	2
315	4.00	4.00	2.85	Q8GT15	Os01g0947000 protein	*O. sativa subsp. japonica*	2
316	4.00	4.00	41.94	M8B440	Uncharacterized protein	*O. sativa subsp. japonica*	2
317	4.00	4.00	8.33	K3XYM7	Uncharacterized protein	*Setaria italica*	2
318	4.00	4.00	3.27	F2CSX3	Predicted protein	*H. vulgare subsp. vulgare*	2
319	4.00	4.00	6.75	B4F9T3	Putative uncharacterized protein	*Zea mays*	2
320	4.00	4.00	14.37	A0A1D5ZZ07	Oleosin	*T. timopheevii subsp. araraticumcum aestivum*	4
321	4.00	4.00	49.38	P30569	EC protein I/II	*T. timopheevii subsp. araraticumcum aestivum*	2
322	4.00	4.00	14.74	CASK_BOVIN			2

323	3.90	4.18	20.00	M8CGQ1	Uncharacterized protein	*O. sativa subsp. japonica*	3
324	3.87	4.01	4.83	B6SLF6	Antimicrobial peptide MBP-1	*Zea mays*	3
325	3.85	19.76	36.07	W8Q2K7	Farinin protein	*Brachypodium distachyon*	40
326	3.85	3.92	7.49	B6SI09	Aquaporin TIP3.1	*Zea mays*	2
327	3.83	3.95	16.81	F2D1K9	Predicted protein	*H. vulgare subsp. vulgare*	2
328	3.81	3.97	2.86	W5G4R7	Pyrophosphate--fructose 6-phosphate 1-phosphotransferase subunit alpha	*T. timopheevii subsp. araraticumcum aestivum*	2
329	3.81	3.92	3.89	R7W1A5	Polyadenylate-binding protein	*O. sativa subsp. japonica*	2
330	3.80	10.38	51.77	A3BHT2	Uncharacterized protein	*O. sativa subsp. japonica*	11
331	3.80	8.08	37.42	Q0KIW2	Glycine-rich RNA-binding protein	*T. timopheevii subsp. araraticumcum aestivum*	5
332	3.79	6.30	4.96	K22E_HUMAN			3
333	3.78	3.97	2.49	F2DL45	Predicted protein	*H. vulgare subsp. vulgare*	3
334	3.76	3.87	15.50	A0A1D6LDG1	Seed maturation protein	*Zea mays*	2
335	3.75	3.85	9.33	M8A2L5	Uncharacterized protein	*T. timopheevii subsp. araraticumcum urartu*	2
336	3.68	6.48	19.34	W5AY74	Caleosin	*T. timopheevii subsp. araraticumcum aestivum*	3
337	3.66	3.81	17.32	A0A1D6D5M0	Uncharacterized protein	*T. timopheevii subsp. araraticumcum aestivum*	4
338	3.66	3.77	16.20	Q8RZH0	Putative uncharacterized protein	*O. sativa subsp. japonica*	2
339	3.64	3.80	5.54	R7W8V8	Tubulin alpha chain	*O. sativa subsp. japonica*	2
340	3.63	3.79	18.18	C0PPC9	Uncharacterized protein	*Zea mays*	3
341	3.62	3.83	4.59	W5I0B0	Uncharacterized protein	*T. timopheevii subsp. araraticumcum aestivum*	2
342	3.61	3.79	5.18	Q6Z782	Os02g0202400 protein	*O. sativa subsp. japonica*	2
343	3.59	7.90	16.02	K3YIG5	Glyceraldehyde-3-phosphate dehydrogenase	*Setaria italica*	4

344	3.59	7.59	38.71	A0A1E5VL69	Late embryogenesis abundant protein EMB564	*T. timopheevii subsp. araraticumcum spelta*	4
345	3.54	3.82	16.00	N1QZ03	Uncharacterized protein	*O. sativa subsp. japonica*	3
346	3.54	3.70	5.26	W5GYF2	Uncharacterized protein	*T. timopheevii subsp. araraticumcum aestivum*	2
347	3.51	3.61	4.52	N1QTB1	Vacuolar-sorting receptor 4	*O. sativa subsp. japonica*	2
348	3.44	3.52	26.83	W5EL62	40S ribosomal protein S21	*T. timopheevii subsp. araraticumcum aestivum*	2
349	3.41	3.56	5.16	W5ES41	Uncharacterized protein	*T. timopheevii subsp. araraticumcum aestivum*	2
350	3.33	3.47	6.41	M8BBZ6	Uncharacterized protein	*O. sativa subsp. japonica*	2
351	3.32	3.45	8.15	M8BD70	Ras-related protein RIC1	*O. sativa subsp. japonica*	2
352	3.31	3.42	13.43	A0A1D5T6F6	Uncharacterized protein	*T. timopheevii subsp. araraticumcum aestivum*	2
353	3.31	3.41	7.07	M7ZNI1	Putative calcium-binding protein CML7	*T. timopheevii subsp. araraticumcum urartu*	2
354	3.26	3.36	4.32	W5BKC3	Uncharacterized protein	*T. timopheevii subsp. araraticumcum aestivum*	2
355	3.24	5.66	27.94	B8YM21	Beta purothionin	*T. timopheevii subsp. araraticumcum urartu*	7
356	3.19	4.32	34.44	Q9S6Y2	Alpha purothionin	*T. timopheevii subsp. araraticumcum aestivum*	5
357	3.12	3.21	6.43	A0A1D5WYI6	Uncharacterized protein	*T. timopheevii subsp. araraticumcum aestivum*	2
358	3.11	3.18	17.33	Q6E648	MPI	*Tripsacum dactyloides*	2
359	3.03	3.29	25.44	A0A1D6CR43	Uncharacterized protein	*T. timopheevii subsp. araraticumcum aestivum*	3
360	3.01	3.08	7.02	W5G8P6	Uncharacterized protein	*T. timopheevii subsp. araraticumcum aestivum*	2
361	3.00	3.07	5.66	Q7XLP7	OSJNBa0044M19.9 protein	*O. sativa subsp. japonica*	2
362	3.00	3.06	5.40	J3MTA1	Uncharacterized protein	*O. brachyantha*	2
363	2.99	5.25	7.73	Q03865	Vicilin-like embryo storage protein	*Zea mays*	5
364	2.99	3.06	7.41	C0HH10	Dirigent protein	*Zea mays*	2
365	2.98	3.12	4.33	Q9ZTP0	Putative uncharacterized protein Ose705	*O. sativa*	2

366	2.96	3.03	11.50	J3M9C0	Uncharacterized protein	*O. brachyantha*	2
367	2.91	3.02	25.83	Q8GVK7	13 kDa prolamin	*O. sativa subsp. japonica*	11
368	2.90	3.01	2.21	R4ZAN8	L-2	*T. timopheevii subsp. araraticumcum kiharae*	1
369	2.90	2.97	13.93	I1QLJ5	Uncharacterized protein	*O. glaberrima*	2
370	2.85	16.12	15.32	A0A1D5SU51	Uncharacterized protein	*T. timopheevii subsp. araraticumcum aestivum*	10
371	2.85	2.95	3.44	M8B1Z5	Lactoylglutathione lyase	*O. sativa subsp. japonica*	1
372	2.84	2.91	2.18	Q6Z8I7	Os02g0752200 protein	*O. sativa subsp. japonica*	2
373	2.83	33.18	26.58	B2LWZ7	Low molecular weight glutenin subunit I2	*Aegilops comosa*	50
374	2.83	2.90	14.17	W5I301	Uncharacterized protein	*T. timopheevii subsp. araraticumcum aestivum*	2
375	2.81	14.24	43.96	B6SYN0	Putative uncharacterized protein	*Zea mays*	14
376	2.77	33.19	34.47	A5HMG1	HMW glutenin subunit 1Bx13	*T. timopheevii subsp. araraticumcum aestivum*	63
377	2.76	11.49	38.86	P0CZ08	Avenin-like a3	*T. timopheevii subsp. araraticumcum aestivum*	21
378	2.75	11.48	49.30	C3VWV8	Dimeric alpha-amylase inhibitor	*Secale cereale*	22
379	2.72	2.79	13.33	W5ACP2	Uncharacterized protein	*T. timopheevii subsp. araraticumcum aestivum*	2
380	2.69	2.90	21.79	M7ZZ14	Defensin SD2	*T. timopheevii subsp. araraticumcum urartu*	3
381	2.66	2.72	8.14	W5E549	Uncharacterized protein	*T. timopheevii subsp. araraticumcum aestivum*	1
382	2.66	2.72	4.03	M8A9J7	Catalase	*T. timopheevii subsp. araraticumcum urartu*	2
383	2.65	2.82	4.67	W5I9I6	Uncharacterized protein	*T. timopheevii subsp. araraticumcum aestivum*	2
384	2.61	2.72	13.33	Q6Z0I0	Os08g0327700 protein	*O. sativa subsp. japonica*	2
385	2.58	40.14	42.02	A1YQH5	Glutelin	*O. sativa subsp. japonica*	67
386	2.57	22.29	27.85	A0A0K2QJ82	Alpha/beta-gliadin	*T. timopheevii subsp. araraticumcum aestivum*	24
387	2.56	2.59	7.51	W5G1S5	Uncharacterized protein	*T. timopheevii subsp. araraticumcum aestivum*	1

388	2.54	7.34	6.25	A0A0E0JM18	Uncharacterized protein	*O. punctata*	4
389	2.54	4.12	18.23	L0L5H3	Gliadin-like avenin	*A. sativa*	6
390	2.54	2.57	27.72	A0A1D6BFJ3	Uncharacterized protein	*T. timopheevii subsp. araraticumcum aestivum*	2
391	2.53	2.57	9.90	W5GGU0	Uncharacterized protein	*T. timopheevii subsp. araraticumcum aestivum*	1
392	2.52	2.56	4.60	W5ECL2	Uncharacterized protein	*T. timopheevii subsp. araraticumcum aestivum*	1
393	2.50	18.19	24.47	I0IT62	Alpha/beta-gliadin	*T. timopheevii subsp. araraticumcum aestivum*	25
394	2.48	4.61	16.40	I1GPB7	Uncharacterized protein	*Brachypodium distachyon*	4
395	2.47	2.51	12.80	A0A1D5RU62	Uncharacterized protein	*T. timopheevii subsp. araraticumcum aestivum*	2
396	2.47	2.51	1.81	A0A1D6SDX2	Uncharacterized protein	*T. timopheevii subsp. araraticumcum aestivum*	2
397	2.46	2.49	12.99	R7W7L2	Uncharacterized protein	*O. sativa subsp. japonica*	1
398	2.46	2.49	29.70	Q6ZHP6	Os02g0715400 protein	*O. sativa subsp. japonica*	2
399	2.43	6.72	18.03	B8XU25	Alfa gliadin	*T. timopheevii subsp. araraticumcum monococcum*	7
400	2.43	2.46	4.45	M8AUC3	Uncharacterized protein	*O. sativa subsp. japonica*	2
401	2.41	2.51	7.53	Q65XV6	Os05g0111200 protein	*O. sativa subsp. japonica*	1
402	2.40	2.43	3.13	M8A6E3	Bowman-Birk type trypsin inhibitor	*T. timopheevii subsp. araraticumcum urartu*	1
403	2.39	2.42	10.87	W5EM63	Uncharacterized protein	*T. timopheevii subsp. araraticumcum aestivum*	1
404	2.38	2.41	26.88	M8ATI6	Uncharacterized protein	*O. sativa subsp. japonica*	2
405	2.35	2.90	33.33	Q0DJ38	Os05g0331800 protein	*O. sativa subsp. japonica*	13
406	2.35	2.38	2.82	A0A1D5XY09	Uncharacterized protein	*T. timopheevii subsp. araraticumcum aestivum*	3
407	2.32	17.86	27.84	Q5MFP0	Low molecular weight glutenin	*T. timopheevii subsp. araraticumcum aestivum*	32
408	2.32	10.35	9.17	R9W924	ER molecular chaperone	*T. timopheevii subsp. araraticumcum aestivum*	5
409	2.31	33.26	39.86	E0Z2G5	Alpha-gliadin storage protein	*O. sativa subsp. japonica*	43

410	2.31	2.34	1.96	Q40025	Beta-glucosidase	*H. vulgare*	1
411	2.30	9.48	15.96	W5H4V7	Phosphoglycerate kinase	*T. timopheevii subsp. araraticumcum aestivum*	5
412	2.27	2.36	5.53	K3XJ87	Peroxidase	*Setaria italica*	2
413	2.27	2.29	22.69	M0UY53	Uncharacterized protein	*H. vulgare subsp. vulgare*	2
414	2.26	16.45	15.16	B5TM09	High molecular weight glutenin subunit 1Ay/Ta-e3	*T. timopheevii subsp. araraticumcum monococcum*	31
415	2.26	2.37	5.04	W5DYH1	Uncharacterized protein	*T. timopheevii subsp. araraticumcum aestivum*	1
416	2.26	2.29	5.51	W5C8D1	Uncharacterized protein	*T. timopheevii subsp. araraticumcum aestivum*	1
417	2.25	2.28	3.08	C0PDX9	Dirigent protein	*Zea mays*	1
418	2.24	2.26	20.27	W5HIB6	Defensin	*T. timopheevii subsp. araraticumcum aestivum*	1
419	2.22	2.32	3.98	B6UHQ3	Plasma membrane associated protein	*Zea mays*	1
420	2.20	2.22	5.56	W5CY88	Uncharacterized protein	*T. timopheevii subsp. araraticumcum aestivum*	1
421	2.18	2.28	1.24	W5FF37	Uncharacterized protein	*T. timopheevii subsp. araraticumcum aestivum*	1
422	2.14	4.19	18.31	R4I508	Vromindoline 3.2	*A. sativa*	4
423	2.13	16.05	48.24	L0L8B6	Gliadin-like avenin	*A. sativa*	19
424	2.13	9.91	32.12	Q7XZB4	High-molecular-weight glutenin subunit	*T. timopheevii subsp. araraticumcum aestivum*	13
425	2.13	2.14	3.63	R7WFJ2	Uncharacterized protein	*O. sativa subsp. japonica*	1
426	2.11	15.02	10.40	J9UF96	High molecular weight glutenin subunit	*T. timopheevii subsp. araraticumcum spelta*	30
427	2.11	8.21	45.74	N1QPE2	Uncharacterized protein	*O. sativa subsp. japonica*	6
428	2.10	2.11	2.15	W5CVJ9	Uncharacterized protein	*T. timopheevii subsp. araraticumcum aestivum*	1
429	2.09	5.03	29.41	W5A4Z8	Uncharacterized protein	*T. timopheevii subsp. araraticumcum aestivum*	3
430	2.09	2.19	27.37	B6UI15	Subtilisin-chymotrypsin inhibitor CI-1B	*Zea mays*	2
431	2.08	4.11	20.93	W5AQ87	Uncharacterized protein	*T. timopheevii subsp. araraticumcum aestivum*	2

432	2.08	2.10	4.10	Q60EJ0	Os05g0501700 protein	*O. sativa subsp. japonica*	1
433	2.07	4.09	3.66	Q10QG3	Cupin family protein, expressed	*O. sativa subsp. japonica*	2
434	2.06	24.31	26.10	P16315	Glutenin, low molecular weight subunit PTDUCD1	*T. timopheevii subsp. araraticumcum aestivum*	25
435	2.06	5.34	7.95	J7H390	Glucose-1-phosphate adenylyltransferase	*Zea mays*	3
436	2.05	10.36	51.42	Q2EPY2	Avenin	*A. sativa*	16
437	2.05	2.06	6.02	M8CTQ5	Dehydrin DHN1	*O. sativa subsp. japonica*	1
438	2.05	2.06	5.02	M8B9E6	Aspartate-semialdehyde dehydrogenase	*O. sativa subsp. japonica*	1
439	2.05	2.05	66.67	Q9S8W4	Alpha-amylase inhibitor	*A. sativa*	2
440	2.04	20.37	19.37	D0EVP4	LMW-m glutenin subunit	*T. timopheevii subsp. araraticumcum aestivum*	26
441	2.04	2.05	8.85	W5AP46	Uncharacterized protein	*T. timopheevii subsp. araraticumcum aestivum*	1
442	2.04	2.05	14.63	A6XER2	Defensin	*Zea mays*	1
443	2.04	2.04	5.12	Q94JF2	Os01g0705200 protein	*O. sativa subsp. japonica*	1
444	2.03	25.28	37.61	Q8W3X5	Low-molecular-weight glutenin subunit group 2 type I	*T. timopheevii subsp. araraticumcum aestivum*	44
445	2.03	4.11	13.21	Q40867	Heat shock protein 17.9	*Pennisetum americanum*	2
446	2.03	2.04	2.79	W5FSD0	Uncharacterized protein	*T. timopheevii subsp. araraticumcum aestivum*	1
447	2.03	2.04	9.09	Q8H4M5	Os07g0213600 protein	*O. sativa subsp. japonica*	1
448	2.03	2.03	7.23	Q2TN84	USP family protein	*T. timopheevii subsp. araraticumcum aestivum*	1
449	2.02	22.51	41.43	W8PUU6	Farinin protein	*Brachypodium distachyon*	46
450	2.02	11.38	29.32	A0A1D5ZYI0	Uncharacterized protein	*T. timopheevii subsp. araraticumcum aestivum*	6
451	2.02	9.29	46.00	A0A1D6BIB2	Uncharacterized protein	*T. timopheevii subsp. araraticumcum aestivum*	6
452	2.02	7.13	11.33	Q3YAF9	B hordein	*H. vulgare subsp. vulgare*	5
453	2.02	2.44	3.51	A3BQD6	Uncharacterized protein	*O. sativa subsp. japonica*	1

454	2.02	2.03	3.90	M8BTX4	12-oxophytodienoate reductase 2	*O. sativa subsp. japonica*	1
455	2.02	2.03	4.89	A0A1D5X5E7	Uncharacterized protein	*T. timopheevii subsp. araraticumcum aestivum*	1
456	2.02	2.02	15.73	B6T2T4	Nonspecific lipid-transfer protein	*Zea mays*	1
457	2.02	2.02	7.90	W5B1E5	Superoxide dismutase	*T. timopheevii subsp. araraticumcum aestivum*	1
458	2.02	2.02	3.14	Q60EH3	Os05g0449600 protein	*O. sativa subsp. japonica*	1
459	2.02	2.02	2.99	A0A1D5TJ06	Uncharacterized protein	*T. timopheevii subsp. araraticumcum aestivum*	1
460	2.01	19.98	55.63	C4P617	Monomeric alpha-amylase inhibitor	*T. timopheevii subsp. araraticumcum dicoccoides*	17
461	2.01	7.21	5.10	B6U0S1	Elongation factor 2	*Zea mays*	4
462	2.01	6.94	63.40	Q5W740	Os05g0332000 protein	*O. sativa subsp. japonica*	21
463	2.01	2.02	6.58	S4TKI5	Superoxide dismutase	*T. timopheevii subsp. araraticumcum aestivum*	1
464	2.01	2.02	8.85	Q65WT5	Os05g0445500 protein	*O. sativa subsp. japonica*	1
465	2.01	2.02	8.94	A0A1D5UXT7	Cysteine proteinase inhibitor	*T. timopheevii subsp. araraticumcum aestivum*	1
466	2.01	2.01	2.52	Q8H916	Uncharacterized protein	*O. sativa subsp. japonica*	1
467	2.01	2.01	13.92	W5G4V0	Uncharacterized protein	*T. timopheevii subsp. araraticumcum aestivum*	1
468	2.01	2.01	7.81	W5DXN2	Uncharacterized protein	*T. timopheevii subsp. araraticumcum aestivum*	1
469	2.01	2.01	2.73	Q5QPY3	Glycosyltransferase	*T. timopheevii subsp. araraticumcum aestivum*	1
470	2.00	60.19	39.65	Q68AN2	LMW-s KS2	*T. timopheevii subsp. araraticumcum aestivum*	109
471	2.00	60.19	40.77	A7XDG0	Low-molecular-weight glutenin GLU-B3	*T. timopheevii subsp. araraticumcum turgidum*	104
472	2.00	59.80	40.66	K7XEB9	Low molecular weight glutenin subunit	*O. sativa subsp. japonica*	97
473	2.00	52.77	45.69	Q0Q5D8	High-molecular-weight glutenin By8	*T. timopheevii subsp. araraticumcum aestivum*	104
474	2.00	43.46	49.90	Q0E2D2	Glutelin	*O. sativa subsp. japonica*	78

475	2.00	37.82	32.79	A5JTR3	Alpha-gliadin Gli-Ts4	*T. timopheevii subsp. araraticumcum spelta*	42
476	2.00	37.27	24.92	A5JJ46	LMW-glutenin LMW-m1	*T. timopheevii subsp. araraticumcum spelta*	58
477	2.00	31.68	36.22	A0A0E0JY02	Uncharacterized protein	*O. punctata*	40
478	2.00	28.99	25.64	A0A1G4P206	HMW glutenin i-type subunit 3A	*T. timopheevii subsp. araraticumcum aestivum*	31
479	2.00	28.53	39.84	Q08837	T. timopheevii subsp. araraticumcin	*T. timopheevii subsp. araraticumcum aestivum*	25
480	2.00	26.12	38.73	Q6PMI9	HMW glutenin subunit Dtx1.5	*O. sativa subsp. japonica*	63
481	2.00	25.40	22.92	A0A089XB95	Low-molecular-weight glutenin subunit	*T. timopheevii subsp. araraticumcum urartu*	40
482	2.00	21.83	9.60	A0A0E0CIK2	Uncharacterized protein	*O. meridionalis*	22
483	2.00	21.58	8.99	M8BLL3	Pyruvate, phosphate dikinase 1, chloroplastic	*O. sativa subsp. japonica*	11
484	2.00	21.53	64.24	C4P5N2	Monomeric alpha-amylase inhibitor	*T. timopheevii subsp. araraticumcum dicoccoides*	22
485	2.00	21.11	46.67	Q8S4P7	Thaumatin-like protein	*T. timopheevii subsp. araraticumcum aestivum*	12
486	2.00	20.74	19.17	A0A1D6APZ0	Uncharacterized protein	*T. timopheevii subsp. araraticumcum aestivum*	10
487	2.00	20.04	18.66	B0FTK5	Low molecular weight glutenin subunit	*T. timopheevii subsp. araraticumcum spelta*	27
488	2.00	19.08	64.05	A0A1B2LQE9	A. alpha amylase trypsin inhibitor	*T. timopheevii subsp. araraticumcum spelta*	11
489	2.00	19.06	39.76	R9XT02	Gamma-gliadin	*T. timopheevii subsp. araraticumcum aestivum*	40
490	2.00	19.06	25.23	Q41877	Zein protein	*Zea mays*	14
491	2.00	18.69	58.67	A0A1B2LQE6	A. alpha amylase trypsin inhibitor	*T. timopheevii subsp. araraticumcum spelta*	13
492	2.00	18.38	41.39	W8E1A7	Gamma-gliadin	*O. sativa subsp. japonica*	29
493	2.00	18.11	16.73	D7RT26	HMW glutenin subunit Gx	*T. timopheevii subsp. araraticum*	48
494	2.00	15.62	36.36	B6DQD1	Gamma-gliadin	*T. timopheevii subsp. araraticumcum spelta*	20
495	2.00	15.36	49.65	A0A173DQZ4	Type-b avenin-like protein	*T. timopheevii subsp. araraticumcum aestivum*	32
496	2.00	15.31	23.15	K7X0W1	Low molecular weight glutenin subunit	*T. timopheevii subsp. araraticumcum aestivum*	24

497	2.00	15.07	14.20	D5LMF2	Low molecular weight glutenin subunit i3	*Aegilops uniaristata*	10
498	2.00	14.05	59.15	M1MQ51	Beta-amylase	*T. timopheevii subsp. araraticumcum aestivum*	13
499	2.00	12.83	65.00	Q2XX24	Non-specific lipid-transfer protein	*Zea mays subsp. parviglumis*	9
500	2.00	12.36	9.50	E7DVE3	High molecular weight glutenin subunit Ux2.3	*T. timopheevii subsp. araraticumcum spelta*	38
501	2.00	12.13	32.16	M0Y227	Uncharacterized protein	*H. vulgare subsp. vulgare*	21
502	2.00	11.25	28.88	A0A1D6D697	Uncharacterized protein	*T. timopheevii subsp. araraticumcum aestivum*	7
503	2.00	11.20	35.00	P16347	Endogenous alpha-amylase/subtilisin inhibitor	*T. timopheevii subsp. araraticumcum aestivum*	8
504	2.00	9.87	10.44	Q3SAE3	Glucose-1-phosphate adenylyltransferase	*Zea mays*	5
505	2.00	9.85	63.51	A0A1B2LQC9	A. alpha amylase trypsin inhibitor-2	*A. sativa*	6
506	2.00	9.78	6.10	Q0D9D0	Os06g0726400 protein	*O. sativa subsp. japonica*	5
507	2.00	9.78	15.25	W5FPN2	Uncharacterized protein	*T. timopheevii subsp. araraticumcum aestivum*	5
508	2.00	9.47	43.70	Q2XX37	Non-specific lipid-transfer protein	*Zea mays subsp. parviglumis*	6
509	2.00	9.29	18.41	A0A1D5W9E0	Uncharacterized protein	*T. timopheevii subsp. araraticumcum aestivum*	5
510	2.00	8.85	34.16	A0A1D6AHC7	Uncharacterized protein	*T. timopheevii subsp. araraticumcum aestivum*	5
511	2.00	8.84	28.34	A0A1D5XQU6	Oleosin	*T. timopheevii subsp. araraticumcum aestivum*	16
512	2.00	8.23	22.34	B6UH16	Globulin	*Zea mays*	4
513	2.00	8.00	28.77	M8A1S2	Trypsin/alpha-amylase inhibitor CMX1/CMX3	*T. timopheevii subsp. araraticumcum urartu*	6
514	2.00	7.79	11.17	E9K174	Actin	*T. timopheevii subsp. araraticumcum spelta*	4
515	2.00	7.66	3.87	A0A1E5VF27	Uncharacterized protein	*T. timopheevii subsp. araraticumcum spelta*	4
516	2.00	7.48	11.98	K3XJU1	Uncharacterized protein	*Setaria italica*	4
517	2.00	7.44	36.90	A3KLI0	RAB17 protein	*Zea mays*	6
518	2.00	6.56	53.85	Q53MW2	Non-specific lipid-transfer protein	*O. sativa subsp. japonica*	7

519	2.00	6.01	21.01	T1WIP7	Dimeric alpha-amylase inhibitor	*T. timopheevii subsp. araraticumcum spelta*	4
520	2.00	5.34	4.24	M7ZLY3	Enolase 2	*T. timopheevii subsp. araraticumcum urartu*	3
521	2.00	5.14	23.33	A0A1D5SUY6	Uncharacterized protein	*T. timopheevii subsp. araraticumcum aestivum*	3
522	2.00	5.02	15.92	G8CLS2	Grain softness protein	*Taeniatherum caput-medusae*	6
523	2.00	4.54	13.17	A0A1D5SCU2	Uncharacterized protein	*T. timopheevii subsp. araraticumcum aestivum*	2
524	2.00	4.21	9.26	A0A0Q3KAB9	Uncharacterized protein	*Brachypodium distachyon*	5
525	2.00	4.10	12.99	A0A1D6AS82	Uncharacterized protein	*T. timopheevii subsp. araraticumcum aestivum*	2
526	2.00	4.07	14.19	W5DWQ4	Uncharacterized protein	*T. timopheevii subsp. araraticumcum aestivum*	2
527	2.00	4.07	14.19	M8AY04	Uncharacterized protein	*O. sativa subsp. japonica*	2
528	2.00	4.01	12.56	Q00318	Gamma-coixin 22kDa	*Coix lacryma-jobi*	4
529	2.00	4.00	13.14	Q43245	Oleosin	*Zea mays*	2
530	2.00	4.00	13.66	I1P9U0	Uncharacterized protein	*O. glaberrima*	2
531	2.00	4.00	23.89	M8C6U2	Glutaredoxin-C6	*O. sativa subsp. japonica*	2
532	2.00	4.00	16.13	B6SIZ2	Oleosin	*Zea mays*	2
533	2.00	2.58	7.43	S4U0K5	Oleosin	*O. sativa subsp. japonica*	2
534	2.00	2.58	6.01	B6UGS3	Oleosin	*Zea mays*	1
535	2.00	2.44	16.67	Q9SQG8	Pathogenesis-related protein 4	*T. timopheevii subsp. araraticumcum aestivum*	2
536	2.00	2.19	2.06	B4F832	Adenine nucleotide transporter BT1 chloroplastic/mitochondrial	*Zea mays*	1
537	2.00	2.15	16.16	I1NMC9	Uncharacterized protein	*O. glaberrima*	1
538	2.00	2.10	6.47	A0A1D5UHV8	Uncharacterized protein	*T. timopheevii subsp. araraticumcum aestivum*	1
539	2.00	2.08	3.57	A0A1R3PYI9	Oil body-associated protein 2A	*Zea mays*	1
540	2.00	2.06	8.54	Q5URW0	Grain softness protein	*H. vulgare subsp. vulgare*	3

541	2.00	2.02	1.87	B9EX51	Uncharacterized protein	*O. sativa subsp. japonica*	1
542	2.00	2.01	9.72	O82688	Amino acid selective channel protein	*H. vulgare*	1
543	2.00	2.01	6.59	A0A1D5VFL6	Uncharacterized protein	*T. timopheevii subsp. araraticumcum aestivum*	1
544	2.00	2.00	1.85	F6MIX2	Purple acid phosphatase	*Secale cereale*	1
545	2.00	2.00	4.04	W5GUD5	Uncharacterized protein	*T. timopheevii subsp. araraticumcum aestivum*	1
546	2.00	2.00	16.67	W5FQG6	40S ribosomal protein S26	*T. timopheevii subsp. araraticumcum aestivum*	3
547	2.00	2.00	4.41	W5ETF4	Uncharacterized protein	*T. timopheevii subsp. araraticumcum aestivum*	1
548	2.00	2.00	9.02	Q42848	Non-specific lipid-transfer protein	*H. vulgare*	1
549	2.00	2.00	3.45	M7YT47	Zinc finger CCCH domain-containing protein 12	*T. timopheevii subsp. araraticumcum urartu*	1
550	2.00	2.00	6.41	K7VEA3	Embryonic cell protein 63	*Zea mays*	1
551	2.00	2.00	6.92	F4ZL26	Alpha-gliadin storage protein	*Secale strictum*	1
552	2.00	2.00	0.58	A0A1D6F400	CW-type Zinc Finger	*Zea mays*	1
553	2.00	2.00	3.18	A0A1D5Y1A6	Uncharacterized protein	*T. timopheevii subsp. araraticumcum aestivum*	2
554	2.00	2.00	11.65	A0A1D5XKT5	Uncharacterized protein	*T. timopheevii subsp. araraticumcum aestivum*	1
555	2.00	2.00	2.07	A0A0D9X7Z0	Uncharacterized protein	*O. meridionalis*	1
556	2.00	2.00	2.08	W8SJN1	6-phosphogluconate dehydrogenase, decarboxylating	*T. timopheevii subsp. araraticumcum spelta*	1
557	2.00	2.00	10.43	W5FIX9	Uncharacterized protein	*T. timopheevii subsp. araraticumcum aestivum*	1
558	2.00	2.00	6.94	W5FEZ3	40S ribosomal protein S12	*T. timopheevii subsp. araraticumcum aestivum*	1
559	2.00	2.00	7.69	W5F7Q9	Uncharacterized protein	*T. timopheevii subsp. araraticumcum aestivum*	1
560	2.00	2.00	1.60	W5ER56	Uncharacterized protein	*T. timopheevii subsp. araraticumcum aestivum*	1
561	2.00	2.00	3.70	W5CQ97	Cysteine proteinase inhibitor	*T. timopheevii subsp. araraticumcum aestivum*	1
562	2.00	2.00	2.52	W5C0E2	Uncharacterized protein	*T. timopheevii subsp. araraticumcum aestivum*	1

563	2.00	2.00	3.53	W5BGU3	Uncharacterized protein	*T. timopheevii subsp. araraticumcum aestivum*	1
564	2.00	2.00	8.18	W5BFB7	Uncharacterized protein	*T. timopheevii subsp. araraticumcum aestivum*	1
565	2.00	2.00	5.56	W5AR77	Uncharacterized protein	*T. timopheevii subsp. araraticumcum aestivum*	1
566	2.00	2.00	15.91	W4ZN48	Uncharacterized protein	*T. timopheevii subsp. araraticumcum aestivum*	1
567	2.00	2.00	1.64	T1L810	Uncharacterized protein	*T. timopheevii subsp. araraticumcum urartu*	1
568	2.00	2.00	4.22	R7W7W8	Uncharacterized protein	*O. sativa subsp. japonica*	1
569	2.00	2.00	2.90	R7W5Z8	Adenylate kinase A	*O. sativa subsp. japonica*	1
570	2.00	2.00	7.06	R7W056	Uncharacterized protein	*O. sativa subsp. japonica*	1
571	2.00	2.00	1.31	Q9ZT12	101 kDa heat shock protein	*T. timopheevii subsp. araraticumcum aestivum*	1
572	2.00	2.00	5.59	Q9AUN6	Putative uncharacterized protein	*O. sativa subsp. japonica*	1
573	2.00	2.00	4.11	Q6YY64	60S ribosomal protein L6	*O. sativa subsp. japonica*	1
574	2.00	2.00	4.65	Q2QN90	Bromodomain associated family protein, expressed	*O. sativa subsp. japonica*	1
575	2.00	2.00	1.38	M8C9M7	Uncharacterized protein	*O. sativa subsp. japonica*	1
576	2.00	2.00	4.67	M8BWJ2	Uncharacterized protein	*O. sativa subsp. japonica*	1
577	2.00	2.00	1.48	M8BVK5	Uncharacterized protein	*O. sativa subsp. japonica*	1
578	2.00	2.00	8.39	M8AW09	40S ribosomal protein S17-4	*T. timopheevii subsp. araraticumcum urartu*	1
579	2.00	2.00	5.77	M0XN12	Uncharacterized protein	*H. vulgare subsp. vulgare*	1
580	2.00	2.00	6.02	K4AE73	Uncharacterized protein	*Setaria italica*	2
581	2.00	2.00	0.71	K3Z3B0	Uncharacterized protein	*Setaria italica*	1
582	2.00	2.00	2.35	J3LAZ4	Uncharacterized protein	*O. brachyantha*	1
583	2.00	2.00	8.21	J3L3I8	Uncharacterized protein	*O. brachyantha*	1
584	2.00	2.00	2.21	I1IGR0	Uncharacterized protein	*Brachypodium distachyon*	1

585	2.00	2.00	4.37	I1GR67	Peroxidase	*Brachypodium distachyon*	1
586	2.00	2.00	5.34	G9DRA0	MYB-related protein	*T. timopheevii subsp. araraticumcum aestivum*	1
587	2.00	2.00	7.91	F2D3I1	Predicted protein	*H. vulgare subsp. vulgare*	1
588	2.00	2.00	6.63	C0PH28	40S ribosomal protein S10-1	*Zea mays*	1
589	2.00	2.00	5.67	B6UHT3	Uncharacterized protein	*Zea mays*	1
590	2.00	2.00	2.36	A0A1E5VP17	Uncharacterized protein	*T. timopheevii subsp. araraticumcum spelta*	1
591	2.00	2.00	15.19	A0A1D6S463	Uncharacterized protein	*T. timopheevii subsp. araraticumcum aestivum*	1
592	2.00	2.00	5.49	A0A1D5YH09	Uncharacterized protein	*T. timopheevii subsp. araraticumcum aestivum*	1
593	2.00	2.00	11.43	A0A1D5SGD9	Uncharacterized protein	*T. timopheevii subsp. araraticumcum aestivum*	1
594	2.00	2.00	2.37	A0A0E0LQ31	Uncharacterized protein	*O. punctata*	1
595	2.00	2.00	7.09	A0A0A9U730	Uncharacterized protein	*Arundo donax*	1
596	2.00	2.00	2.34	A0A0A9PKF2	Uncharacterized protein	*Arundo donax*	1
597	2.00	2.00	17.65	A0A0A9AM13	Atp1, OrsajM_p50	*Arundo donax*	1
598	2.00	2.00	3.25	A0A077RY90	Uncharacterized protein	*T. timopheevii subsp. araraticumcum aestivum*	1
599	1.96	2.00	3.40	Q6L5M3	40S ribosomal protein S4	*Bromus inermis*	1
600	1.94	2.02	3.02	W5B7D0	Uncharacterized protein	*T. timopheevii subsp. araraticumcum aestivum*	1
601	1.93	2.09	1.01	W5EB84	Uncharacterized protein	*T. timopheevii subsp. araraticumcum aestivum*	1
602	1.92	17.32	26.33	Q2QL55	Alpha-gliadin storage protein	*Aegilops speltoides*	24
603	1.92	2.00	5.61	A0A1D5S2X8	Uncharacterized protein	*T. timopheevii subsp. araraticumcum aestivum*	1
604	1.89	2.00	0.98	Q65XQ8	Os05g0151400 protein	*O. sativa subsp. japonica*	1
605	1.85	17.16	7.30	A0A0D9VE98	Uncharacterized protein	*O. meridionalis*	19
606	1.85	4.00	20.37	Q2QLR2	Glycine-rich RNA-binding protein GRP1A, putative, expressed	*O. sativa subsp. japonica*	3

607	1.83	2.00	5.53	Q76ME3	ADP-ribosylation factor	*T. timopheevii subsp. araraticumcum aestivum*	1
608	1.82	1.96	7.34	A9XEB9	50kD gamma canein	*Saccharum officinarum*	1
609	1.82	1.96	1.96	W5FY62	Glucose-6-phosphate isomerase	*T. timopheevii subsp. araraticumcum aestivum*	1
610	1.80	1.92	1.18	R7W244	E3 SUMO-protein ligase SIZ1	*O. sativa subsp. japonica*	1
611	1.77	12.42	10.98	A0A1D5XVS8	Uncharacterized protein	*T. timopheevii subsp. araraticumcum aestivum*	8
612	1.77	1.89	1.08	A0A0E0J0G8	Uncharacterized protein	*O. nivara*	1
613	1.77	1.89	1.42	A0A0E0K139	Uncharacterized protein	*O. punctata*	1
614	1.74	13.38	27.44	W5AUU7	Uncharacterized protein	*T. timopheevii subsp. araraticumcum aestivum*	9
615	1.74	1.89	4.46	A0A0E0F914	Uncharacterized protein	*O. meridionalis*	1
616	1.74	1.85	1.24	J3LME7	Uncharacterized protein	*O. brachyantha*	1
617	1.73	1.87	1.88	Q6ZK46	Os08g0127900 protein	*O. sativa subsp. japonica*	1
618	1.72	1.90	2.60	A0A1D6BGP6	Uncharacterized protein	*T. timopheevii subsp. araraticumcum aestivum*	1
619	1.72	1.85	1.84	Q10JB2	Chaperone clpB 1, putative, expressed	*O. sativa subsp. japonica*	1
620	1.66	6.26	3.77	Q93WS3	Sucrose synthase	*Zea mays*	5
621	1.64	1.77	2.98	W5G105	Uncharacterized protein	*T. timopheevii subsp. araraticumcum aestivum*	1
622	1.60	1.74	7.75	Q4FZ49	Cysteine proteinase inhibitor	*Zea mays*	1
623	1.59	42.55	43.05	F1CYR8	High-molecular-weight glutenin subunit	*T. timopheevii subsp. araraticumcum spelta*	113
624	1.57	1.80	8.43	A0A1D5ZZJ8	Uncharacterized protein	*T. timopheevii subsp. araraticumcum aestivum*	2
625	1.56	1.71	5.57	W5GF29	Peroxidase	*T. timopheevii subsp. araraticumcum aestivum*	1
626	1.55	12.08	9.23	C9E6N3	High molecular weight glutenin y-type subunit Hy3	*T. timopheevii subsp. araraticumcum spelta*	15
627	1.55	1.85	4.35	Q8H8B0	Os03g0159600 protein	*O. sativa subsp. japonica*	1
628	1.55	1.67	7.69	C5YYN1	Uncharacterized protein	*Sorghum bicolor*	1

629	1.54	1.68	4.02	I1QCX5	Uncharacterized protein	*O. glaberrima*	1
630	1.54	1.66	1.89	I1I6Q4	REVERSED Uncharacterized protein	*Brachypodium distachyon*	1
631	1.53	2.88	5.20	R4ZCU4	L-1	*T. timopheevii subsp. araraticum*	2
632	1.52	1.87	2.12	C5Z7L1	Pyrophosphate--fructose 6-phosphate 1-phosphotransferase subunit beta	*Sorghum bicolor*	1
633	1.52	1.75	7.92	W5CWR9	Uncharacterized protein	*T. timopheevii subsp. araraticumcum aestivum*	2
634	1.51	1.72	6.34	C5XFV5	Uncharacterized protein	*Sorghum bicolor*	1
635	1.51	1.64	6.18	Q7X9L9	QM	*T. timopheevii subsp. araraticumcum aestivum*	1
636	1.48	1.97	1.09	I1P0X2	Uncharacterized protein	*O. glaberrima*	1
637	1.47	1.59	3.12	Q6EQL0	Putative uncharacterized protein	*O. sativa subsp. japonica*	1
638	1.46	7.99	20.33	A0A1D5SF46	Uncharacterized protein	*T. timopheevii subsp. araraticumcum aestivum*	4
639	1.43	1.55	2.62	F2E9C4	Predicted protein	*H. vulgare subsp. vulgare*	1
640	1.43	1.55	2.86	Q84MJ5	Methylmalonate semialdehyde dehydrogenase	*T. timopheevii subsp. araraticumcum aestivum*	1
641	1.42	42.80	37.64	Q6UJY7	HMW-glutenin By subunit	*T. timopheevii subsp. araraticumcum turgidum*	99
642	1.42	1.55	12.00	A0A0A8YIV5	Uncharacterized protein	*Arundo donax*	1
643	1.41	1.54	3.36	Q6H7I9	ATP-dependent Clp protease proteolytic subunit	*O. sativa subsp. japonica*	1
644	1.36	3.65	21.48	H6U7Z1	PINB-2v5-2	*T. timopheevii subsp. araraticumcum aestivum*	5
645	1.35	3.57	9.93	Q6JBR2	Chitinase	*Tripsacum dactyloides*	2
646	1.32	8.28	14.76	Q40069	Peroxidase	*H. vulgare*	5
647	1.32	1.43	3.62	W5CQE4	Uncharacterized protein	*T. timopheevii subsp. araraticumcum aestivum*	1
648	1.31	1.42	8.91	R7WAY7	Carbonic anhydrase	*O. sativa subsp. japonica*	1
649	1.30	6.05	30.46	W5AGK9	Nucleoside diphosphate kinase	*T. timopheevii subsp. araraticumcum aestivum*	4

650	1.30	1.42	11.34	Q7XDY1	Expressed protein	*O. sativa subsp. japonica*	1
651	1.29	1.41	1.28	X2D2V6	Lipoxygenase	*T. timopheevii subsp. araraticumcum aestivum*	1
652	1.28	35.76	27.37	M8A4Z7	Glutenin, high molecular weight subunit DX5	*T. timopheevii subsp. araraticumcum urartu*	100
653	1.28	12.39	16.36	H6UQP6	High molecular weight subunit of glutenin	*T. timopheevii subsp. araraticumcum spelta*	30
654	1.28	5.94	13.51	A0A1E5VCS5	1-Cys peroxiredoxin PER1	*T. timopheevii subsp. araraticumcum spelta*	3
655	1.28	3.63	17.43	Q6I5U6	Os05g0432700 protein	*O. sativa subsp. japonica*	2
656	1.26	13.34	15.38	J3LBL1	Uncharacterized protein	*O. brachyantha*	13
657	1.26	1.48	18.75	T2B4Q5	Nonspecific lipid transfer protein 2	*O. sativa*	2
658	1.26	1.38	0.91	W5H4V8	Aminopeptidase	*T. timopheevii subsp. araraticumcum aestivum*	1
659	1.26	1.38	17.33	M7ZE88	Uncharacterized protein	*T. timopheevii subsp. araraticumcum urartu*	2
660	1.24	8.55	34.75	M8B2U4	Uncharacterized protein	*O. sativa subsp. japonica*	6
661	1.24	8.12	14.21	A0A1D6KL30	Sorghum bicolortol dehydrogenase	*Zea mays*	6
662	1.24	1.41	1.83	A0A1D5YN92	Uncharacterized protein	*T. timopheevii subsp. araraticumcum aestivum*	2
663	1.22	1.34	10.23	W5EJM2	Uncharacterized protein	*T. timopheevii subsp. araraticumcum aestivum*	1
664	1.21	45.23	35.06	X2JBS3	Low-molecular-weight glutenin subunit Glu-D3	*T. timopheevii subsp. araraticumcum aestivum*	65
665	1.21	1.35	2.39	M7ZMS4	Endoglucanase	*T. timopheevii subsp. araraticumcum urartu*	1
666	1.20	1.32	2.97	K3ZHS3	Uncharacterized protein	*Setaria italica*	1
667	1.19	1.31	10.13	A0A1D6A246	Uncharacterized protein	*T. timopheevii subsp. araraticumcum aestivum*	1
668	1.18	6.31	5.60	I1IMR6	Protein disulfide-isomerase	*Brachypodium distachyon*	3
669	1.18	1.31	5.42	M8B3B6	Uncharacterized protein	*O. sativa subsp. japonica*	1
670	1.18	1.30	4.42	U3N1P5	Glutamine synthetase	*T. timopheevii subsp. araraticumcum turgidum*	1
671	1.17	35.61	43.49	A0A0E3Z6T7	Alpha-gliadin	*T. timopheevii subsp. araraticumcum spelta*	39

672	1.15	1.27	1.38	W5FSK5	Polyadenylate-binding protein	*T. timopheevii subsp. araraticumcum aestivum*	1
673	1.14	17.48	39.78	J7HYC2	Gamma-gliadin	*Aegilops speltoides*	26
674	1.13	7.63	33.57	A0A1B2LQB6	A. alpha amylase trypsin inhibitor	*T. timopheevii subsp. araraticumcum spelta*	5
675	1.12	1.24	11.49	W5GVJ0	Uncharacterized protein	*T. timopheevii subsp. araraticumcum aestivum*	1
676	1.11	1.23	2.66	A0A1E5WB11	Uncharacterized protein	*T. timopheevii subsp. araraticumcum spelta*	1
677	1.11	1.23	9.84	A0A0A9KM48	Prpl35-1	*Arundo donax*	1
678	1.09	20.07	42.75	J7HUW3	Gamma-gliadin	*Aegilops speltoides*	28
679	1.08	13.74	13.20	Q93XQ7	Protein disulfide-isomerase	*T. timopheevii subsp. araraticumcum aestivum*	7
680	1.08	1.19	2.35	B9G865	Uncharacterized protein	*O. sativa subsp. japonica*	1
681	1.07	19.56	26.02	M7Z1Z4	Serpin-Z2B	*T. timopheevii subsp. araraticumcum urartu*	12
682	1.07	1.18	6.10	A0A0D9V107	Uncharacterized protein	*O. meridionalis*	1
683	1.06	1.18	1.91	A0A1B6P8P7	Uncharacterized protein	*Sorghum bicolor*	1
684	1.05	1.15	18.46	W5E8X2	Uncharacterized protein	*T. timopheevii subsp. araraticumcum aestivum*	1
685	1.04	1.18	2.46	A0A060N0S6	Omega-gliadin	*T. timopheevii subsp. araraticumcum aestivum*	1
686	1.04	1.16	1.30	W5GA68	Pyrophosphate--fructose 6-phosphate 1-phosphotransferase subunit alpha	*T. timopheevii subsp. araraticumcum aestivum*	1
687	1.04	1.14	10.00	Q5I7K5	Ribosomal protein P1	*T. timopheevii subsp. araraticumcum aestivum*	1
688	1.03	14.21	35.09	J7HY97	Gamma-gliadin	*Aegilops comosa*	32
689	1.03	1.14	1.96	W5EIX6	Pyruvate kinase	*T. timopheevii subsp. araraticumcum aestivum*	1
690	1.03	1.14	15.22	R7W070	Uncharacterized protein	*O. sativa subsp. japonica*	1
691	1.03	1.14	4.85	Q2XXK7	Zeamatin-like protein	*Zea mays subsp. parviglumis*	1
692	1.02	1.12	1.06	F2DLY1	Predicted protein	*H. vulgare subsp. vulgare*	1

693	1.01	17.07	21.13	Q2XQF1	B hordein	*H. vulgare subsp. vulgare*	25
694	1.01	13.97	21.10	I1HMK8	Uncharacterized protein	*Brachypodium distachyon*	27
695	1.00	1.10	4.46	A0A0E0LK26	Uncharacterized protein	*O. punctata*	1
696	1.00	1.10	6.84	A0A0A9P4I7	Uncharacterized protein	*Arundo donax*	1
697	0.98	1.10	2.53	Q6AVU2	Endoplasmic oxidoreductin 1, putative, expressed	*O. sativa subsp. japonica*	1
698	0.97	1.07	13.73	A0A0A9LSK5	Uncharacterized protein	*Arundo donax*	1
699	0.96	1.07	5.26	Q67VZ0	Os06g0221300 protein	*O. sativa subsp. japonica*	1
700	0.95	1.06	1.08	R7W333	Alpha-mannosidase	*Aegilops tauschii*	1
701	0.94	4.13	27.10	W5ANC8	Uncharacterized protein	*T. timopheevii subsp. araraticumcum aestivum*	3
702	0.94	1.04	2.25	A0A0D9XVQ3	Uncharacterized protein	*Leersia perrieri*	1
703	0.93	18.17	9.68	E9NQE5	Pyruvate orthophosphate dikinase 1	*Zea mays*	9
704	0.93	1.04	1.99	Q53M54	Expressed protein	*O. sativa subsp. japonica*	1
705	0.92	1.02	3.15	I1IMT6	Uncharacterized protein	*Brachypodium distachyon*	1
706	0.91	2.35	4.41	A0A0E0M9G6	Uncharacterized protein	*O. punctata*	2
707	0.91	1.06	5.00	M8BX24	Uncharacterized protein	*Aegilops tauschii*	1
708	0.91	1.03	3.27	M8CV71	3-ketoacyl-CoA thiolase 2, peroxisomal	*Aegilops tauschii*	1
709	0.89	1.00	2.19	Q5NA76	Putative uncharacterized protein B1066G12.18	*O. sativa subsp. japonica*	1
710	0.89	1.00	5.74	A0A1D6PTV8	Glutamyl-tRNA reductase-binding protein chloroplastic	*Zea mays*	1
711	0.88	21.36	12.88	A0A1D5U769	Sucrose synthase	*T. timopheevii subsp. araraticumcum aestivum*	12
712	0.88	1.02	3.66	A0A1D6H070	Leucine-rich repeat/extensin 2	*Zea mays*	2
713	0.86	0.96	2.82	K7VF10	Coiled-coil domain-containing protein 97 isoform 1	*Zea mays*	1
714	0.85	42.29	48.42	E0Z2G9	Alpha-gliadin storage protein	*Aegilops tauschii*	46

715	0.85	0.99	2.79	W5AMP7	Uncharacterized protein	*T. timopheevii subsp. araraticumcum aestivum*	1
716	0.84	0.95	5.81	W5A8H1	Histone H2A	*T. timopheevii subsp. araraticumcum aestivum*	1
717	0.84	0.95	3.17	I1QG82	Uncharacterized protein	*O. glaberrima*	1
718	0.83	13.68	8.52	V5RM88	D-Hordein	*Elymus sibiricus*	31
719	0.82	0.92	8.85	W4ZQ59	Uncharacterized protein	*T. timopheevii subsp. araraticumcum aestivum*	1
720	0.82	0.91	1.93	A0A1D5WYY4	Uncharacterized protein	*T. timopheevii subsp. araraticumcum aestivum*	1

Supplementary Table 2. Protein identifications in breakfast bars (BB). Trypsin digestion peptides were identified after database searching against the Poaceae subset of the Uniprot database appended with the Common Repository of Adventitious Protein (cRAP) database using a 1% global false discovery rate (FDR) threshold.

N	Unused Score	Total Score	% Cov (95%)	Accession	Name	Species	Peptides (95%)
1	53.55	53.55	41.73	B2Y2S3	Low molecular weight glutenin subunit	*T. timopheevii subsp. araraticumcum aestivum*	88
2	47.23	47.23	71.11	A0A1D5YFA7	Beta-amylase	*T. timopheevii subsp. araraticumcum aestivum*	46
3	38.00	38.00	31.90	Q41553	HMW glutenin subunit Ax2	*T. timopheevii subsp. araraticumcum aestivum*	48
4	37.75	37.79	67.67	W5FZ62	Uncharacterized protein	*T. timopheevii subsp. araraticumcum aestivum*	23
5	33.65	33.65	33.16	I6QQ39	Globulin-3A	*T. timopheevii subsp. araraticumcum aestivum*	20
6	33.16	33.17	32.45	Q38780	11S globulin	*A. sativa*	27
7	29.16	29.17	44.79	K7XE90	Alpha-gliadin	*T. timopheevii subsp. araraticumcum aestivum*	32
8	28.73	28.74	75.60	Q6S5B1	Alpha amylase inhibitor CM3	*T. timopheevii subsp. araraticumcum turgidum subsp. durum*	37
9	26.91	26.91	70.71	M8CU50	Uncharacterized protein	*Aegilops tauschii*	51
10	25.28	25.28	20.06	K2C1	Keratin	*Homo sapiens*	12
11	24.82	24.83	81.56	C3VWA4	Dimeric alpha-amylase inhibitor	*T. timopheevii subsp. araraticumcum dicoccoides*	25
12	24.54	24.72	33.61	W6AX70	High molecular weight glutenin subunit	*T. timopheevii subsp. araraticumcum aestivum*	41
13	24.39	29.58	38.08	A1YQG3	Glutelin	*O. sativa subsp. japonica*	25
14	23.67	23.74	47.13	B6UKL5	Gamma-gliadin	*T. timopheevii subsp. araraticumcum turgidum*	35
15	23.65	23.74	26.17	M7ZQM3	Globulin-1 S allele	*T. timopheevii subsp. araraticumcum urartu*	12
16	23.36	27.65	20.88	G4Y3Y0	High-molecular-weight glutenin subun	*T. timopheevii subsp. araraticumcum aestivum*	27
17	23.32	27.39	10.84	A0A0E0FMV5	Uncharacterized protein	*O. nivara*	19
18	23.29	23.57	37.09	W8E2L5	Gamma-gliadin	*Aegilops tauschii*	30
19	22.61	22.83	21.14	W5AKY9	Uncharacterized protein	*T. timopheevii subsp. araraticumcum aestivum*	10
20	21.71	22.63	70.86	X2KYP9	Monomeric alpha-amylase inhibitor	*T. timopheevii subsp. araraticumcum aestivum*	23
21	21.22	21.23	56.34	A0A173DQZ4	Type-b avenin-like protein	*T. timopheevii subsp. araraticumcum aestivum*	27
22	20.14	20.15	38.08	A0A0E0E950	Uncharacterized protein	*O. meridionalis*	51
23	19.95	20.03	73.28	Q8GZB0	Non-specific lipid-transfer protein	*T. timopheevii subsp. araraticumcum aestivum*	20

24	19.52	21.65	44.58	W5I5X1	Uncharacterized protein	*T. timopheevii subsp. araraticumcum aestivum*	15
25	18.74	18.75	61.50	Q8LKV8	Seed globulin	*Aegilops tauschii*	13
26	18.42	29.41	30.54	Q84U14	Low-molecular-weight glutenin subunit	*T. timopheevii subsp. araraticumcum aestivum*	29
27	18.30	23.10	28.28	T1T6C4	Glutelin	*O. sativa subsp. indica*	18
28	17.86	20.01	48.23	B6DQD5	Gamma-gliadin	*T. timopheevii subsp. araraticumcum aestivum*	27
29	17.23	27.35	37.19	M8C2Y1	Serpin-Z2B	*Aegilops tauschii*	14
30	17.14	17.16	60.93	A0A1B2LQE5	A. alpha amylase trypsin inhibitor	*T. timopheevii subsp. araraticumcum aestivum*	11
31	16.95	19.13	27.61	B8AH66	Putative uncharacterized protein	*O. sativa subsp. indica*	12
32	16.71	16.72	14.37	A0A0K0KDM6	High molecular weight glutenin subunit	*T. timopheevii subsp. araraticumcum aestivum*	28
33	16.45	16.46	52.38	Q2A784	Avenin-like a1	*T. timopheevii subsp. araraticumcum aestivum*	22
34	16.33	16.48	56.82	A0A0E0Q5N0	Uncharacterized protein	*O. rufipogon*	22
35	16.11	16.17	11.73	Q9SAU8	HSP70	*T. timopheevii subsp. araraticumcum aestivum*	8
36	16.09	32.47	43.05	P14812	12S seed storage globulin 2	*A. sativa*	32
37	16.03	16.07	44.00	W5FN32	Uncharacterized protein	*T. timopheevii subsp. araraticumcum aestivum*	8
38	15.85	30.09	24.24	W5EST8	Uncharacterized protein	*T. timopheevii subsp. araraticumcum aestivum*	16
39	15.57	15.60	68.82	W4ZP51	Uncharacterized protein	*T. timopheevii subsp. araraticumcum aestivum*	16
40	15.43	20.66	37.10	A0A1P8DTB4	Alpha-gliadin storage protein	*T. timopheevii subsp. araraticumcum aestivum*	31
41	15.33	15.52	10.79		K1C10_HUMAN	7	7
42	15.21	15.88	63.45	P16851	Alpha-amylase/trypsin inhibitor CM2	*T. timopheevii subsp. araraticumcum aestivum*	26
43	14.91	14.93	66.90	B9VRI3	Alpha-amylase inhibitor CM16 subunit	*T. timopheevii subsp. araraticumcum aestivum*	28
44	14.68	14.71	34.37	Q7X6I8	OJ000315_02.8 protein	*O. sativa subsp. japonica*	9
45	14.51	18.98	24.07	T1T5D8	Glutelin	*O. sativa subsp. indica*	16
46	14.35	14.41	27.09	Q8LK23	Peroxidase	*T. timopheevii subsp. araraticumcum aestivum*	8
47	14.24	14.32	35.34	I4EP67	Avenin	*T. timopheevii subsp. araraticumcum aestivum*	29
48	13.46	42.21	56.55	A0A1D5XGF4	Beta-amylase	*T. timopheevii subsp. araraticumcum aestivum*	41
49	13.37	13.47	14.82	A0A1D6ABF1	Uncharacterized protein	*T. timopheevii subsp. araraticumcum aestivum*	9
50	13.05	17.22	35.12	A0A0E0AHA2	Uncharacterized protein	*O. meridionalis*	26

51	12.71	12.72	29.36	Q9AXH7	1-Cys peroxiredoxin	*T. timopheevii subsp. araraticumcum turgidum subsp. durum*	6
52	12.52	12.56	13.22	W4ZRH9	Glucose-1-phosphate adenylyltransfera	*T. timopheevii subsp. araraticumcum aestivum*	5
53	12.48	12.50	36.11	P16347	Endogenous alpha-amylase/subtilisin in	*T. timopheevii subsp. araraticumcum aestivum*	7
54	12.27	12.35	13.80	K1C9_HUMAN			7
55	12.20	12.24	7.32	Q0D9D0	Os06g0726400 protein	*O. sativa subsp. japonica*	6
56	12.17	12.21	41.59	I7KM78	Gamma-gliadin	*T. timopheevii subsp. araraticumcum aestivum*	13
57	12.10	26.85	41.67	B2BZC7	LMW-m glutenin subunit 0154A5-M	*T. timopheevii subsp. araraticumcum aestivum*	44
58	12.07	12.10	12.62	Q93XQ7	Protein disulfide-isomerase	*T. timopheevii subsp. araraticumcum aestivum*	6
59	12.04	21.25	40.89	D2X6D9	Alpha-gliadin	*T. timopheevii subsp. araraticumcum turgidum subsp. durum*	38
60	12.00	21.20	22.97	A0A1D5ZZT8	Uncharacterized protein	*T. timopheevii subsp. araraticumcum aestivum*	11
61	12.00	12.00	37.76	A0A1B2LQC0	A. alpha amylase trypsin inhibitor	*A. sativa*	7
62	11.85	11.91	39.31	M8BAK8	Uncharacterized protein	*Aegilops tauschii*	7
63	11.80	11.96	39.60	A0A0E0HAY8	Uncharacterized protein	*O. nivara*	19
64	11.78	11.95	38.21	W5A2C0	Uncharacterized protein	*T. timopheevii subsp. araraticumcum aestivum*	9
65	11.71	21.42	19.48	A0A068F6Z7	Low molecular weight glutenin	*T. timopheevii subsp. araraticumcum dicoccoides*	39
66	11.61	11.72	14.12	M7YLN2	Aspartate aminotransferase	*T. timopheevii subsp. araraticumcum urartu*	5
67	11.59	11.72	100.00	P82901	Non-specific lipid-transfer protein 2P	*T. timopheevii subsp. araraticumcum aestivum*	13
68	11.55	11.62	33.19	W5EFT2	Uncharacterized protein	*T. timopheevii subsp. araraticumcum aestivum*	7
69	11.47	11.56	63.51	A0A1B2LQD4	A. alpha amylase trypsin inhibitor-2	*A. sativa*	7
70	11.43	11.51	39.06	I3NM41	Oleosin	*T. timopheevii subsp. araraticumcum aestivum*	11
71	11.21	11.35	21.05	R9UNY9	Xylanase inhibitor protein	*T. timopheevii subsp. araraticumcum aestivum*	6
72	11.17	11.45	57.69	Q0IP02	Os12g0269200 protein	*O. sativa subsp. japonica*	42
73	11.05	22.40	81.56	Q4U199	Dimeric alpha-amylase inhibitor	*T. timopheevii subsp. araraticumcum aestivum*	16

74	10.83	11.00	23.00	A0A0E0AHC4	Uncharacterized protein	*O. meridionalis*	9
75	10.76	10.94	47.22	W5AKQ2	Uncharacterized protein	*T. timopheevii subsp. araraticumcum aestivum*	6
76	10.75	10.91	36.14	G1JSL4	Peroxygenase 1	*A. sativa*	7
77	10.68	10.87	10.24	M8BDK9	Alanine aminotransferase 2	*Aegilops tauschii*	5
78	10.60	10.81	37.23	TRYP_PIG			43
79	10.51	10.56	41.38	A0A1D6DC72	Uncharacterized protein	*T. timopheevii subsp. araraticumcum aestivum*	17
80	10.36	10.56	7.72	W5B5R3	Sucrose synthase	*T. timopheevii subsp. araraticumcum aestivum*	5
81	10.15	13.85	66.67	Q9M4N7	Early-methionine-labelled polypeptide	*Secale cereale*	9
82	10.15	10.21	19.34	A0A1D5VIX2	Uncharacterized protein	*T. timopheevii subsp. araraticumcum aestivum*	5
83	9.97	13.80	27.44	W5AUU7	Uncharacterized protein	*T. timopheevii subsp. araraticumcum aestivum*	7
84	9.94	10.00	57.29	R7WGD7	Uncharacterized protein	*Aegilops tauschii*	5
85	9.89	10.00	30.83	A0A1D5YGW1	Uncharacterized protein	*T. timopheevii subsp. araraticumcum aestivum*	5
86	9.83	11.93	56.64	A0A1B2LQA9	A. alpha amylase trypsin inhibitor	*A. sativa*	9
87	9.68	9.76	30.40	W5DQS5	Uncharacterized protein	*T. timopheevii subsp. araraticumcum aestivum*	5
88	9.63	9.68	46.34	W5D637	Uncharacterized protein	*T. timopheevii subsp. araraticumcum aestivum*	5
89	9.57	9.63	43.03	A0A1D5RYA9	Uncharacterized protein	*T. timopheevii subsp. araraticumcum aestivum*	6
90	9.48	9.60	4.25	B9W4U7	Starch branching enzyme IIa	*T. timopheevii subsp. araraticumcum aestivum*	5
91	9.27	9.97	25.98	L0L6J0	Gliadin-like avenin	*A. sativa*	21
92	9.24	9.30	56.86	A1YQF0	Os05g0331532 protein	*O. sativa subsp. japonica*	15
93	9.21	9.34	8.71	Q6Z782	Os02g0202400 protein	*O. sativa subsp. japonica*	4
94	9.15	41.80	29.66	Q00M56	LMW-GS P-31	*T. timopheevii subsp. araraticumcum aestivum*	64
95	8.96	9.02	44.90	U6A2I2	Vromindoline VIN2	*T. timopheevii subsp. araraticumcum aestivum*	12
96	8.87	8.96	15.80	T1MSW5	Uncharacterized protein	*T. timopheevii subsp. araraticumcum urartu*	5
97	8.78	8.86	15.46	W5BPU1	40S ribosomal protein SA	*T. timopheevii subsp. araraticumcum aestivum*	4

98	8.51	8.56	19.56	A0A1D6BR50	Uncharacterized protein	*T. timopheevii subsp. araraticumcum aestivum*	5
99	8.51	8.55	25.08	A0A1D5XF71	Uncharacterized protein	*T. timopheevii subsp. araraticumcum aestivum*	5
100	8.39	8.54	37.74	A0A0E0Q5F7	Uncharacterized protein	*O. rufipogon*	16
101	8.30	8.34	37.22	A0A1D6A150	Uncharacterized protein	*T. timopheevii subsp. araraticumcum aestivum*	5
102	8.29	8.34	35.06	A0A1D6A2L5	Uncharacterized protein	*T. timopheevii subsp. araraticumcum aestivum*	5
103	8.23	12.38	36.13	Q6EEX0	Gamma gliadin	*T. timopheevii subsp. araraticumcum aestivum*	12
104	8.22	8.27	17.77	W5D4F5	Uncharacterized protein	*T. timopheevii subsp. araraticumcum aestivum*	4
105	8.20	8.21	24.59	B6TJ90	Histone H4	*Zea mays*	4
106	8.18	20.57	32.60	R4JBK0	Low-molecular-weight glutenin subunit	*T. timopheevii subsp. araraticumcum aestivum*	42
107	8.18	8.23	19.93	A0A1D5Z6P0	rRNA N-glycosidase	*T. timopheevii subsp. araraticumcum aestivum*	4
108	8.16	12.21	42.44	D2KFH1	Avenin-like a4	*T. timopheevii subsp. araraticumcum aestivum*	18
109	8.10	10.11	45.75	A0A1D6C0D3	Uncharacterized protein	*T. timopheevii subsp. araraticumcum aestivum*	6
110	8.05	20.04	29.72	V9TRL3	High molecular weight glutenin subuni	*T. timopheevii subsp. araraticumcum aestivum*	29
111	8.03	8.61	23.14	L0L5I0	Gliadin-like avenin	*A. sativa*	10
112	8.00	8.00	29.27	Q9FVJ4	CDS_GSP-1	*Aegilops tauschii*	8
113	7.89	7.96	16.98	T1NAD0	Malate dehydrogenase	*T. timopheevii subsp. araraticumcum urartu*	4
114	7.84	13.81	63.45	R7W9W1	Alpha-amylase/trypsin inhibitor CM1	*Aegilops tauschii*	21
115	7.72	7.87	36.03	Q53WS1	Alpha 1 purothionin	*T. timopheevii subsp. araraticumcum aestivum*	5
116	7.67	11.86	67.24	M8BYH8	Non-specific lipid-transfer protein	*Aegilops tauschii*	11
117	7.64	7.75	10.67	Q41534	ATP synthase subunit beta	*T. timopheevii subsp. araraticumcum aestivum*	5
118	7.62	7.71	32.24	CAS1_BOVIN			6
119	7.54	7.62	6.07	A0A1D6REH6	Uncharacterized protein	*T. timopheevii subsp. araraticumcum aestivum*	3
120	7.37	11.63	39.69	A0A1D5XMK2	Uncharacterized protein	*T. timopheevii subsp. araraticumcum aestivum*	6
121	7.34	7.42	39.86	V5RL87	Puroindoline	*T. timopheevii subsp. araraticumcum aestivum*	7

122	7.18	7.27	38.03	W0NPU1	Vromindoline VIN3	*T. timopheevii subsp. araraticumcum aestivum*	4
123	7.11	7.25	17.62	A0A1B5GE57	Caleosin	*T. timopheevii subsp. araraticumcum aestivum*	4
124	7.09	24.38	40.86	Q7Y074	Low molecular weight glutenin	*T. timopheevii subsp. araraticumcum aestivum*	22
125	7.09	7.19	24.10	A0A1D5UB33	Uncharacterized protein	*T. timopheevii subsp. araraticumcum aestivum*	7
126	7.05	7.19	12.66	A7UME2	Xylanase inhibitor 725ACCN	*T. timopheevii subsp. araraticumcum aestivum*	4
127	7.02	7.34	1.86	M8BLL3	Pyruvate, phosphate dikinase 1, chlorop	*Aegilops tauschii*	2
128	6.99	22.21	91.13	Q5UHH7	0.19 dimeric alpha-amylase inhibitor	*T. timopheevii subsp. araraticumcum aestivum*	34
129	6.94	7.04	12.76	M8C8G6	Glyceraldehyde-3-phosphate dehydrog	*Aegilops tauschii*	3
130	6.90	32.26	55.78	A0A1D5ZBL7	Uncharacterized protein	*T. timopheevii subsp. araraticumcum aestivum*	21
131	6.84	7.01	58.97	M0V3U0	Non-specific lipid-transfer protein	*H. vulgare subsp. vulgare*	7
132	6.83	13.09	48.24	V5M0Z7	Avenin-like protein	*T. timopheevii subsp. araraticumcum aestivum*	19
133	6.83	6.90	18.38	I1PWZ3	Uncharacterized protein	*O. glaberrima*	7
134	6.74	10.06	29.25	A0A0K2QJC8	Alpha/beta-gliadin	*T. timopheevii subsp. araraticumcum aestivum*	13
135	6.55	9.60	6.19	J3MC18	Sucrose synthase	*O. brachyantha*	5
136	6.53	6.75	7.83	A0A1D5VLG8	Uncharacterized protein	*T. timopheevii subsp. araraticumcum aestivum*	3
137	6.49	8.59	42.48	W5A4Z8	Uncharacterized protein	*T. timopheevii subsp. araraticumcum aestivum*	4
138	6.47	6.81	7.59	W5I4Y2	Glucose-1-phosphate adenylyltransfera	*T. timopheevii subsp. araraticumcum aestivum*	3
139	6.36	6.39	26.74	I1PEM1	Oleosin	*O. glaberrima*	8
140	6.36	6.39	40.42	A0A1D6DLC2	Uncharacterized protein	*T. timopheevii subsp. araraticumcum aestivum*	3
141	6.31	6.39	35.14	Q712J4	Puroindoline-b	*Aegilops tauschii*	7
142	6.27	27.03	74.66	M8BV45	Alpha-amylase/trypsin inhibitor CM3	*Aegilops tauschii*	38
143	6.26	9.95	68.97	A0A1B2LQE8	A. alpha amylase trypsin inhibitor	*A. sativa*	9
144	6.25	7.70	9.34	M7YUQ6	Aspartic proteinase O.sin-1	*T. timopheevii subsp. araraticumcum urartu*	5
145	6.19	15.55	10.23	J9UF96	High molecular weight glutenin subunit	*T. timopheevii subsp. araraticumcum aestivum*	13

146	6.15	12.26	59.44	Q41540	CM 17 protein	*T. timopheevii subsp. araraticumcum aestivum*	21
147	6.13	18.73	22.22	I6TRT5	B3 hordein	*H. vulgare*	22
148	6.09	14.25	36.28	B6UKW6	Gamma-gliadin	*T. timopheevii subsp. araraticumcum aestivum*	26
149	6.07	20.80	32.04	O49258	12s globulin	*A. sativa*	26
150	6.06	6.07	8.82	A0A0D3HQM8	Non-specific lipid-transfer protein	*O. meridionalis*	4
151	6.01	6.01	27.48	Q38770	Type V Thionin	*Aegilops tauschii*	4
152	6.00	9.14	63.27	Q2PCC3	Type 2 non specific lipid transfer protei	*T. timopheevii subsp. araraticumcum aestivum*	10
153	6.00	6.00	25.15	Q93W25	Peptidyl-prolyl cis-trans isomerase	*T. timopheevii subsp. araraticumcum aestivum*	3
154	6.00	6.00	22.11	CASK_BOVIN			3
155	5.94	16.54	31.27	I0IT55	Alpha/beta-gliadin	*T. timopheevii subsp. araraticumcum aestivum*	17
156	5.88	6.28	13.99	P93636	Actin	*Zea mays*	4
157	5.87	45.72	59.27	W5EKI0	Beta-amylase	*T. timopheevii subsp. araraticumcum aestivum*	49
158	5.85	11.08	62.32	A0A0E0E948	Uncharacterized protein	*O. meridionalis*	26
159	5.84	5.97	21.88	W5BE38	Oleosin	*T. timopheevii subsp. araraticumcum aestivum*	4
160	5.82	20.84	19.44	B7U6L5	Globulin 3B	*T. timopheevii subsp. araraticumcum aestivum*	12
161	5.77	5.98	8.23	A0A1D5ZX81	Uncharacterized protein	*T. timopheevii subsp. araraticumcum aestivum*	3
162	5.76	8.44	46.34	M7ZCN8	Uncharacterized protein	*T. timopheevii subsp. araraticumcum urartu*	4
163	5.73	8.92	24.18	W5B7W5	Caleosin	*T. timopheevii subsp. araraticumcum aestivum*	5
164	5.71	5.85	7.42	W5FJT8	Tubulin beta chain	*T. timopheevii subsp. araraticumcum aestivum*	3
165	5.65	5.88	11.68	M8B1Z5	Lactoylglutathione lyase	*Aegilops tauschii*	3
166	5.60	26.51	16.86	B8ZX17	Glutenin	*T. timopheevii subsp. araraticumcum aestivum*	28
167	5.55	5.66	47.89	P81713	Bowman-Birk type trypsin inhibitor	*T. timopheevii subsp. araraticumcum aestivum*	7
168	5.54	7.68	27.61	Q2XSN3	Grain softness protein	*T. timopheevii subsp. araraticumcum aestivum*	8
169	5.51	17.84	6.45	D4HNC8	Y-type HMW glutenin subunit	*T. timopheevii subsp. araraticumcum aestivum*	20

170	5.49	5.61	16.94	D2KFH0	Gliadin/avenin-like seed protein	*T. timopheevii subsp. ararаticumcum aestivum*	3
171	5.47	5.61	9.80	W5AMI8	Uncharacterized protein	*T. timopheevii subsp. araraticumcum aestivum*	3
172	5.46	5.59	10.99	M4Q9V0	Enolase	*T. timopheevii subsp. araraticumcum aestivum*	3
173	5.39	13.70	48.00	Q6UJY8	Globulin	*T. timopheevii subsp. araraticumcum turgidum*	8
174	5.38	22.16	22.18	A0A1D5Z1A1	Uncharacterized protein	*T. timopheevii subsp. araraticumcum aestivum*	10
175	5.32	17.97	38.86	A0A0S2GJR0	Low-molecular-weight glutenin subunit	*T. timopheevii subsp. araraticumcum aestivum*	29
176	5.25	11.99	33.93	M8AQI5	Avenin-3	*T. timopheevii subsp. araraticumcum urartu*	16
177	5.22	18.78	83.06	A4GFS0	Dimeric alpha-amylase inhibitor	*Aegilops bicornis*	21
178	5.19	5.31	17.75	Q96185	Superoxide dismutase	*T. timopheevii subsp. araraticumcum aestivum*	3
179	5.19	5.26	25.22	A0A1D6ACI6	Uncharacterized protein	*T. timopheevii subsp. araraticumcum aestivum*	3
180	5.18	5.31	28.14	A0A1D5YXN3	Oleosin	*T. timopheevii subsp. araraticumcum aestivum*	6
181	5.12	5.19	9.72	A0A1D5XY09	Uncharacterized protein	*T. timopheevii subsp. araraticumcum aestivum*	3
182	5.11	5.31	26.47	M8AT68	Uncharacterized protein	*Aegilops tauschii*	3
183	5.03	15.00	68.82	Q53WS3	Em protein	*T. timopheevii subsp. araraticumcum aestivum*	9
184	4.98	5.05	6.01	J3MB04	Starch synthase, chloroplastic/amylopla	*O. brachyantha*	3
185	4.97	24.34	40.70	Q9ST58	Serpin-Z1C	*T. timopheevii subsp. araraticumcum aestivum*	16
186	4.91	20.79	17.99	A0A1D5S0Z9	Uncharacterized protein	*T. timopheevii subsp. araraticumcum aestivum*	9
187	4.90	6.32	52.00	Q5EFA3	Prolamin	*O. meridionalis*	17
188	4.89	4.95	49.38	P30569	EC protein I/II	*T. timopheevii subsp. araraticumcum aestivum*	2
189	4.81	4.88	15.58	Q8H4M5	Os07g0213600 protein	*O. sativa subsp. japonica*	2
190	4.80	4.88	32.22	W5ACP2	Uncharacterized protein	*T. timopheevii subsp. araraticumcum aestivum*	2
191	4.78	12.05	52.23	I1Q921	Uncharacterized protein	*O. glaberrima*	15
192	4.75	45.81	28.84	Q8W3X1	Low-molecular-weight glutenin subunit	*T. timopheevii subsp. araraticumcum aestivum*	68
193	4.69	27.50	36.47	A2WVB9	Putative uncharacterized protein	*O. sativa subsp. indica*	26

194	4.65	20.16	37.72	K7X1L1	Alpha-gliadin	*T. timopheevii subsp. araraticumcum aestivum*	21
195	4.65	4.80	27.81	N1QQY6	Uncharacterized protein	*Aegilops tauschii*	3
196	4.64	6.23	36.05	R4I506	Vromindoline 1.3	*A. sativa*	8
197	4.62	5.99	38.95	M8BVH7	Putative non-specific lipid-transfer prot	*Aegilops tauschii*	5
198	4.52	7.93	6.36	K22E_HUMAN			4
199	4.50	11.98	58.75	Q40653	Allergenic protein	*O. sativa subsp. japonica*	42
200	4.46	26.03	31.20	Q0E261	Glutelin	*O. sativa subsp. japonica*	22
201	4.45	4.50	10.96	O64392	T. timopheevii subsp. araraticumc	*T. timopheevii subsp. araraticumcum aestivum*	2
202	4.36	4.50	6.70	W5D5L4	Fructose-bisphosphate aldolase	*T. timopheevii subsp. araraticumcum aestivum*	2
203	4.34	4.38	12.68	Q9MB31	GSH-dependent dehydroascorbate redu	*O. sativa subsp. japonica*	2
204	4.33	4.37	15.44	P04464	Calmodulin	*T. timopheevii subsp. araraticumcum aestivum*	2
205	4.28	4.31	9.65	W5CL83	Uncharacterized protein	*T. timopheevii subsp. araraticumcum aestivum*	2
206	4.25	4.41	12.97	M8AVF3	Nuclear transport factor 2	*Aegilops tauschii*	2
207	4.17	4.30	29.32	R7W3S7	Uncharacterized protein	*Aegilops tauschii*	4
208	4.16	4.18	16.18	Q946V7	19kD alpha zein D2	*Zea mays*	3
209	4.12	4.14	8.96	I1H7M6	Uncharacterized protein	*Brachypodium distachyon*	2
210	4.08	6.24	1.37	Q7XZK7	Starch branching enzyme IIb	*Sorghum bicolor*	3
211	4.08	4.09	4.52	W5XK42	ATP synthase subunit alpha	*Aegilops longissima*	2
212	4.06	26.41	24.55	A0A1D5XS09	Uncharacterized protein	*T. timopheevii subsp. araraticumcum aestivum*	15
213	4.06	4.07	20.98	N1QTW5	Trypsin inhibitor CMc	*Aegilops tauschii*	5
214	4.05	4.05	23.89	M8C6U2	Glutaredoxin-C6	*Aegilops tauschii*	2
215	4.02	21.04	40.89	A0A1P8DSK8	Alpha-gliadin storage protein	*T. timopheevii subsp. araraticumcum aestivum*	56
216	4.02	14.03	50.00	Q8H4M4	Allergenic protein	*O. sativa subsp. japonica*	15
217	4.02	12.29	38.86	P0CZ08	Avenin-like a3	*T. timopheevii subsp. araraticumcum aestivum*	20

218	4.02	4.02	6.57	Q6ZFJ0	Os08g0502400 protein	*O. sativa subsp. japonica*	2
219	4.01	22.26	37.14	R4JAP5	Low-molecular-weight glutenin subunit	*T. timopheevii subsp. araraticumcum aestivum*	27
220	4.01	17.51	26.45	A0A0K2QJY6	Alpha/beta-gliadin	*T. timopheevii subsp. araraticumcum aestivum*	13
221	4.01	14.04	39.11	Q8S4P7	Thaumatin-like protein	*T. timopheevii subsp. araraticumcum aestivum*	7
222	4.01	13.66	22.15	Q5PU41	Low molecular weight glutenin subunit	*T. timopheevii subsp. araraticumcum aestivum*	16
223	4.00	18.44	59.83	Q6RS99	Globulin	*T. timopheevii subsp. araraticumcum turgidum*	12
224	4.00	17.56	37.02	K7X0N8	Alpha-gliadin	*T. timopheevii subsp. araraticumcum aestivum*	24
225	4.00	11.99	37.25	A0A1B2LQE9	A. alpha amylase trypsin inhibitor	*T. timopheevii subsp. araraticumcum aestivum*	7
226	4.00	10.11	42.20	Q2A780	Putative avenin-like a	*T. timopheevii subsp. araraticumcum aestivum*	22
227	4.00	8.00	26.67	A0A1D6DG60	Uncharacterized protein	*T. timopheevii subsp. araraticumcum aestivum*	4
228	4.00	4.00	28.17	M8AZ12	Non-specific lipid-transfer protein 2G	*Aegilops tauschii*	2
229	4.00	4.00	36.63	Q6ZHP6	Os02g0715400 protein	*O. sativa subsp. japonica*	2
230	4.00	4.00	22.61	M8ADF8	Uncharacterized protein	*T. timopheevii subsp. araraticumcum urartu*	2
231	4.00	4.00	18.70	A0A1D5UXT7	Cysteine proteinase inhibitor	*T. timopheevii subsp. araraticumcum aestivum*	2
232	4.00	4.00	13.17	A0A1D5SCU2|	Uncharacterized protein	*T. timopheevii subsp. araraticumcum aestivum*	2
233	3.93	10.63	11.82	Q84X93	Glutelin	*O. sativa subsp. japonica*	7
234	3.92	4.00	11.67	M7ZFM9	Uncharacterized protein	*T. timopheevii subsp. araraticumcum urartu*	2
235	3.85	4.05	5.26	A0A1D5WJB3	Uncharacterized protein	*T. timopheevii subsp. araraticumcum aestivum*	2
236	3.82	3.97	15.55	T1MEJ3	Uncharacterized protein	*T. timopheevii subsp. araraticumcum urartu*	2
237	3.74	8.00	18.86	Q38769	Permatin	*A. sativa*	4
238	3.72	3.82	19.56	M8BUR6	Uncharacterized protein	*Aegilops tauschii*	3
239	3.70	3.85	8.29	R4ZAN8	L-2	*T. timopheevii subsp. araraticumcum kiharae*	3
240	3.69	3.78	5.76	W5EI90	Uncharacterized protein	*T. timopheevii subsp. araraticumcum aestivum*	2
241	3.67	7.24	44.23	A1YQF8	Prolamin	*O. sativa subsp. japonica*	19

242	3.59	3.72	26.88	M8ATI6	Uncharacterized protein	*Aegilops tauschii*	2
243	3.53	3.61	10.06	A0A1D5RV12	Uncharacterized protein	*T. timopheevii subsp. araraticumcum aestivum*	2
244	3.52	3.68	5.16	M8BDX3	Uncharacterized protein	*Aegilops tauschii*	2
245	3.50	5.75	7.03	A0A1D5VWK9	Peroxidase	*T. timopheevii subsp. araraticumcum aestivum*	3
246	3.50	4.08	4.88	A0A0E0ETC4	Uncharacterized protein	*O. meridionalis*	2
247	3.46	3.62	4.59	W5I0B0	Uncharacterized protein	*T. timopheevii subsp. araraticumcum aestivum*	2
248	3.42	4.20	3.74	I1PE38	Uncharacterized protein	*O. glaberrima*	2
249	3.41	3.49	5.72	A0A1D6F125	UDP-arabinopyranose mutase 3	*Zea mays*	2
250	3.41	3.48	38.54	M4VP35	Uncharacterized protein	*T. timopheevii subsp. araraticumcum aestivum*	2
251	3.40	17.64	48.08	A0A1P8DT36	Alpha-gliadin storage protein	*T. timopheevii subsp. araraticumcum aestivum*	26
252	3.39	3.48	8.52	Q94LL7	Putative uncharacterized protein	*O. sativa*	2
253	3.38	3.61	7.48	W5H4V7	Phosphoglycerate kinase	*T. timopheevii subsp. araraticumcum aestivum*	2
254	3.33	3.47	17.24	A0A1D6B171	Uncharacterized protein	*T. timopheevii subsp. araraticumcum aestivum*	2
255	3.28	10.81	61.05	I1PUP1	Uncharacterized protein	*O. glaberrima*	6
256	3.24	24.57	32.33	R9XUM8	Alpha-gliadin	*T. timopheevii subsp. araraticumcum aestivum*	28
257	3.24	3.33	2.29	D0TZH2	Pullulanase	*O. sativa subsp. indica*	2
258	3.22	7.64	21.18	Q7Y1Z2	27K protein	*T. timopheevii subsp. araraticumcum aestivum*	5
259	3.19	3.38	12.35	W5CY88	Uncharacterized protein	*T. timopheevii subsp. araraticumcum aestivum*	2
260	3.17	7.06	22.82	A0A1D5SF46	Uncharacterized protein	*T. timopheevii subsp. araraticumcum aestivum*	4
261	3.14	12.48	56.96	B8B8F6	Putative uncharacterized protein	*O. sativa subsp. indica*	13
262	3.14	3.25	18.60	W5E549	Uncharacterized protein	*T. timopheevii subsp. araraticumcum aestivum*	2
263	3.12	3.34	5.32	R7W8V8	Tubulin alpha chain	*Aegilops tauschii*	2
264	3.11	3.26	6.62	Q0WX48	Thaumatin-like xylanase inhibitor	*T. timopheevii subsp. araraticumcum aestivum*	1
265	3.07	12.79	63.83	M7YEH7	Uncharacterized protein	*T. timopheevii subsp. araraticumcum urartu*	15

266	3.06	3.13	4.65	A0A1D6S518	Uncharacterized protein	*T. timopheevii subsp. araraticumcum aestivum*	2
267	3.04	3.10	5.49	B6SYN0	Putative uncharacterized protein	*Zea mays*	4
268	3.02	19.65	45.30	B6UKP6	Gamma-gliadin	*T. timopheevii subsp. araraticumcum aestivum*	31
269	3.01	8.34	56.86	Q5W740	Os05g0332000 protein	*O. sativa subsp. japonica*	12
270	3.01	4.38	12.62	W5BUF4	Caleosin	*T. timopheevii subsp. araraticumcum aestivum*	3
271	3.00	3.07	8.81	W5ECL2	Uncharacterized protein	*T. timopheevii subsp. araraticumcum aestivum*	2
272	2.96	4.24	34.00	M0WPC3	Uncharacterized protein	*H. vulgare subsp. vulgare*	5
273	2.94	15.74	36.79	W8Q671	Farinin protein	*Brachypodium distachyon*	28
274	2.94	12.04	28.48	R9XU99	Gamma-gliadin	*T. timopheevii subsp. araraticumcum aestivum*	14
275	2.91	3.10	7.26	A0A1D6BTL4	Uncharacterized protein	*T. timopheevii subsp. araraticumcum aestivum*	1
276	2.89	3.03	13.89	M8CGQ1	Uncharacterized protein	*Aegilops tauschii*	2
277	2.88	16.34	44.25	A0A0E0Q5M4	Uncharacterized protein	*O. rufipogon*	52
278	2.88	15.95	12.43	J9Q8Q6	High molecular weight glutenin subunit	*T. timopheevii subsp. araraticumcum dicoccoides*	16
279	2.85	6.41	30.54	Q41518	Single-stranded nucleic acid binding pro	*T. timopheevii subsp. araraticumcum aestivum*	5
280	2.82	2.92	9.07	W5ERW2	Uncharacterized protein	*T. timopheevii subsp. araraticumcum aestivum*	2
281	2.78	2.85	16.59	Q8LPA7	Cold shock protein-1	*T. timopheevii subsp. araraticumcum aestivum*	2
282	2.75	7.51	8.00	Q3YAF9	B hordein	*H. vulgare subsp. vulgare*	4
283	2.74	21.55	36.39	A0A0E3Z7F7	Alpha-gliadin	*T. timopheevii subsp. araraticumcum aestivum*	26
284	2.74	2.83	7.32	A0A1D5RU62	Uncharacterized protein	*T. timopheevii subsp. araraticumcum aestivum*	1
285	2.73	2.80	16.45	W5B1E5	Superoxide dismutase	*T. timopheevii subsp. araraticumcum aestivum*	2
286	2.72	3.00	4.20	W5DYH1	Uncharacterized protein	*T. timopheevii subsp. araraticumcum aestivum*	1
287	2.70	2.79	2.29	Q9ZTP0	Putative uncharacterized protein	*O. sativa*	1
288	2.68	15.19	16.19	V9P760	LMW-m glutenin subunit 45	*T. timopheevii subsp. araraticumcum aestivum*	12
289	2.66	7.12	38.00	M8ANS4	Avenin-3	*T. timopheevii subsp. araraticumcum urartu*	13

290	2.62	2.92	5.01	N1QTR7	Uncharacterized protein	*Aegilops tauschii*	2
291	2.61	2.65	27.72	A0A1D6BFJ3	Uncharacterized protein	*T. timopheevii subsp. araraticumcum aestivum*	1
292	2.59	4.42	22.79	B8YM21	Beta purothionin	*T. timopheevii subsp. araraticumcum urartu*	3
293	2.57	8.77	59.00	A0A1D6BIB2	Uncharacterized protein	*T. timopheevii subsp. araraticumcum aestivum*	6
294	2.53	17.87	38.93	W8PUU6	Farinin protein	*Brachypodium distachyon*	27
295	2.50	2.63	15.33	M8ARU5	Nucleoside diphosphate kinase	*T. timopheevii subsp. araraticumcum urartu*	2
296	2.46	11.15	34.11	P27919	Avenin	*A. sativa*	11
297	2.45	2.58	8.43	R7W9M2	Putative aquaporin TIP3-1	*Aegilops tauschii*	2
298	2.45	2.50	6.00	K3ZUR5	Thiamine thiazole synthase, chloroplast	*Setaria italica*	1
299	2.44	4.30	4.68	A0A0E0P2F5	Uncharacterized protein	*O. rufipogon*	2
300	2.43	2.48	2.55	Q9FPK6	Aldehyde dehydrogenase	*O. sativa*	1
301	2.42	7.48	18.95	Q41603	LMW glutenin	*T. timopheevii subsp. araraticumcum turgidum subsp. durum*	11
302	2.41	2.46	1.69	A0A1D5WQ92	Uncharacterized protein	*T. timopheevii subsp. araraticumcum aestivum*	1
303	2.41	2.46	2.16	W5B347	Uncharacterized protein	*T. timopheevii subsp. araraticumcum aestivum*	1
304	2.41	2.45	9.59	Q65XV6	Os05g0111200 protein	*O. sativa subsp. japonica*	2
305	2.40	2.44	9.32	M7ZEK8	Uncharacterized protein	*T. timopheevii subsp. araraticumcum urartu*	1
306	2.35	7.22	29.46	W5FLF3	Uncharacterized protein	*T. timopheevii subsp. araraticumcum aestivum*	5
307	2.35	2.46	16.34	A0A1E5V9X3	Late embryogenesis abundant protein B	*T. timopheevii subsp. araraticumcum aestivum*	3
308	2.35	2.39	22.12	W4ZQ59	Uncharacterized protein	*T. timopheevii subsp. araraticumcum aestivum*	2
309	2.32	6.28	20.15	J7HYA4	Gamma-gliadin	*Aegilops umbellulata*	11
310	2.31	9.42	25.94	A0A1D5ZYI0	Uncharacterized protein	*T. timopheevii subsp. araraticumcum aestivum*	4
311	2.26	2.29	6.50	T1NPR1	Uncharacterized protein	*T. timopheevii subsp. araraticumcum urartu*	1
312	2.23	2.41	3.03	Q41579	Rab protein	*T. timopheevii subsp. araraticumcum aestivum*	1
313	2.18	6.49	25.00	Q9FVJ5	GSP-A1	*T. timopheevii subsp. araraticumcum aestivum*	7

314	2.18	2.20	14.29	R7W7L2	Uncharacterized protein	*Aegilops tauschii*	1
315	2.17	2.32	10.22	M8A2L5	Uncharacterized protein	*T. timopheevii subsp. ararаticumcum urartu*	2
316	2.16	2.26	5.70	A0A1D6CJL6	Uncharacterized protein	*T. timopheevii subsp. araraticumcum aestivum*	1
317	2.15	2.17	3.81	W5F815	Uncharacterized protein	*T. timopheevii subsp. araraticumcum aestivum*	1
318	2.14	7.02	12.76	Q43359	Cytosolic glyceroldehyde-3-phosphate	*Zea mays*	3
319	2.12	2.99	2.12	Q9ARI0	ADP-glucose pyrophosphorylase large s	*O. sativa subsp. japonica*	1
320	2.10	3.02	16.14	Q548E9	27 kDa gamma-zein	*Zea mays*	4
321	2.09	2.10	5.56	W5AR77	Uncharacterized protein	*T. timopheevii subsp. araraticumcum aestivum*	1
322	2.08	2.09	8.33	F2DWT1	Predicted protein	*H. vulgare subsp. vulgare*	1
323	2.06	4.09	49.38	P30570	EC protein III	*T. timopheevii subsp. araraticumcum aestivum*	2
324	2.06	3.96	12.42	F2DYL9	Predicted protein	*H. vulgare subsp. vulgare*	2
325	2.06	2.07	5.51	W5FQE2	Uncharacterized protein	*T. timopheevii subsp. araraticumcum aestivum*	1
326	2.06	2.07	8.18	R7W4C4	Subtilisin-chymotrypsin inhibitor-2A	*Aegilops tauschii*	1
327	2.05	10.12	47.22	A0A1B2LQB5	A. alpha amylase trypsin inhibitor	*T. timopheevii subsp. araraticumcum aestivum*	9
328	2.05	2.18	8.21	A0A1D5WYI6	Uncharacterized protein	*T. timopheevii subsp. araraticumcum aestivum*	2
329	2.05	2.06	7.69	W5F7Q9	Uncharacterized protein	*T. timopheevii subsp. araraticumcum aestivum*	1
330	2.04	3.33	14.46	A0A1D5Y5R8	Uncharacterized protein	*T. timopheevii subsp. araraticumcum aestivum*	2
331	2.04	2.05	10.14	A0A1D6DLM3	Uncharacterized protein	*T. timopheevii subsp. araraticumcum aestivum*	1
332	2.03	9.64	64.58	Q9FEK9	Lipid transfer protein	*T. timopheevii subsp. araraticumcum turgidum subsp. durum*	11
333	2.03	5.17	36.62	R4I3I8	Vromindoline 3	*A. sativa*	4
334	2.03	2.04	6.44	N1QWN7	Early nodulin-like protein 2	*Aegilops tauschii*	1
335	2.03	2.04	9.86	LALBA_BOVIN			1
336	2.03	2.03	15.91	R7W3F1	Uncharacterized protein	*Aegilops tauschii*	1
337	2.02	49.95	38.78	Q75ZV9	LMW-s HS1	*T. timopheevii subsp. araraticumcum aestivum*	88

338	2.02	7.26	34.58	F2EE76	Predicted protein	*H. vulgare subsp. vulgare*	4
339	2.01	16.88	9.47	D7RT26	HMW glutenin subunit Gx	*T. timopheevii subsp. araraticum*	18
340	2.01	12.79	34.68	A0A0E3UQX3	Alpha-gliadin	*T. timopheevii subsp. araraticumcum aestivum*	18
341	2.01	7.75	24.18	Q6L5L7	Embryonic abundant protein	*Bromus inermis*	4
342	2.00	45.19	63.62	M8B5G5	Beta-amylase	*T. timopheevii subsp. araraticumcum urartu*	45
343	2.00	39.37	32.57	H9XH01	Low-molecular-weight glutenin subunit	*T. timopheevii subsp. araraticumcum aestivum*	61
344	2.00	35.32	34.36	Q52NZ5	Low molecular weight glutenin subunit	*T. timopheevii subsp. araraticumcum aestivum*	43
345	2.00	34.64	32.89	H6VLQ4	LMW-GS	*T. timopheevii subsp. araraticumcum aestivum*	41
346	2.00	33.83	29.43	A0A0X9BSF8	High molecular weight glutenin subunit	*T. timopheevii subsp. araraticumcum aestivum*	38
347	2.00	32.79	32.47	X2JBS3	Low-molecular-weight glutenin subunit	*T. timopheevii subsp. araraticumcum aestivum*	43
348	2.00	22.66	33.33	Q0Q5D8	High-molecular-weight glutenin By8	*T. timopheevii subsp. araraticumcum aestivum*	40
349	2.00	21.84	16.48	Q8RVX0	High molecular weight glutenin subunit	*T. timopheevii subsp. araraticumcum turgidum subsp. durum*	26
350	2.00	21.08	12.51	X2JUA0	High molecular weight glutenin subunit	*T. timopheevii subsp. araraticumcum aestivum*	31
351	2.00	20.54	64.24	C4P5J4	Monomeric alpha-amylase inhibitor	*T. timopheevii subsp. araraticumcum dicoccoides*	17
352	2.00	20.18	57.39	A0A173DQZ7	Type-b avenin-like protein	*T. timopheevii subsp. araraticumcum aestivum*	25
353	2.00	19.12	22.77	D4HNC3	Low molecular weight glutenin subunit	*Taeniatherum caput-medusae subsp. crinitum*	14
354	2.00	18.88	29.84	A5JTR3	Alpha-gliadin Gli-Ts4	*T. timopheevii subsp. araraticumcum aestivum*	25
355	2.00	18.46	64.24	C4P5B7	Monomeric alpha-amylase inhibitor	*T. timopheevii subsp. araraticumcum dicoccoides*	18
356	2.00	17.11	18.80	D0EVP4	LMW-m glutenin subunit	*T. timopheevii subsp. araraticumcum aestivum*	12
357	2.00	14.29	46.27	A0A1D6CWE1	Uncharacterized protein	*T. timopheevii subsp. araraticumcum aestivum*	31
358	2.00	13.34	13.97	J3LBL1	Uncharacterized protein	*O. brachyantha*	7
359	2.00	13.18	43.65	Q2A782	Putative avenin-like a	*Aegilops cylindrica*	22
360	2.00	12.47	32.05	I4EP57	Avenin	*T. timopheevii subsp. araraticumcum aestivum*	21

361	2.00	11.69	65.79	Q7XAH1	High-molecular-weight glutenin subunit	*T. timopheevii subsp. araraticumcum aestivum*	13
362	2.00	11.56	63.51	A0A1B2LQD0	A. alpha amylase trypsin inhibitor-2	*T. timopheevii subsp. araraticumcum aestivum*	6
363	2.00	10.19	30.74	A0A0E0E2C5	Uncharacterized protein	*O. meridionalis*	17
364	2.00	9.35	30.63	Q09072	Avenin	*A. sativa*	15
365	2.00	9.32	3.35	A0A0E0EED3	Sucrose synthase	*O. meridionalis*	4
366	2.00	8.97	25.73	G8ZCW5	Avenin protein	*T. timopheevii subsp. araraticumcum aestivum*	13
367	2.00	8.72	28.34	A0A1D5XQU6	Oleosin	*T. timopheevii subsp. araraticumcum aestivum*	10
368	2.00	8.00	28.77	M8A1S2	Trypsin/alpha-amylase inhibitor CMX1/	*T. timopheevii subsp. araraticumcum urartu*	5
369	2.00	8.00	21.66	A0A1D5X2J6	Uncharacterized protein	*T. timopheevii subsp. araraticumcum aestivum*	4
370	2.00	6.52	2.53	I1PVJ3	Pyruvate, phosphate dikinase	*O. glaberrima*	2
371	2.00	4.99	5.78	W0G9U6	Starch synthase, chloroplastic/amylopla	*T. timopheevii subsp. araraticumcum urartu*	3
372	2.00	4.58	17.67	A0A1D5V3W0	Oleosin	*T. timopheevii subsp. araraticumcum aestivum*	6
373	2.00	4.57	9.48	Q670S2	B hordein	*T. timopheevii subsp. araraticumcum aestivum*	4
374	2.00	4.19	4.33	Q401N7	Aspartic proteinase	*T. timopheevii subsp. araraticumcum aestivum*	3
375	2.00	4.02	12.99	A0A1D6AS82	Uncharacterized protein	*T. timopheevii subsp. araraticumcum aestivum*	2
376	2.00	4.00	39.13	A0A1D5XXQ6	Uncharacterized protein	*T. timopheevii subsp. araraticumcum aestivum*	2
377	2.00	4.00	15.54	S4U0K5	Oleosin	*O. sativa subsp. japonica*	2
378	2.00	2.72	30.72	Q0DJ38	Os05g0331800 protein	*O. sativa subsp. japonica*	8
379	2.00	2.25	7.33	A0A1D5WXL8	Uncharacterized protein	*T. timopheevii subsp. araraticumcum aestivum*	2
380	2.00	2.20	10.83	Q9SQG8	Pathogenesis-related protein 4	*T. timopheevii subsp. araraticumcum aestivum*	1
381	2.00	2.07	5.12	Q94JF2	Os01g0705200 protein	*O. sativa subsp. japonica*	1
382	2.00	2.00	5.06	Q946W0	15kD beta zein	*Zea mays*	2
383	2.00	2.00	6.58	I1H1D2	Uncharacterized protein	*Brachypodium distachyon*	1
384	2.00	2.00	1.77	A0A0E0A6E7	Uncharacterized protein	*O. meridionalis*	1

385	2.00	2.00	13.92	W5G4V0	Uncharacterized protein	*T. timopheevii subsp. araraticumcum aestivum*	1
386	2.00	2.00	7.51	W5G1S5	Uncharacterized protein	*T. timopheevii subsp. araraticumcum aestivum*	1
387	2.00	2.00	20.27	W5CTA6	Uncharacterized protein	*T. timopheevii subsp. araraticumcum aestivum*	1
388	2.00	2.00	3.31	W5BQF4	Lactoylglutathione lyase	*T. timopheevii subsp. araraticumcum aestivum*	1
389	2.00	2.00	3.53	W5BGU3	Uncharacterized protein	*T. timopheevii subsp. araraticumcum aestivum*	1
390	2.00	2.00	8.18	W5BFB7	Uncharacterized protein	*T. timopheevii subsp. araraticumcum aestivum*	1
391	2.00	2.00	1.57	W5B5M5	Uncharacterized protein	*T. timopheevii subsp. araraticumcum aestivum*	1
392	2.00	2.00	5.58	T1MRQ2	Uncharacterized protein	*T. timopheevii subsp. araraticumcum urartu*	1
393	2.00	2.00	12.94	R7WFJ0	Uncharacterized protein	*Aegilops tauschii*	1
394	2.00	2.00	8.48	R7WDJ9	Uncharacterized protein	*Aegilops tauschii*	1
395	2.00	2.00	3.26	R7WD67	Malate dehydrogenase	*Aegilops tauschii*	1
396	2.00	2.00	7.14	Q9AV77	60S ribosomal protein L17	*O. sativa subsp. japonica*	1
397	2.00	2.00	39.24	Q95951	ATP synthase subunit 9, mitochondrial	*Sorghum bicolor*	1
398	2.00	2.00	6.44	Q946W1	50kD gamma zein	*Zea mays*	1
399	2.00	2.00	3.14	Q7XM93	OSJNBb0060E08.6 protein	*O. sativa subsp. japonica*	1
400	2.00	2.00	5.18	Q6Z4W7	Os08g0273300 protein	*O. sativa subsp. japonica*	1
401	2.00	2.00	8.85	Q65WT5	Os05g0445500 protein	*O. sativa subsp. japonica*	1
402	2.00	2.00	2.19	Q5NA76	Putative uncharacterized protein B1066	*O. sativa subsp. japonica*	1
403	2.00	2.00	5.03	Q06I75	Fasciclin-like protein FLA31	*T. timopheevii subsp. araraticumcum aestivum*	1
404	2.00	2.00	1.48	M8BVK5	Uncharacterized protein	*Aegilops tauschii*	1
405	2.00	2.00	4.35	J3LXU1	Uncharacterized protein	*O. brachyantha*	1
406	2.00	2.00	5.09	I6TEV2	Gamma 3 hordein	*H. vulgare*	1
407	2.00	2.00	2.21	I1IGR0	Uncharacterized protein	*Brachypodium distachyon*	1
408	2.00	2.00	0.90	I1GVI1	Uncharacterized protein	*Brachypodium distachyon*	1

409	2.00	2.00	6.99	F4ZL28	Alpha-gliadin storage protein	*Secale strictum subsp. africanum*	1
410	2.00	2.00	3.33	B6SI53	Fructose-bisphosphate aldolase	*Zea mays*	1
411	2.00	2.00	15.19	A0A1D6S463	Uncharacterized protein	*T. timopheevii subsp. araraticumcum aestivum*	1
412	2.00	2.00	1.14	A0A1D6CYE6	Uncharacterized protein	*T. timopheevii subsp. araraticumcum aestivum*	1
413	2.00	2.00	5.77	A0A1D6CU02	Uncharacterized protein	*T. timopheevii subsp. araraticumcum aestivum*	1
414	2.00	2.00	19.54	A0A1D6B8M4	Uncharacterized protein	*T. timopheevii subsp. araraticumcum aestivum*	1
415	2.00	2.00	12.40	A0A0Q3PA99	Uncharacterized protein	*Brachypodium distachyon*	1
416	2.00	2.00	5.21	A0A0E0JM09	Uncharacterized protein	*O. punctata*	1
417	2.00	2.00	4.32	A0A077RPK1	Uncharacterized protein	*T. timopheevii subsp. araraticumcum aestivum*	1
418	2.00	2.00	1.38	C5XMS9	Ubiquitinyl hydrolase 1	*Sorghum bicolor*	1
419	1.96	2.00	2.64	W5EPR0	Uncharacterized protein	*T. timopheevii subsp. araraticumcum aestivum*	1
420	1.96	2.00	6.96	Q6K2D2	Oleosin	*O. sativa subsp. japonica*	1
421	1.94	21.45	36.65	E0Z2G5	Alpha-gliadin storage protein	*Aegilops tauschii*	20
422	1.92	2.00	6.94	W5FEZ3	40S ribosomal protein S12	*T. timopheevii subsp. araraticumcum aestivum*	1
423	1.89	2.20	4.03	R7WFJ2	Uncharacterized protein	*Aegilops tauschii*	1
424	1.89	2.04	1.86	W5B7Z6	Adenosylhomocysteinase	*T. timopheevii subsp. araraticumcum aestivum*	1
425	1.85	14.03	28.07	M0Y227	Uncharacterized protein	*H. vulgare subsp. vulgare*	13
426	1.85	2.04	5.09	W5CKU7	Uncharacterized protein	*T. timopheevii subsp. araraticumcum aestivum*	1
427	1.85	2.00	1.16	W5G7S7	Uncharacterized protein	*T. timopheevii subsp. araraticumcum aestivum*	1
428	1.82	10.10	14.66	R4JF25	Low-molecular-weight glutenin subunit	*T. timopheevii subsp. araraticumcum aestivum*	12
429	1.80	2.00	3.23	W5CWR9	Uncharacterized protein	*T. timopheevii subsp. araraticumcum aestivum*	1
430	1.80	1.92	17.74	Q2TM68	Metallothionein-like protein	*O. meridionalis*	1
431	1.80	1.92	32.00	A0A060AQ88	Defensin	*T. timopheevii subsp. araraticumcum aestivum*	1
432	1.78	12.62	24.17	I0IT62	Alpha/beta-gliadin	*T. timopheevii subsp. araraticumcum aestivum*	15

433	1.75	4.06	9.46	C5X3B9	Uncharacterized protein	*Sorghum bicolor*	2
434	1.74	15.54	39.83	A0A0K2QJE4	Pseudo alpha/beta-gliadin	*T. timopheevii subsp. araraticumcum aestivum*	15
435	1.74	1.89	1.14	A0A1D6MED7	Structural maintenance of chromosome	*Zea mays*	1
436	1.74	1.89	4.01	A0A0D9UVQ5	Uncharacterized protein	*O. meridionalis*	1
437	1.73	12.88	59.77	Q94G97	Gamma-gliadin	*T. timopheevii subsp. araraticumcum aestivum*	28
438	1.72	1.96	17.41	CASB_BOVIN			4
439	1.72	1.93	1.50	M7YE46	Vicilin-like antimicrobial peptides 2-2	*T. timopheevii subsp. araraticumcum urartu*	1
440	1.71	5.52	46.67	Q9S6Y2	Alpha purothionin	*T. timopheevii subsp. araraticumcum aestivum*	4
441	1.70	13.81	58.23	A0A0E0E956	Uncharacterized protein	*O. meridionalis*	17
442	1.69	1.84	2.44	Q6ZBH2	Os08g0545200 protein	*O. sativa subsp. japonica*	1
443	1.68	17.03	25.00	Q5XXZ7	Low molecular weight glutenin subunit	*Elymus elongatus*	11
444	1.68	2.01	5.20	C0PPC9	Uncharacterized protein	*Zea mays*	2
445	1.68	1.82	15.63	Q40051	Glycine rich protein	*H. vulgare*	1
446	1.64	1.90	8.08	I1P9U0	Uncharacterized protein	*O. glaberrima*	1
447	1.64	1.87	4.30	BGAL_ECOLI			2
448	1.64	1.77	2.00	W5A2F4	Uncharacterized protein	*T. timopheevii subsp. araraticumcum aestivum*	1
449	1.62	7.53	41.03	A0A0E0RFS6	Uncharacterized protein	*O. rufipogon*	26
450	1.62	2.79	5.79	A0A0Q3KAB9	Uncharacterized protein	*Brachypodium distachyon*	2
451	1.61	2.79	35.76	A0A0E0HY57	Uncharacterized protein	*O. nivara*	12
452	1.60	1.74	7.07	C0PNC3	Uncharacterized protein	*Zea mays*	1
453	1.59	1.81	1.83	M0YZF2	Uncharacterized protein	*H. vulgare subsp. vulgare*	1
454	1.59	1.72	8.93	K3ZY88	Uncharacterized protein	*Setaria italica*	1
455	1.58	28.59	43.31	A0A0E3Z6U5	Alpha-gliadin	*T. timopheevii subsp. araraticumcum aestivum*	29
456	1.57	1.70	5.77	M0XN12	Uncharacterized protein	*H. vulgare subsp. vulgare*	1

457	1.56	14.07	10.20	W5DYF8	Uncharacterized protein	*T. timopheevii subsp. araraticumcum aestivum*	7
458	1.54	1.68	5.34	W5BGL0	Uncharacterized protein	*T. timopheevii subsp. araraticumcum aestivum*	1
459	1.53	1.76	1.19	Q7XTK1	Elongation factor	*O. sativa subsp. japonica*	1
460	1.52	1.66	3.85	Q6R985	Cytochrome c oxidase subunit 2	*Zea mays*	1
461	1.52	1.66	3.79	M8BK19	Glucan endo-1,3-beta-glucosidase GIV	*Aegilops tauschii*	1
462	1.49	8.43	28.34	A0A1D6D697	Uncharacterized protein	*T. timopheevii subsp. araraticumcum aestivum*	5
463	1.48	1.62	66.67	Q9S8W4	Alpha-amylase inhibitor	*A. sativa*	2
464	1.47	41.58	24.15	A0A0U3AGE0	Low molecular weight glutenin subunit	*Aegilops searsii*	61
465	1.47	1.67	20.27	W5HIB6	Defensin	*T. timopheevii subsp. araraticumcum aestivum*	1
466	1.47	1.62	5.29	W5I1R7	Uncharacterized protein	*T. timopheevii subsp. araraticumcum aestivum*	1
467	1.46	1.77	0.00	M8B1C1	10 kDa chaperonin	*Aegilops tauschii*	0
468	1.45	1.68	1.36	A0A0E0PRJ8	Uncharacterized protein	*O. rufipogon*	1
469	1.45	1.59	3.13	M8A6E3	Bowman-Birk type trypsin inhibitor	*T. timopheevii subsp. araraticumcum urartu*	1
470	1.44	1.63	1.21	W5G4R7	Pyrophosphate--fructose 6-phosphate 1	*T. timopheevii subsp. araraticumcum aestivum*	1
471	1.44	1.59	2.25	A0A0D9XVQ3	Uncharacterized protein	*O. meridionalis*	1
472	1.44	1.57	12.36	W5DZY1	Uncharacterized protein	*T. timopheevii subsp. araraticumcum aestivum*	1
473	1.42	24.46	29.72	Q6UJY7	HMW-glutenin By subunit	*T. timopheevii subsp. araraticumcum turgidum*	42
474	1.40	1.60	2.01	Q5VPB9	Ketol-acid reductoisomerase	*O. sativa subsp. japonica*	1
475	1.38	1.51	20.45	W5EJM2	Uncharacterized protein	*T. timopheevii subsp. araraticumcum aestivum*	1
476	1.38	1.51	8.91	R7WAY7	Carbonic anhydrase	*Aegilops tauschii*	1
477	1.37	22.22	25.41	A0A1D5YEH1	Uncharacterized protein	*T. timopheevii subsp. araraticumcum aestivum*	12
478	1.35	25.74	23.20	M8BGV8	Globulin-1 S allele	*Aegilops tauschii*	14
479	1.35	1.49	4.40	W5H0R7	Uncharacterized protein	*T. timopheevii subsp. araraticumcum aestivum*	1
480	1.33	1.46	13.41	W5EL62	40S ribosomal protein S21	*T. timopheevii subsp. araraticumcum aestivum*	1

481	1.33	1.46	2.88	A0A1D5UMX4	REVERSED Uncharacterized protein	*T. timopheevii subsp. araraticumcum aestivum*	1
482	1.30	1.43	4.92	W5B757	Uncharacterized protein	*T. timopheevii subsp. araraticumcum aestivum*	1
483	1.28	1.40	12.12	W5EPH9	Uncharacterized protein	*T. timopheevii subsp. araraticumcum aestivum*	1
484	1.26	1.45	2.50	K3YFC9	Uncharacterized protein	*Setaria italica*	1
485	1.25	1.38	5.88	A0A1D6DLJ4	Uncharacterized protein	*T. timopheevii subsp. araraticumcum aestivum*	1
486	1.25	1.38	2.34	I1NYL5	Uncharacterized protein	*O. glaberrima*	1
487	1.24	1.35	4.43	W5BMW7	Uncharacterized protein	*T. timopheevii subsp. araraticumcum aestivum*	1
488	1.22	12.08	27.88	J7HYD9	Gamma-gliadin	*Aegilops speltoides*	16
489	1.22	8.38	15.26	W5E2W7	40S ribosomal protein SA	*T. timopheevii subsp. araraticumcum aestivum*	4
490	1.21	11.09	34.93	A0A1D5SXD6	Uncharacterized protein	*T. timopheevii subsp. araraticumcum aestivum*	8
491	1.21	1.41	3.93	A0A1D5ZZJ8	Uncharacterized protein	*T. timopheevii subsp. araraticumcum aestivum*	1
492	1.20	3.96	12.22	W5AC28	Uncharacterized protein	*T. timopheevii subsp. araraticumcum aestivum*	3
493	1.20	1.54	23.50	Q548E8	16 kDa gamma zein	*Zea mays*	4
494	1.16	1.30	4.76	A0A0E0EH66	Uncharacterized protein	*O. meridionalis*	1
495	1.15	1.42	10.14	W5EM63	Uncharacterized protein	*T. timopheevii subsp. araraticumcum aestivum*	1
496	1.15	1.27	7.45	W5AKH4	Uncharacterized protein	*T. timopheevii subsp. araraticumcum aestivum*	1
497	1.14	3.97	10.00	I1QCJ2	Uncharacterized protein	*O. glaberrima*	2
498	1.13	10.48	15.21	I1HMK8	Uncharacterized protein	*Brachypodium distachyon*	8
499	1.13	3.53	5.65	A0A1D5WVH9	Uncharacterized protein	*T. timopheevii subsp. araraticumcum aestivum*	2
500	1.13	1.26	1.59	W5G112	Uncharacterized protein	*T. timopheevii subsp. araraticumcum aestivum*	1
501	1.13	1.24	4.10	K3YU83	Uncharacterized protein	*Setaria italica*	1
502	1.12	1.24	23.26	A0A0A8ZQG3	Uncharacterized protein	*Arundo donax*	1
503	1.10	1.29	7.50	W5I301	Uncharacterized protein	*T. timopheevii subsp. araraticumcum aestivum*	1
504	1.10	1.22	11.49	W5GVJ0	Uncharacterized protein	*T. timopheevii subsp. araraticumcum aestivum*	1

505	1.09	1.20	4.41	A0A1D5WVJ7	Uncharacterized protein	*T. timopheevii subsp. araraticumcum aestivum*	1
506	1.07	26.65	17.18	A0A142ESP3	High molecular weight glutenin subunit	*T. timopheevii subsp. araraticumcum dicoccoides*	29
507	1.06	1.18	11.65	M8CFP1	Non-specific lipid-transfer protein 2G	*Aegilops tauschii*	1
508	1.06	1.18	5.33	W5AXM7	Uncharacterized protein	*T. timopheevii subsp. araraticumcum aestivum*	1
509	1.05	8.29	27.49	G8ZCW4	Avenin protein	*T. timopheevii subsp. araraticumcum aestivum*	14
510	1.05	1.16	15.49	M7YGF5	Subtilisin-chymotrypsin inhibitor-2A	*T. timopheevii subsp. araraticumcum urartu*	1
511	1.03	1.15	12.50	W5HAI5	Uncharacterized protein	*T. timopheevii subsp. araraticumcum aestivum*	1
512	1.01	5.89	6.72	D4AIA3	Glucose-1-phosphate adenylyltransfera	*O. sativa subsp. indica*	3
513	1.01	1.17	2.71	A0A1D5V1B7	Uncharacterized protein	*T. timopheevii subsp. araraticumcum aestivum*	1
514	1.00	3.03	4.91	R4ZCU4	L-1	*T. timopheevii subsp. araraticum*	2
515	1.00	1.11	1.59	N1R4J0	Uncharacterized protein	*Aegilops tauschii*	1
516	0.99	1.17	10.98	A0A1D5SJ31	Uncharacterized protein	*T. timopheevii subsp. araraticumcum aestivum*	1
517	0.98	1.11	1.70	W5AC96	Carboxypeptidase	*T. timopheevii subsp. araraticumcum aestivum*	1
518	0.97	3.20	3.53	A0A1E5UKF8	Isocitrate dehydrogenase	*Dichanthelium oligosanthes*	2
519	0.97	1.10	3.91	W5BUA4	Uncharacterized protein	*T. timopheevii subsp. araraticumcum aestivum*	1
520	0.95	9.44	31.86	W5DWP8	Uncharacterized protein	*T. timopheevii subsp. araraticumcum aestivum*	6
521	0.95	1.06	10.00	Q5I7K5	Ribosomal protein P1	*T. timopheevii subsp. araraticumcum aestivum*	1
522	0.94	1.06	7.23	Q2TN84	USP family protein	*T. timopheevii subsp. araraticumcum aestivum*	1
523	0.93	1.09	2.87	A0A1D6DIS2	Uncharacterized protein	*T. timopheevii subsp. araraticumcum aestivum*	1
524	0.92	1.03	2.28	W5GHC3	Uncharacterized protein	*T. timopheevii subsp. araraticumcum aestivum*	1

Supplementary Table 3. Protein identifications in milk-based breakfast drinks (BM). Trypsin digestion peptides were identified after database searching against the Poaceae subset of the Uniprot database appended with the Common Repository of Adventitious Protein (cRAP) database using a 1% global false discovery rate (FDR) threshold.

N	Unused Score	Total Score	% Cov (95%)	Accession	Name	Species	Peptides (95%)
1	98.32	98.32	74.30	ALBU_BOVIN	Albumin	*Bos taurus*	67
2	73.35	73.35	73.36	CAS1_BOVIN	Casein	*Bos taurus*	280
3	62.81	62.81	72.97	CAS2_BOVIN	Casein	*Bos taurus*	108
4	49.61	49.61	64.74	CASK_BOVIN	Casein	*Bos taurus*	136
5	39.06	39.06	76.06	LALBA_BOVIN	Lactalbumin	*Bos taurus*	95
6	28.22	28.22	21.91	K3XFF0	Uncharacterized protein	*Setaria italica*	14
7	27.73	27.75	73.66	CASB_BOVIN	Casein	*Bos taurus*	64
8	23.38	23.38	40.58	W5EE00	Uncharacterized protein	*T. aestivum*	12
9	22.95	22.97	25.68	P14812	12S seed storage globulin 2	*A. sativa*	12
10	20.90	20.90	29.53	Q8SAT2	Elongation factor 1-alpha	*Saccharum officinarum*	13
11	20.77	20.77	32.75	M0UEE6	Uncharacterized protein	*H. vulgare subsp. vulgare*	13
12	18.59	18.59	66.67	M0V3U0	Non-specific lipid-transfer protein	*H. vulgare subsp. vulgare*	24
13	16.40	16.40	13.29	F4Y5B1	Heat shock protein 81-3	*Aegilops tauschii*	10
14	16.03	16.03	11.36	W5ET88	Uncharacterized protein	*T. aestivum*	8
15	15.91	18.03	15.93	A0A194YM75	Uncharacterized protein	*Sorghum bicolor*	10
16	14.42	14.42	12.69	K7VQA7	Cell division control protein 48 homolog D	*Zea mays*	7
17	14.26	18.20	23.53	Q38780	11S globulin	*A. sativa*	10
18	14.22	14.22	26.11	A0A1B6P664	Glyceraldehyde-3-phosphate dehydrogenase	*Sorghum bicolor*	7
19	14.08	14.09	10.57	Q84LE9	D-Hordein	*H. vulgare*	9
20	13.44	13.46	20.39	A0A1E5VL44	Phosphoglycerate kinase	*Dichanthelium oligosanthes*	7
21	12.79	12.80	13.29	I1IA29	Uncharacterized protein	*Brachypodium distachyon*	7
22	12.00	12.00	13.57	H2F5A2	Ribulose bisphosphate carboxylase large chain	*Ophiopogon japonicus*	6
23	11.34	11.35	15.40	A0A0D9W1N0	Uncharacterized protein	*Leersia perrieri*	8
24	10.39	10.40	23.76	Q5N7P9	ATP synthase subunit beta	*O. sativa subsp. japonica*	6
25	10.10	10.10	17.51	M0XUU4	Uncharacterized protein	*H. vulgare subsp. vulgare*	5
26	10.00	10.00	7.40	I1PU83	Uncharacterized protein	*O. glaberrima*	5
27	10.00	10.00	24.10	I4EP67	Avenin	*Dichanthelium oligosanthes*	6
28	9.85	9.87	41.78	M0VFV9	Uncharacterized protein	*H. vulgare subsp. vulgare*	6
29	9.55	11.58	14.11	Q43492	Serpin-Z7	*H. vulgare*	7
30	9.36	9.38	26.07	A0A0D9X1I3	40S ribosomal protein SA	*Leersia perrieri*	5
31	9.30	9.320	10.61	W5XK42	ATP synthase subunit alpha	*Aegilops longissima*	5

32	8.76	8.77	43.36	M0ULY1	Uncharacterized protein	*H. vulgare subsp. vulgare*	7
33	8.57	10.62	25.23	P27919	Avenin	*A. sativa*	6
34	8.51	26.77	21.65	B5L808	Heat-shock protein 70	*Dactylis glomerata*	13
35	8.24	8.25	14.32	W5IA32	Formate dehydrogenase, mitochondrial	*T. aestivum*	5
36	8.24	8.25	24.24	FABPH_HUMAN			4
37	8.18	8.18	8.949	Q5EUE1	Protein disulfide-isomerase	*Zea mays*	4
38	8.10	8.10	23.66	I6TRT5	B3 hordein	*H. vulgare*	5
39	8.03	8.03	13.03	W5FJT8	Tubulin beta chain	*T. aestivum*	4
40	8.01	8.02	8.88	W5E562	Uncharacterized protein	*T. aestivum*	4
41	8.00	19.74	26.99	O49258	12s globulin	*A. sativa*	12
42	8.00	8.00	10.61	W5D5L4	Fructose-bisphosphate aldolase	*T. aestivum*	5
43	7.92	7.92	12.5	J3LEE7	Elongation factor Tu	*O. brachyantha*	4
44	7.72	7.76	31.97	G3DQF2	Ubiquitin	*Lolium perenne*	4
45	7.62	7.68	24.09	I1NPK6	GTP-binding nuclear protein	*O. glaberrima*	4
46	6.98	7.01	10.87	Q4U474	Eukaryotic initiation factor 4A	*Pennisetum americanum*	4
47	6.90	6.91	10.59	M0XD84	Malate dehydrogenase	*H. vulgare subsp. vulgare*	4
48	6.61	6.62	6.07	Q5NKP6	Starch synthase, chloroplastic/ amyloplastic	*Zea mays*	3
49	6.55	6.56	7.556	Q65C79	Tubulin alpha chain	*Setaria viridis*	3
50	6.36	6.36	71.88	F2EKE4	Predicted protein	*H. vulgare subsp. vulgare*	5
51	6.27	6.28	40.94	M0Y227	Uncharacterized protein	*H. vulgare subsp. vulgare*	6
52	6.23	6.26	12.98	C0LL37	UDP-glucose pyrophosphorylase	*Bambusa oldhamii*	4
53	6.12	6.12	6.98	A0A0D9WL26	Uncharacterized protein	*Leersia perrieri*	3
54	6.10	6.11	28.24	A0A0A9MLI5	UREG	*Arundo donax*	6
55	6.07	6.07	21.55	Q9LDC9	Os01g0265100 protein	*O. sativa subsp. japonica*	3
56	6.07	6.07	21.65	TRYP_PIG			8
57	6.06	6.06	22.28	W5FLE9	Uncharacterized protein	*T. aestivum*	3
58	6.03	6.03	16.03	K3ZJQ2|	Proteasome subunit alpha type	*Setaria italica*	3
59	6.01	6.01	34.68	F2EGD5	Predicted protein	*H. vulgare subsp. vulgare*	5
60	6.01	6.01	25.23	K7AJI8	Peptidyl-prolyl cis-trans isomerase	*Phleum pratense*	5
61	6.01	6.01	25.97	A0A1B2LQE3	Avena alpha amylase trypsin inhibitor	*Dichanthelium oligosanthes*	3
62	6.00	8.3	28.57	U6A1U2	Vromindoline VIN2	*Dichanthelium oligosanthes*	5
63	6.00	6.87	10.17	B6SHD3	Malate dehydrogenase	*Zea mays*	4
64	6.00	6.00	8.00	Q7DMU0	Storage protein	*T. aestivum*	3
65	6.00	6.00	23.49	J3M103	Uncharacterized protein	*O. brachyantha*	4
66	6.00	6.00	15.85	Q5URW0	Grain softness protein	*H. vulgare subsp. vulgare*	3

67	6.00	6.00	18.87	L0L4J7	Gliadin-like avenin	*A. sativa*		3
68	6.00	6.00	17.45	A0A1E5V4S0	Nucleoside diphosphate kinase	*Dichanthelium oligosanthes*		3
69	5.82	5.87	8.22	K3XGJ1|	Serine hydroxymethyltransferase	*Setaria italica*		3
70	5.71	5.81	34.87	Q546U1	Barley dimeric alpha-amylase inhibitor (Bdai-1)	*H. vulgare*		4
71	5.66	5.72	19.46	M8BD70	Ras-related protein RIC1	*Aegilops tauschii*		3
72	4.97	13.29	13.59	A0A1D6I6T8	Heat shock protein 90-2	*Zea mays*		10
73	4.66	4.66	6.52	Q5SNC0	Os06g0173100 protein	*O. sativa subsp. japonica*		2
74	4.64	18.76	28.53	F2E0T8	Predicted protein	*H. vulgare subsp. vulgare*		10
75	4.54	4.55	2.88	W5B7Z6	Adenosylhomocysteinase	*T. aestivum*		2
76	4.51	4.51	16.90	W0NU33	Vromindoline VIN3	*Dichanthelium oligosanthes*		2
77	4.44	4.44	21.32	A0A0A9RS32	Eno1	*Arundo donax*		3
78	4.33	4.33	13.57	Q0JDZ7	40S ribosomal protein S8	*O. sativa subsp. japonica*		2
79	4.31	4.33	8.37	C5XY44	Uncharacterized protein	*Sorghum bicolor*		3
80	4.14	4.14	4.78	TRFL_HUMAN				3
81	4.13	4.14	11.79	I1PUZ1	40S ribosomal protein S4	*O. glaberrima*		3
82	4.13	4.13	9.153	I6TEV2	Gamma 3 hordein	*H. vulgare*		4
83	4.12	8.70	33.83	I3RXT5	Glyceraldehyde-3-phosphate dehydrogenase	*Secale cereale*		4
84	4.09	4.09	14.02	PPIA_HUMAN				2
85	4.09	4.09	4.28	I1J3C6	Pyruvate kinase	*Brachypodium distachyon*		2
86	4.06	4.06	21.85	B2FH40	16.9a kDa heat-shock protein	*Dichanthelium oligosanthes*		3
87	4.05	4.06	9.75	K4APL6	Uncharacterized protein	*Setaria italica*		2
88	4.05	4.06	21.34	M0UYA9	Uncharacterized protein	*H. vulgare subsp. vulgare*		2
89	4.04	22.58	19.54	A0A0D9VSI8	Uncharacterized protein	*Leersia perrieri*		12
90	4.04	4.04	27.21	A0A1D5Z8I6	Histone H4	*T. aestivum*		3
91	4.03	4.04	17.81	F2EAF6	Predicted protein	*H. vulgare subsp. vulgare*		2
92	4.03	4.04	15.38	A0A1B2LQF1	Avena alpha amylase trypsin inhibitor	*A. sativa*		2
93	4.03	4.03	5.06	K3Y6F3	Uncharacterized protein	*Setaria italica*		3
94	4.03	4.03	14.35	W5EJ02	Ribosomal protein L19	*T. aestivum*		2
95	4.02	4.03	14.84	Q0E2I8	Os02g0235600 protein	*O. sativa subsp. japonica*		2
96	4.01	6.02	1.85	A0A0E0NCE4	Uncharacterized protein	*O. rufipogon*		4
97	4.00	16.63	24.47	J3L659	Uncharacterized protein	*O. brachyantha*		9
98	4.00	8.00	8.10	I1IMR6	Protein disulfide-isomerase	*Brachypodium distachyon*		4
99	4.00	6.00	28.66	M0VEH1	Uncharacterized protein	*H. vulgare subsp. vulgare*		3
100	4.00	4.00	3.85	Q7FAT6	OSJNBa0010H02.6 protein	*O. sativa subsp. japonica*		2

101	4.00	4.00	36.13	M0UY53	Uncharacterized protein	*H. vulgare subsp. vulgare*	3
102	4.00	4.00	5.75	F2D448	Predicted protein	*H. vulgare subsp. vulgare*	2
103	4.00	4.00	7.53	C5WYF2	Malate dehydrogenase	*Sorghum bicolor*	2
104	4.00	4.00	6.05	W5AMI8	Uncharacterized protein	*T. aestivum*	2
105	4.00	4.00	4.77	T1NLG4	Uncharacterized protein	*T. urartu*	2
106	4.00	4.00	6.66	Q3YAF9	B hordein	*H. vulgare subsp. vulgare*	2
107	4.00	4.00	3.96	A0A0D9XEA5	Uncharacterized protein	*Leersia perrieri*	2
108	4.00	4.00	2.19	A0A0D9VXV1	Lipoxygenase	*Leersia perrieri*	3
109	4.00	4.00	10.84	G1JSL4	Peroxygenase 1	*A. sativa*	2
110	4.00	4.00	6.18	W5FIP7	Uncharacterized protein	*T. aestivum*	2
111	4.00	4.00	13.62	W5C3H4	Proteasome subunit alpha type	*T. aestivum*	2
112	4.00	4.00	26.88	W4ZP51	Uncharacterized protein	*T. aestivum*	2
113	4.00	4.00	19.73	R4I506	Vromindoline 1.3	*A. sativa*	2
114	4.00	4.00	10.13	Q9SME6	Glutathione peroxidase	*H. vulgare*	2
115	4.00	4.00	12.92	Q7X9L9	QM	*T. aestivum*	2
116	4.00	4.00	5.703	M7Z059	60S acidic ribosomal protein P0	*T. urartu*	2
117	4.00	4.00	15.17	M0YS73	Uncharacterized protein	*H. vulgare subsp. vulgare*	3
118	4.00	4.00	7.43	K3XK17	Uncharacterized protein	*Setaria italica*	2
119	4.00	4.00	4.09	I1QRY4	Uncharacterized protein	*O. glaberrima*	2
120	4.00	4.00	9.76	A0A1E5VLL3	Triosephosphate isomerase, cytosolic	*Dichanthelium oligosanthes*	2
121	3.91	4.06	7.491	Q8H903	60 kDa chaperonin	*O. sativa subsp. japonica*	3
122	3.72	3.77	18.50	K3YW48	Uncharacterized protein	*Setaria italica*	2
123	3.68	9.28	20.34	M0Z2X3	Phosphoglycerate kinase	*H. vulgare subsp. vulgare*	5
124	3.46	3.53	16.47	W5FSM8	Uncharacterized protein	*T. aestivum*	2
125	3.37	3.43	18.75	K3XMZ4	Uncharacterized protein	*Setaria italica*	2
126	3.34	3.42	3.66	A0A0E0F3I1	Uncharacterized protein	*Leersia perrieri*	2
127	3.20	3.27	10.04	K3ZWD4	Proteasome subunit alpha type	*Setaria italica*	2
128	3.16	3.22	3.41	A0A0A9NCK5	Uncharacterized protein	*Arundo donax*	2
129	3.14	3.26	4.66	M7ZQM3	Globulin-1 S allele	*T. urartu*	2
130	3.02	3.08	18.25	Q0MRQ9	Late embryogenesis abundant protein	*Sorghum bicolor*	3
131	2.99	3.07	3.43	Q9AUQ4	Os03g0712700 protein	*O. sativa subsp. japonica*	2
132	2.90	2.98	18.13	K4AG37	Eukaryotic translation initiation factor 5A	*Setaria italica*	2
133	2.62	2.63	4.49	W5D7B8	60S ribosomal protein L18a	*T. aestivum*	1
134	2.60	2.61	12.31	Q84S20	CHP-rich zinc finger protein-like	*O. sativa subsp. japonica*	2
135	2.52	2.53	1.89	I1I007	Uncharacterized protein	*Brachypodium distachyon*	1

136	2.51	2.52	7.54	Q94J20	Lipoprotein-like	*O. sativa subsp. japonica*	2
137	2.47	2.48	23.35	Q7X679	OJ991214_12.15 protein	*O. sativa subsp. japonica*	2
138	2.46	2.47	5.49	W5GZB8	Uncharacterized protein	*T. aestivum*	1
139	2.39	2.40	2.31	Q6V959	Ribosomal protein L3	*T. aestivum*	1
140	2.32	2.33	6.50	W5D067	Uncharacterized protein	*T. aestivum*	1
141	2.30	2.31	20.93	J3LWV5	40S ribosomal protein S27	*O. brachyantha*	1
142	2.26	2.27	6.82	Q43238	Alcohol dehydrogenase 1	*Zea diploperennis*	2
143	2.23	2.24	12.82	Q6ZDW5	Os07g0674700 protein	*O. sativa subsp. japonica*	2
144	2.19	2.20	7.57	Q8LK80	Ferritin	*O. sativa subsp. japonica*	2
145	2.18	2.19	19.01	Q8S409	Hordoindoline A-1	*H. vulgare subsp. vulgare*	2
146	2.17	2.19	7.58	J3MNT1	Uncharacterized protein	*O. brachyantha*	1
147	2.15	2.16	1.47	W5D4Q6	Phospholipase D	*T. aestivum*	1
148	2.14	2.15	7.94	J3LXN2	Uncharacterized protein	*O. brachyantha*	1
149	2.13	2.14	6.57	Q38769	Permatin	*A. sativa*	1
150	2.12	2.13	2.25	K3YT25	Uncharacterized protein	*Setaria italica*	1
151	2.12	2.12	4.04	B6TD84	Auxin-induced protein PCNT115	*Zea mays*	1
152	2.11	2.12	8.91	A0A1E5VGM3	Annexin-like protein RJ4	*Dichanthelium oligosanthes*	3
153	2.09	2.10	11.88	W5ECL2	Uncharacterized protein	*T. aestivum*	2
154	2.09	2.10	4.326	Q5W6H1	Os05g0350500 protein	*O. sativa subsp. japonica*	1
155	2.09	2.09	13.19	W5FEZ3	40S ribosomal protein S12	*T. aestivum*	1
156	2.08	10.66	27.82	B1P766	Elongation factor 1-alpha	*Lolium temulentum*	9
157	2.08	2.09	6.14	W4ZQJ2	Uncharacterized protein	*T. aestivum*	1
158	2.07	2.08	1.354	A0A1E5WMP1	Zinc finger protein VAR3, chloroplastic	*Dichanthelium oligosanthes*	1
159	2.07	2.08	7.75	W5HZ15	Histone H2B	*T. aestivum*	1
160	2.06	2.27	10.19	W5CAA5	Uncharacterized protein	*T. aestivum*	2
161	2.05	8.68	8.72	Q5JKU5	Os01g0742200 protein	*O. sativa subsp. japonica*	5
162	2.05	6.12	30.43	A6N0B2	Mitochondrial formate dehydrogenase 1	*O. sativa subsp. indica*	4
163	2.04	2.04	18.63	F2DYL9	Predicted protein	*H. vulgare subsp. vulgare*	2
164	2.03	12.41	10.73	I1QJW3	Uncharacterized protein	*O. glaberrima*	8
165	2.03	2.11	1.81	F2EGM6	Predicted protein	*H. vulgare subsp. vulgare*	1
166	2.03	2.04	8.70	C5XN41	Uncharacterized protein	*Sorghum bicolor*	1
166	0.73	0.86	10.96	A0A0A9JUQ8	Uncharacterized protein	*Arundo donax*	1
167	2.03	2.03	5.73	W5I1R7	Uncharacterized protein	*T. aestivum*	1
168	2.03	2.03	3.86	Q25B80	Catalase	*Festuca arundinacea*	1
169	2.02	8.13	5.22	K3Y5K9	Uncharacterized protein	*Setaria italica*	4

170	2.02	2.03	8.49	Q9ZWG4	Ribulose bisphosphate carboxylase small chain	*A. sativa*	1
171	2.02	2.03	2.02	Q688K0	Guanosine nucleotide diphosphate dissociation inhibitor	*O. sativa subsp. japonica*	1
172	2.02	2.02	2.24	U5NJ12	Beta-amylase	*H. vulgare*	1
173	2.02	2.02	10.77	M8B4K5	40S ribosomal protein S15a-1	*Aegilops tauschii*	1
174	2.01	2.03	10.13	C5XE07	Uncharacterized protein	*Sorghum bicolor*	3
175	2.01	2.01	1.78	W5I3K2	Uncharacterized protein	*T. aestivum*	1
176	2.01	2.01	1.91	W5FIY5	Uncharacterized protein	*T. aestivum*	1
177	2.01	2.01	8.40	Q7X8D0	OSJNBa0035M09.3 protein	*O. sativa subsp. japonica*	1
178	2.01	2.01	9.15	Q53Q51	Os11g0151300 protein	*O. sativa subsp. japonica*	1
179	2.01	2.01	0.66	N1QUA9	Formin-like protein	*Aegilops tauschii*	1
180	2.00	14.35	13.66	K4A5Z8	Uncharacterized protein	*Setaria italica*	7
181	2.00	14.03	12.01	Q8W529	Methionine synthase	*Zea mays*	7
182	2.00	12.79	12.93	K3XEI1	Uncharacterized protein	*Setaria italica*	7
183	2.00	10.77	22.28	K3ZTW1	Uncharacterized protein	*Setaria italica*	8
184	2.00	10.2	18.99	B4FS87	Glyceraldehyde-3-phosphate dehydrogenase	*Zea mays*	5
185	2.00	10.05	8.54	K3Z491	Uncharacterized protein	*Setaria italica*	5
186	2.00	7.29	10.87	Q8HFD4	ATP synthase subunit alpha	*Dichanthelium oligosanthes*	4
187	2.00	6.00	7.78	I1GXE4	Fructose-bisphosphate aldolase	*Brachypodium distachyon*	3
188	2.00	4.07	4.57	K4A6M8	Uncharacterized protein	*Setaria italica*	2
189	2.00	4.07	4.66	J3LS33	Uncharacterized protein	*O. brachyantha*	2
190	2.00	4.06	6.07	J3LJW5	Uncharacterized protein	*O. brachyantha*	3
191	2.00	4.03	6.98	M8D1A8	Formate dehydrogenase, mitochondrial	*Aegilops tauschii*	2
192	2.00	4.00	7.40	V9QFJ0	Elongation factor-1a	*Cochliobolus miyabeanus*	2
193	2.00	4.00	2.50	I1GPA9	Lipoxygenase	*Brachypodium distachyon*	3
194	2.00	4.00	15.70	C7E3V7	Peptidyl-prolyl cis-trans isomerase	*Saccharum officinarum*	2
195	2.00	4.00	2.37	A0A0A9IRF3	Lipoxygenase	*Arundo donax*	3
196	2.00	4.00	15.87	A0A024A3B7	Low molecular weight glutenin subunit	*Pseudoroegneria spicata*	2
197	2.00	3.43	3.15	TRFE_HUMAN			2
198	2.00	2.00	2.73	I1GLH6	Uncharacterized protein	*Brachypodium distachyon*	1
199	2.00	2.00	5.14	X2JG17	Low-molecular-weight glutenin subunit Glu-B3	*T. turgidum*	1
200	2.00	2.00	3.03	W5CPE3	Uncharacterized protein	*T. aestivum*	1
201	2.00	2.00	3.36	W5BPN7	Uncharacterized protein	*T. aestivum*	1
202	2.00	2.00	4.68	I3NM41	Oleosin	*T. aestivum*	1

203	2.00	2.00	5.44	E0WBA5	Alcohol dehydrogenase	*Dichanthelium oligosanthes*	1
204	2.00	2.00	3.11	A0A0E0K6U2	S-(hydroxymethyl)glutathione dehydrogenase	*O. punctata*	1
205	2.00	2.00	4.53	W5XS41	Nad9	*T. turgidum subsp. durum*	1
206	2.00	2.00	5.73	W5I0M5	Histone H2A	*T. aestivum*	1
207	2.00	2.00	1.67	W5H440	Uncharacterized protein	*T. aestivum*	1
208	2.00	2.00	2.98	W5G105	Uncharacterized protein	*T. aestivum*	1
209	2.00	2.00	8.27	W5FVL1	Uncharacterized protein	*T. aestivum*	1
210	2.00	2.00	9.09	W5FT47	Uncharacterized protein	*T. aestivum*	1
211	2.00	2.00	9.09	W5FSX7	Non-specific lipid-transfer protein	*T. aestivum*	1
212	2.00	2.00	6.25	W5FQG6	40S ribosomal protein S26	*T. aestivum*	1
213	2.00	2.00	2.60	W5EM06	Uncharacterized protein	*T. aestivum*	1
214	2.00	2.00	2.74	W5EGF1	Uncharacterized protein	*T. aestivum*	1
215	2.00	2.00	2.66	W5DXP0	Uncharacterized protein	*T. aestivum*	1
216	2.00	2.00	2.28	W5DPV9	Uncharacterized protein	*T. aestivum*	1
217	2.00	2.00	3.61	W5D4F5	Uncharacterized protein	*T. aestivum*	1
218	2.00	2.00	3.05	W5CT81	Uncharacterized protein	*T. aestivum*	1
219	2.00	2.00	9.92	W5A3G5	Profilin	*T. aestivum*	1
220	2.00	2.00	1.89	W5A2K8	Uncharacterized protein	*T. aestivum*	1
221	2.00	2.00	6.12	W4ZWP2	Peptidyl-prolyl cis-trans isomerase	*T. aestivum*	1
222	2.00	2.00	2.04	W4ZM56	Uncharacterized protein	*T. aestivum*	1
223	2.00	2.00	3.13	T1MN05	Uncharacterized protein	*T. urartu*	1
224	2.00	2.00	6.57	T1M6E7	Xyloglucan endotransglucosylase /hydrolase	*T. urartu*	1
225	2.00	2.00	3.03	R7W9P7	Uncharacterized protein	*Aegilops tauschii*	1
226	2.00	2.00	2.34	R7W8L5	Vacuolar-sorting receptor 1	*Aegilops tauschii*	1
227	2.00	2.00	1.04	R7W7Q5	Uncharacterized protein	*Aegilops tauschii*	1
228	2.00	2.00	3.26	Q9SPD9	Voltage-dependent anion channel protein 1a	*Zea mays*	1
229	2.00	2.00	8.78	Q9SC07	Puroindoline B	*T. monococcum*	1
230	2.00	2.00	2.55	Q9LLR2	Aldehyde dehydrogenase	*O. sativa*	1
231	2.00	2.00	2.83	Q9FP24	Putative class III chitinase	*O. sativa subsp. japonica*	1
232	2.00	2.00	1.72	Q94GG8	Alanine--tRNA ligase	*O. sativa subsp. japonica*	1
233	2.00	2.00	4.34	Q8SA35	Os01g0659200 protein	*O. sativa subsp. japonica*	1
234	2.00	2.00	6.47	Q8H4T5	OSJNBa0070C17.19 protein	*O. sativa subsp. japonica*	1
235	2.00	2.00	12.70	Q8H3F5	Os07g0251301 protein	*O. sativa subsp. japonica*	1
236	2.00	2.00	13.41	Q7Y199	40S ribosomal protein S21	*O. sativa subsp. japonica*	1

237	2.00	2.00	5.77	Q6F4B1	Glutamine synthetase	*Dichanthelium oligosanthes*	1
238	2.00	2.00	9.67	Q5UNP2	Non-specific lipid-transfer protein	*H. vulgare subsp. vulgare*	1
239	2.00	2.00	5.35	Q0IR97	21 kDa protein	*O. sativa subsp. japonica*	1
240	2.00	2.00	2.73	N1R5E3	Heat shock protein STI	*Aegilops tauschii*	1
241	2.00	2.00	0.60	N1R0C0	Putative disease resistance protein RGA1	*Aegilops tauschii*	1
242	2.00	2.00	1.27	N1QVQ6	Peptidylprolyl isomerase	*Aegilops tauschii*	1
243	2.00	2.00	3.89	M8CBN8	Uncharacterized protein	*Aegilops tauschii*	1
244	2.00	2.00	1.54	M8BPX6	Uncharacterized protein	*Aegilops tauschii*	1
245	2.00	2.00	6.76	M8AVA4	Uncharacterized protein	*Aegilops tauschii*	1
246	2.00	2.00	10.64	M0Z5Y1	Uncharacterized protein	*H. vulgare subsp. vulgare*	1
247	2.00	2.00	13.27	M0UFI7	Uncharacterized protein	*H. vulgare subsp. vulgare*	1
248	2.00	2.00	1.83	K7TLU1	Hexosyltransferase	*Zea mays*	1
249	2.00	2.00	1.30	K4AMB2	Uncharacterized protein	*Setaria italica*	1
249	2.00	2.00	1.44	I1H9J6	Uncharacterized protein	*Brachypodium distachyon*	1
250	2.00	2.00	4.12	K3ZX49	Uncharacterized protein	*Setaria italica*	1
251	2.00	2.00	1.77	K3ZR22	Uncharacterized protein	*Setaria italica*	1
252	2.00	2.00	1.98	K3Z6Q4	Uncharacterized protein	*Setaria italica*	1
253	2.00	2.00	1.60	K3Y5X2	D-3-phosphoglycerate dehydrogenase	*Setaria italica*	1
254	2.00	2.00	3.91	K3XLL7	Uncharacterized protein	*Setaria italica*	1
255	2.00	2.00	5.00	K3XLG2	Uncharacterized protein	*Setaria italica*	1
256	2.00	2.00	2.46	J3KZ27	Uncharacterized protein	*O. brachyantha*	1
257	2.00	2.00	6.01	I1NTC3	Uncharacterized protein	*O. glaberrima*	1
258	2.00	2.00	3.48	I1IZP9	Uncharacterized protein	*Brachypodium distachyon*	1
259	2.00	2.00	2.08	I1IWW5	Uncharacterized protein	*Brachypodium distachyon*	1
260	2.00	2.00	3.03	I1ICU0	Uncharacterized protein	*Brachypodium distachyon*	1
261	2.00	2.00	6.97	I1HWJ6	Peptidyl-prolyl cis-trans isomerase	*Brachypodium distachyon*	1
262	2.00	2.00	3.77	I1HW94	40S ribosomal protein S3a	*Brachypodium distachyon*	1
263	2.00	2.00	1.21	I1GPF5	Uncharacterized protein	*Brachypodium distachyon*	1
264	2.00	2.00	4.28	I1GLI4	Uncharacterized protein	*Brachypodium distachyon*	1
265	2.00	2.00	8.00	F2CZ16	Predicted protein	*H. vulgare subsp. vulgare*	1
266	2.00	2.00	1.80	C5YT28	Lipoxygenase	*Sorghum bicolor*	1
267	2.00	2.00	0.382	C5YG68	Uncharacterized protein	*Sorghum bicolor*	1
268	2.00	2.00	2.20	B9FQT9	Uncharacterized protein	*O. sativa subsp. japonica*	1

269	2.00	2.00	4.32	B6STA3	IN2-1 protein	*Zea mays*	1
270	2.00	2.00	5.26	B6SSF0	Putative RING zinc finger domain superfamily protein	*Zea mays*	1
271	2.00	2.00	8.60	B1NEV4	Putative ribosomal protein S14	*O. sativa subsp. japonica*	1
272	2.00	2.00	1.91	A0A1E5VAR1	Heat shock 70 kDa protein, mitochondrial	*Dichanthelium oligosanthes*	1
273	2.00	2.00	0.88	A0A1D6M1D2	Uncharacterized protein	*Zea mays*	1
274	2.00	2.00	19.40	A0A1D5V3T5	Uncharacterized protein	*T. aestivum*	1
275	2.00	2.00	9.15	A0A1B6QL82	Uncharacterized protein	*Sorghum bicolor*	1
276	2.00	2.00	7.22	A0A1B6PNJ1	Uncharacterized protein	*Sorghum bicolor*	1
277	2.00	2.00	11.49	A0A1B2LQD4	Avena alpha amylase trypsin inhibitor-2	*A. sativa*	1
278	2.00	2.00	5.43	A0A0E0KX19	Uncharacterized protein	*O. punctata*	1
279	2.00	2.00	2.62	A0A0E0KB74	Uncharacterized protein	*O. punctata*	1
280	2.00	2.00	1.89	A0A0E0ESU9	Uncharacterized protein	*Leersia perrieri*	1
281	2.00	2.00	0.98	A0A0D9XZ63	Uncharacterized protein	*Leersia perrieri*	1
282	2.00	2.00	3.49	A0A0D9V729	Uncharacterized protein	*Leersia perrieri*	1
284	2.00	2.00	10.64	A0A0A9KFS9	Acyl carrier protein	*Arundo donax*	1
285	2.00	2.00	8.14	A0A0A9HKZ3	LOX4	*Arundo donax*	1
286	2.00	2.00	3.68	A0A0A9FJS6	CASP-like protein	*Arundo donax*	1
287	2.00	2.00	4.97	RETBP_HUMAN			1
288	2.00	2.00	6.84	HBB_HUMAN			1
289	2.00	2.00	4.70	ANXA5_HUMAN			1
290	1.96	2.00	4.95	C5X3B9	Uncharacterized protein	*Sorghum bicolor*	1
291	1.92	2.00	18.4	W5E8X2	Uncharacterized protein	*T. aestivum*	1
292	1.92	2.00	1.59	A0A1D5S1L8	Uncharacterized protein	*T. aestivum*	1
293	1.92	2.00	4.76	A0A0D3HE95	Uncharacterized protein	*Leersia perrieri*	1
294	1.89	2.00	1.80	A0A1D6H9V7	Poly [ADP-ribose] polymerase	*Zea mays*	1
295	1.87	6.24	3.21	Q9MB32	Heat shock protein 90	*O. sativa*	4
296	1.85	2.00	3.35	Q94JJ0	Fructose-bisphosphate aldolase	*O. sativa subsp. japonica*	1
297	1.85	2.00	4.06	Q2RAW0	60S acidic ribosomal protein P0	*O. sativa subsp. japonica*	2
298	1.82	8.03	12.98	W5D048	Tubulin beta chain	*T. aestivum*	4
299	1.78	1.93	1.71	J3LDG6	Poly [ADP-ribose] polymerase	*O. brachyantha*	1
300	1.77	1.92	7.20	J3MZ72	Proteasome subunit beta	*O. brachyantha*	1
301	1.74	1.99	10.79	C5YU07	Uncharacterized protein	*Sorghum bicolor*	2
302	1.66	1.91	10.62	I1HYI5	Uncharacterized protein	*Brachypodium distachyon*	2
303	1.62	1.88	3.33	T1NSH3	Glyceraldehyde-3-phosphate dehydrogenase	*T. urartu*	1
304	1.62	1.77	2.76	Q8S4Y9	Glyceraldehyde-3-phosphate dehydrogenase	*O. sativa*	1
305	1.60	1.86	5.94	W5HK74	Uncharacterized protein	*T. aestivum*	2

306	1.57	1.72	12.50	Q01L74	H0321H01.6 protein	*O. sativa*	1
307	1.46	1.60	8.84	Q5URW7	Hordoindoline b-2	*H. vulgare subsp. vulgare*	1
308	1.37	1.51	5.08	M7ZE30	Beta-amylase	*T. urartu*	1
309	1.36	1.49	3.61	Q9LST5	Proteasome subunit beta type	*O. sativa subsp. japonica*	1
310	1.35	1.48	6.50	Q5VPF1	Proteasome subunit beta type	*O. sativa subsp. japonica*	1
311	1.35	1.48	1.22	A0A0D9V8I3	Uncharacterized protein	*Leersia perrieri*	1
312	1.30	1.45	3.6	A0A0D9X1M2	40S ribosomal protein S6	*Leersia perrieri*	1
313	1.29	3.51	9.75	I1IPR0	Uncharacterized protein	*Brachypodium distachyon*	2
314	1.23	1.38	3.05	W5FGX7	Uncharacterized protein	*T. aestivum*	1
315	1.18	1.33	2.67	R7WE15	Transaldolase 1	*Aegilops tauschii*	1
316	1.15	1.30	3.67	A0A1D5VZV3	Uncharacterized protein	*T. aestivum*	1
317	1.14	1.28	4.22	Q5Z816	Os06g0703600 protein	*O. sativa subsp. japonica*	1
318	1.14	1.28	5.74	M0V337	REVERSED Uncharacterized protein	*H. vulgare subsp. vulgare*	1
319	1.11	1.25	2.50	F2EG51	Predicted protein	*H. vulgare subsp. vulgare*	1
320	1.08	1.22	5.07	R7W067	60S ribosomal protein L23a	*Aegilops tauschii*	1
321	1.07	1.21	5.16	A0A1D6JQU8	Uncharacterized protein	*Zea mays*	1
322	1.04	1.17	3.96	W5DQS5	Uncharacterized protein	*T. aestivum*	1
323	1.03	1.17	9.32	N1QV84	Regulator of ribonuclease-like protein 2	*Aegilops tauschii*	1
324	1.03	1.16	1.05	I1Q7V6	REVERSED Uncharacterized protein	*O. glaberrima*	1
325	1.02	16.22	19.24	Q9M7E4	Elongation factor 1 alpha	*Zea mays*	10
326	0.99	12.88	20.92	A0A1E5UZA4	Phosphoglycerate kinase	*Dichanthelium oligosanthes*	7
327	0.98	1.12	10.67	K3ZER9	Uncharacterized protein	*Setaria italica*	1
328	0.95	1.13	4.60	I1H6J2	Uncharacterized protein	*Brachypodium distachyon*	2
329	0.93	1.07	20.83	A0A067YHZ8	Glyceraldehyde-3-phosphate DH	*H. vulgare subsp. spontaneum*	1
330	0.9	1.03	3.15	K4AB95	Uncharacterized protein	*Setaria italica*	1
331	0.87	1.00	1.16	J3MLW3	Uncharacterized protein	*O. brachyantha*	1
332	0.86	1.00	1.61	Q10LY9	2,3-bisphosphoglycerate-independent phosphoglycerate mutase, putative	*O. sativa subsp. japonica*	1
333	0.77	0.90	14.78	A0A0P0Y6A6	Non-specific lipid-transfer protein	*O. sativa subsp. japonica*	1
334	0.76	0.89	3.21	K3Z766	Uncharacterized protein	*Setaria italica*	1
335	0.75	3.77	7.17	A0A0Q3KAB9	Uncharacterized protein	*Brachypodium distachyon*	2
336	0.75	0.88	2.24	W5GV92	Uncharacterized protein	*T. aestivum*	1

Supplementary Table 4. Protein identifications in powdered drinks (PD). Trypsin digestion peptides were identified after database searching against the Poaceae subset of the Uniprot database appended with the Common Repository of Adventitious Protein (cRAP) database using a 1% global false discovery rate (FDR) threshold.

N	Unused Score	Total Score	% Cov (95%)	Accession	Name	Species	Peptides (95%)
1	57.18	57.18	64.74	CASK_BOVIN	Casein	*Bos taurus*	138
2	46.48	46.48	44.32	ALBU_BOVIN	Albumin	*Bos taurus*	24
3	45.44	45.46	75.70	CAS1_BOVIN	Casein	*Bos taurus*	199
4	44.41	44.89	66.90	LALBA_BOVIN	Lactalbumin	*Bos taurus*	82
5	44.11	45.33	62.61	CAS2_BOVIN	Casein	*Bos taurus*	96
6	41.94	41.94	38.44	K7X1I9	Alpha-gliadin	*T. timopheevii subsp. araraticumcum aestivum*	28
7	28.72	28.72	49.00	M0UEE6	Uncharacterized protein	*H. vulgare subsp. vulgare*	20
8	28.63	28.66	58.04	CASB_BOVIN	Casein	*Bos taurus*	73
9	26.34	26.34	16.47	I6SW34	D hordein	*H. vulgare subsp. vulgare*	42
10	25.08	25.13	35.04	A0A1D5YFA7	Beta-amylase	*T. timopheevii subsp. araraticumcum aestivum*	19
11	24.12	24.13	77.78	M0V3U0	Non-specific lipid-transfer protein	*H. vulgare subsp. vulgare*	98
12	22.04	22.04	67.13	M0ULY1	Uncharacterized protein	*H. vulgare subsp. vulgare*	31
13	21.36	21.45	19.13	K2C1_HUMAN	Keratin	*Homo sapiens*	13
14	18.30	18.30	64.33	M0Y227	Uncharacterized protein	*H. vulgare subsp. vulgare*	45
15	18.06	18.07	50.00	M0VFV9	Uncharacterized protein	*H. vulgare subsp. vulgare*	20
16	17.01	17.07	37.99	I6TRT5	B3 hordein	*H. vulgare*	26
17	16.60	18.77	48.08	M0Z714	Uncharacterized protein	*H. vulgare subsp. vulgare*	16
18	16.41	16.43	65.13	Q546U1	Barley dimeric alpha-amylase inhibitor (Bdai-1)	*H. vulgare*	19
19	15.99	16.12	56.95	X2KYP9	Monomeric alpha-amylase inhibitor	*T. timopheevii subsp. araraticumcum aestivum*	12
20	15.03	18.25	63.69	Q6S5B1	Alpha amylase inhibitor CM3	*T. timopheevii subsp. araraticumcum turgidum subsp. durum*	30
21	12.71	15.48	13.85	A0A060MZP1	High molecular weight glutenin subunit	*T. timopheevii subsp. araraticumcum aestivum*	14
22	12.49	12.49	44.78	A0A1D6CWE1	Uncharacterized protein	*T. timopheevii subsp. araraticumcum aestivum*	23
23	12.46	12.48	65.25	A0A1D5SYE4	Uncharacterized protein	*T. timopheevii subsp. araraticumcum aestivum*	8
24	12.32	12.40	29.83	I6TEV2	Gamma 3 hordein	*H. vulgare*	35
25	12.31	12.33	59.57	C8CAI4	Dimeric alpha-amylase inhibitor	*T. timopheevii subsp. araraticumcum aestivum*	13

26	12.17	12.38	11.64	K1C10_HUMAN			6
27	11.94	12.03	10.83	Q7DMU0	Storage protein	*T. timopheevii subsp. araraticumcum aestivum*	7
28	11.58	11.76	73.10	M0YS73	Uncharacterized protein	*H. vulgare subsp. vulgare*	18
29	11.47	11.55	28.62	B6DQD5	Gamma-gliadin	*Secale cereale x T. timopheevii subsp. araraticumcum aestivum*	12
30	11.44	11.50	44.22	Q5URW6	Hordoindoline b-1	*H. vulgare subsp. vulgare*	9
31	10.48	10.51	71.88	F2EKE4	Predicted protein	*H. vulgare subsp. vulgare*	16
32	10.27	10.52	34.45	Q8W3W7	Low-molecular-weight glutenin subunit group 3 type II	*T. timopheevii subsp. araraticumcum aestivum*	24
33	9.68	22.58	33.96	J7HWD7	Alpha-gliadin	*T. timopheevii subsp. araraticumcum aestivum*	16
34	9.62	16.00	60.14	Q41540	CM 17 protein	*T. timopheevii subsp. araraticumcum aestivum*	20
35	9.42	19.37	22.80	U5NIV6	Beta-amylase	*H. vulgare subsp. spontaneum*	20
36	9.36	9.44	35.37	M0UYA9	Uncharacterized protein	*H. vulgare subsp. vulgare*	11
37	9.15	9.25	27.56	Q0Q5D9	Globulin 1	*T. timopheevii subsp. araraticumcum aestivum*	5
38	9.02	23.91	31.62	I0IT55	Alpha/beta-gliadin	*T. timopheevii subsp. araraticumcum aestivum*	16
39	8.97	9.02	25.00	W8PU74	Farinin protein	*Brachypodium distachyon*	8
40	8.45	8.52	8.18	A5HMG1	HMW glutenin subunit 1Bx13	*T. timopheevii subsp. araraticumcum aestivum*	7
41	8.40	8.51	51.03	P16851	Alpha-amylase/trypsin inhibitor CM2	*T. timopheevii subsp. araraticumcum aestivum*	10
42	8.36	8.38	24.83	W8E2L7	Gamma-gliadin	*Aegilops tauschii*	12
43	8.11	8.15	24.09	R9XV91	LMW-GS	*T. timopheevii subsp. araraticumcum aestivum*	11
44	8.07	8.07	25.11	TRYP_PIG			28
45	8.01	20.56	28.47	A0A1P8DST0	Alpha-gliadin storage protein	*Secale cereale x T. timopheevii subsp. araraticumcum aestivum*	14
46	8.00	12.30	60.99	C3VWA4	Dimeric alpha-amylase inhibitor	*T. timopheevii subsp. araraticumcum dicoccoides*	14
47	8.00	8.92	10.04	A0A0K0KDM6	High molecular weight glutenin subunit 1Dy3	*T. timopheevii subsp. araraticumcum aestivum*	8
48	8.00	8.00	27.09	A0A1D6DC72	Uncharacterized protein	*T. timopheevii subsp. araraticumcum aestivum*	7
49	7.83	7.96	73.49	M0W9B7	Uncharacterized protein	*H. vulgare subsp. vulgare*	12

50	7.74	10.07	22.86	B2BZD2	LMW-m glutenin subunit 0877L13-M	*T. timopheevii subsp. araraticumcum aestivum*	15
51	7.71	7.82	37.75	M8C5C1	Uncharacterized protein	*Aegilops tauschii*	5
52	7.62	7.71	20.07	A0A173DQZ7	Type-b avenin-like protein	*T. timopheevii subsp. araraticumcum aestivum*	7
53	7.60	7.73	8.50	A0A1D6APZ0	Uncharacterized protein	*T. timopheevii subsp. araraticumcum aestivum*	4
54	7.59	7.66	21.35	I3NM41	Oleosin	*T. timopheevii subsp. araraticumcum aestivum*	6
55	7.54	7.62	28.77	M8A1S2	Trypsin/alpha-amylase inhibitor CMX1/CMX3	*T. timopheevii subsp. araraticumcum urartu*	4
56	7.24	7.44	46.34	N1R4E0	Uncharacterized protein	*Aegilops tauschii*	4
57	7.14	9.99	51.03	R7W9W1	Alpha-amylase/trypsin inhibitor CM1	*Aegilops tauschii*	11
58	7.14	7.41	8.67	K1C9_HUMAN			5
59	7.00	7.08	7.41	F2EBM4	Predicted protein	*H. vulgare subsp. vulgare*	5
60	6.96	7.07	9.22	M0XUU4	Uncharacterized protein	*H. vulgare subsp. vulgare*	4
61	6.93	9.84	11.81	Q94IJ6	High molecular weight glutenin subunit y	*T. timopheevii subsp. araraticumcum aestivum*	11
62	6.59	6.64	24.39	G8CLS3	Grain softness protein	*Taeniatherum caput-medusae*	5
63	6.47	6.51	34.90	M0UY52	Uncharacterized protein	*H. vulgare subsp. vulgare*	14
64	6.39	6.42	16.18	Q53WS1	Alpha 1 purothionin	*T. timopheevii subsp. araraticumcum aestivum*	5
65	6.02	8.46	43.90	M0VEH1	Uncharacterized protein	*H. vulgare subsp. vulgare*	19
66	6.00	19.92	67.61	B9VRI3	Alpha-amylase inhibitor CM16 subunit	*Secale cereale x T. timopheevii subsp. araraticumcum aestivum*	24
67	6.00	12.47	67.74	M0VEJ0	Uncharacterized protein	*H. vulgare subsp. vulgare*	10
68	5.87	6.07	15.94	M0ZDL8	Uncharacterized protein	*H. vulgare subsp. vulgare*	4
69	5.75	5.91	10.06	A0A0D9V992	Fructose-bisphosphate aldolase	*Leersia perrieri*	3
70	5.70	5.94	27.01	M0V715	Uncharacterized protein	*H. vulgare subsp. vulgare*	6
71	5.69	5.82	9.74	M8BDX3	Uncharacterized protein	*Aegilops tauschii*	4
72	5.52	5.57	31.51	F2EAF6	Predicted protein	*H. vulgare subsp. vulgare*	4
73	5.18	7.66	17.29	W5FZ62	Uncharacterized protein	*T. timopheevii subsp. araraticumcum aestivum*	6
74	4.99	5.05	27.38	A8V4A4	Chymotrypsin inhibitor-2	*H. vulgare subsp. vulgare*	3
75	4.77	4.82	32.71	F2EEH7	Predicted protein	*H. vulgare subsp. vulgare*	4
76	4.66	6.78	18.75	Q84NG7	Globulin	*H. vulgare*	5

77	4.66	4.67	6.24	F2D284	Protein disulfide-isomerase	*H. vulgare subsp. vulgare*	4
78	4.48	5.32	24.56	Q1ZZT4	Low-molecular-weight glutenin subunit	*T. timopheevii subsp. araraticumcum aestivum*	10
79	4.46	4.48	36.00	Q5ITV1	Hordoindoline-a	*H. vulgare subsp. vulgare*	6
80	4.31	8.51	25.83	I6R4A7	B-hordein	*H. vulgare subsp. vulgare*	11
81	4.31	4.32	17.05	I6TMV6	Gamma 1 hordein	*H. vulgare*	7
82	4.29	4.31	9.86	M0Z4S0	Uncharacterized protein	*H. vulgare subsp. vulgare*	4
83	4.27	6.32	18.47	A0A1D5X2J6	Uncharacterized protein	*T. timopheevii subsp. araraticumcum aestivum*	3
84	4.22	17.22	28.15	A0A0E3URD2	Alpha-gliadin	*Secale cereale x T. timopheevii subsp. araraticumcum aestivum*	14
85	4.19	8.71	33.72	D2KFH1	Avenin-like a4	*T. timopheevii subsp. araraticumcum aestivum*	18
86	4.19	4.20	10.22	W5FN32	Uncharacterized protein	*T. timopheevii subsp. araraticumcum aestivum*	2
87	4.15	9.21	21.88	B2BZC7	LMW-m glutenin subunit 0154A5-M	*T. timopheevii subsp. araraticumcum aestivum*	12
88	4.12	4.80	44.79	Q9FEK9	Lipid transfer protein	*T. timopheevii subsp. araraticumcum turgidum subsp. durum*	7
89	4.09	4.10	6.91	N1R3X3	Peroxidase	*Aegilops tauschii*	2
90	4.06	4.07	17.39	F2EJ79|	Predicted protein	*H. vulgare subsp. vulgare*	2
91	4.05	4.05	15.24	Q5URW0	Grain softness protein	*H. vulgare subsp. vulgare*	6
92	4.03	4.15	48.44	Q8S9N0	Ubiquitin	*O. sativa*	3
93	4.02	6.79	27.63	H6VLQ4	LMW-GS	*T. timopheevii subsp. araraticumcum aestivum*	19
94	4.02	4.02	12.43	I1PWZ3	Uncharacterized protein	*O. glaberrima*	2
95	4.01	4.01	18.55	Q5UNP2	Non-specific lipid-transfer protein	*H. vulgare subsp. vulgare*	2
96	4.00	15.28	26.59	N1NV64	Beta-amylase	*Brachypodium distachyon*	12
97	4.00	14.63	8.96	V5RM88	D-Hordein	*Secale cereale x T. timopheevii subsp. araraticumcum aestivum*	18
98	4.00	12.30	55.84	W5D003	Uncharacterized protein	*T. timopheevii subsp. araraticumcum aestivum*	12
99	4.00	10.02	44.22	G1UH43	Hordoindoline b-2	*H. vulgare subsp. vulgare*	10
100	4.00	7.71	21.83	V5M3L6	Avenin-like protein	*T. timopheevii subsp. araraticumcum aestivum*	5
101	4.00	4.00	23.18	W5AGK9	Nucleoside diphosphate kinase	*T. timopheevii subsp. araraticumcum aestivum*	3
102	4.00	4.00	6.64	W5BUF4	Caleosin	*T. timopheevii subsp. araraticumcum aestivum*	3

103	4.00	4.00	25.95	Q38770	Type V Thionin	*Aegilops tauschii*	3
104	4.00	4.00	3.65	W5DYF8	Uncharacterized protein	*T. timopheevii subsp. araraticumcum aestivum*	2
105	4.00	4.00	13.29	Q43770	Oleosin	*H. vulgare*	2
106	4.00	4.00	7.09	M0XIN0	Uncharacterized protein	*H. vulgare subsp. vulgare*	2
107	4.00	4.00	19.43	F2CR90	Predicted protein	*H. vulgare subsp. vulgare*	2
108	3.93	4.07	25.86	M8BYH8	Non-specific lipid-transfer protein	*Aegilops tauschii*	5
109	3.81	3.92	9.26	W5EQ17	Uncharacterized protein	*T. timopheevii subsp. araraticumcum aestivum*	2
110	3.74	3.82	14.37	F2E8X4	Oleosin	*H. vulgare subsp. vulgare*	3
111	3.61	3.73	26.80	M0VJA1	Uncharacterized protein	*H. vulgare subsp. vulgare*	3
112	3.53	3.63	17.11	F2EC88	Predicted protein	*H. vulgare subsp. vulgare*	3
113	3.49	3.63	12.92	L7VFZ3	Delta gliadin 1	*Aegilops tauschii*	6
114	3.44	3.54	11.66	F2EL01	Predicted protein	*H. vulgare subsp. vulgare*	2
115	3.39	7.43	69.88	M0XBS5	Uncharacterized protein	*H. vulgare subsp. vulgare*	7
116	3.39	3.48	17.28	T1N474	Uncharacterized protein	*T. timopheevii subsp. araraticumcum urartu*	2
117	3.38	3.48	20.69	P07596	Alpha-amylase/subtilisin inhibitor	*H. vulgare*	3
118	3.33	3.46	12.31	I4EP89	Avenin	*Secale cereale x T. timopheevii subsp. araraticumcum aestivum*	2
119	3.28	3.37	22.15	W5EIR1	Uncharacterized protein	*T. timopheevii subsp. araraticumcum aestivum*	3
120	3.27	3.49	7.19	Q42837	Aldose reductase	*H. vulgare*	2
121	3.16	19.58	30.73	Q43492	Serpin-Z7	*H. vulgare*	15
122	3.11	3.32	20.66	Q9SES6	Non-specific lipid-transfer protein	*H. vulgare*	2
123	3.09	35.91	33.11	A0A1P8DTD6	Alpha-gliadin storage protein	*Secale cereale x T. timopheevii subsp. araraticumcum aestivum*	25
124	3.04	3.12	3.92	W5ANJ9	Uncharacterized protein	*T. timopheevii subsp. araraticumcum aestivum*	2
125	3.00	3.14	13.25	R7W9M2	Putative aquaporin TIP3-1	*Aegilops tauschii*	3
126	2.98	3.08	6.63	F2ECH4	Predicted protein	*H. vulgare subsp. vulgare*	2
127	2.87	3.62	46.32	M8BVH7	Putative non-specific lipid-transfer protein	*Aegilops tauschii*	5
128	2.83	2.91	17.39	A0A1D6ACI6	Uncharacterized protein	*T. timopheevii subsp. araraticumcum aestivum*	2
129	2.81	2.89	10.47	B5TWK6	rRNA N-glycosidase	*H. vulgare*	2
130	2.78	2.85	20.98	N1QTW5	Trypsin inhibitor CMc	*Aegilops tauschii*	5

131	2.71	5.30	32.95	M0VH55	Uncharacterized protein	*H. vulgare subsp. vulgare*	20
132	2.68	2.77	6.05	W5FPN2	Uncharacterized protein	*T. timopheevii subsp. araraticumcum aestivum*	2
133	2.62	2.81	26.32	D0UXW7	Superoxide dismutase	*Bambusa oldhamii*	4
134	2.60	2.83	16.98	B5TWD1	Late embryogenesis abundant protein	*H. vulgare subsp. vulgare*	4
135	2.52	2.66	10.26	A0A1D5UB33	Uncharacterized protein	*T. timopheevii subsp. araraticumcum aestivum*	2
136	2.46	6.22	17.26	Q8LKV8	Seed globulin	*Aegilops tauschii*	3
137	2.46	2.51	2.86	F2DS64	Predicted protein	*H. vulgare subsp. vulgare*	1
138	2.41	6.14	24.83	M8BAK8	Uncharacterized protein	*Aegilops tauschii*	3
139	2.38	2.43	2.16	W5XK42	ATP synthase subunit alpha	*Aegilops longissima*	1
140	2.36	21.60	29.76	K7X0N8	Alpha-gliadin	*T. timopheevii subsp. araraticumcum aestivum*	19
141	2.36	2.41	8.20	Q42848	Non-specific lipid-transfer protein	*H. vulgare*	1
142	2.34	2.39	9.51	F2CSK4	Predicted protein	*H. vulgare subsp. vulgare*	3
143	2.29	2.34	18.98	Q4G3S6	B3-hordein	*Hordeum chilense*	6
144	2.29	2.33	2.02	Q8HRM3	Ribulose bisphosphate carboxylase large chain	*Hordeum chilenseDasypogon hookeri*	1
145	2.28	5.32	6.14	A0A1D5Z1A1	Uncharacterized protein	*T. timopheevii subsp. araraticumcum aestivum*	3
146	2.26	2.42	2.84	A0A1E5VRS5	Luminal-binding protein 5	*Secale cereale x T. timopheevii subsp. araraticumcum aestivum*	2
147	2.23	4.36	23.59	R4JDK8	Low-molecular-weight glutenin subunit	*T. timopheevii subsp. araraticumcum aestivum*	7
148	2.23	2.27	23.60	M0W4X5	Uncharacterized protein	*H. vulgare subsp. vulgare*	2
149	2.23	2.27	5.03	F2DXR4	Predicted protein	*H. vulgare subsp. vulgare*	1
150	2.21	2.36	19.00	F2DB07	Predicted protein	*H. vulgare subsp. vulgare*	2
151	2.18	2.22	4.15	I1PMF5	Glyceraldehyde-3-phosphate dehydrogenase	*O. glaberrima*	1
152	2.17	2.21	7.65	D2KFH0	Gliadin/avenin-like seed protein	*T. timopheevii subsp. araraticumcum aestivum*	1
153	2.16	8.73	37.71	P0CZ08	Avenin-like a3	*T. timopheevii subsp. araraticumcum aestivum*	20
154	2.15	2.18	5.17	M0YZF2	Uncharacterized protein	*H. vulgare subsp. vulgare*	2
155	2.14	2.17	5.36	W5AUU7	Uncharacterized protein	*T. timopheevii subsp. araraticumcum aestivum*	1
156	2.13	2.16	2.70	W5I9I6	Uncharacterized protein	*T. timopheevii subsp. araraticumcum aestivum*	1

157	2.08	10.30	8.54	A0A142ESP3	High molecular weight glutenin subunit 1Bx protein	*T. timopheevii subsp. araraticumcum dicoccoides*	13
158	2.08	2.12	14.80	B8XU28	Alfa gliadin	*T. timopheevii subsp. araraticumcum monococcum*	4
159	2.07	2.09	2.46	U5S8M6	Elongation factor 1-alpha	*Dendrocalamus latiflorus*	1
160	2.07	2.08	6.54	P93602	PUP88 protein; member of trypsin/a-amylase inhibitors family from cereals	*T. timopheevii subsp. araraticumcum aestivum*	1
161	2.06	2.07	29.87	Q84L51	Metallothionein-like protein type 4	*H. vulgare subsp. vulgare*	1
162	2.04	4.73	33.33	Q2PCC7	Type 2 non specific lipid transfer protein	*T. timopheevii subsp. araraticumcum aestivum*	5
163	2.04	2.06	11.30	M8ADF8	Uncharacterized protein	*T. timopheevii subsp. araraticumcum urartu*	2
164	2.04	2.05	4.76	M7Z267	Uncharacterized protein	*T. timopheevii subsp. araraticumcum urartu*	1
165	2.04	2.04	5.51	I1HT86	Uncharacterized protein	*Brachypodium distachyon*	1
166	2.03	2.04	4.94	Q9T2L5	26 kDa heat shock protein	*H. vulgare*	2
167	2.02	6.05	23.78	Q9FVJ5	GSP-A1	*T. timopheevii subsp. araraticumcum aestivum*	4
168	2.02	2.02	6.02	M0UGW6	Uncharacterized protein	*H. vulgare subsp. vulgare*	1
169	2.01	8.05	22.89	H8Y0H9	Gamma prolamin	*Elymus elongatus*	12
170	2.00	32.68	28.52	A5JTR3	Alpha-gliadin Gli-Ts4	*Secale cereale x T. timopheevii subsp. araraticumcum aestivum*	20
171	2.00	29.89	27.54	R9XSW3	Alpha-gliadin	*T. timopheevii subsp. araraticumcum aestivum*	18
172	2.00	14.96	50.33	C4P5J4	Monomeric alpha-amylase inhibitor	*T. timopheevii subsp. araraticumcum dicoccoides*	10
173	2.00	14.07	56.78	Q45FA6	Cereal-type amylase inhibitor	*Secale cereale*	23
174	2.00	13.03	27.94	Q6QGV8	LMW glutenin pGM107	*T. timopheevii subsp. araraticumcum aestivum*	31
175	2.00	10.60	27.99	P06470	B1-hordein	*H. vulgare*	16
176	2.00	10.51	69.00	M0WPC3	Uncharacterized protein	*H. vulgare subsp. vulgare*	17
177	2.00	8.57	20.27	R4JBK0	Low-molecular-weight glutenin subunit	*T. timopheevii subsp. araraticumcum aestivum*	14
178	2.00	8.43	43.93	M8BZV7	Uncharacterized protein	*Aegilops tauschii*	18
179	2.00	6.07	12.50	A0A089XB95	Low-molecular-weight glutenin subunit	*T. timopheevii subsp. araraticumcum urartu*	5
180	2.00	6.03	19.92	H8Y0P9	Gamma prolamin	*Secale cereale x T. timopheevii subsp. araraticumcum aestivum*	13

181	2.00	6.00	23.50	M8ANS4	Avenin-3	*T. timopheevii subsp. ararаticumcum urartu*	5
182	2.00	5.51	8.31	W5I5X1	Uncharacterized protein	*T. timopheevii subsp. araraticumcum aestivum*	4
183	2.00	5.51	11.36	W5HSW8	Uncharacterized protein	*T. timopheevii subsp. araraticumcum aestivum*	4
184	2.00	4.28	31.69	Q8S409	Hordoindoline A-1	*H. vulgare subsp. vulgare*	5
185	2.00	4.00	23.33	Q9LKM0	Nucleoside diphosphate kinase	*Lolium perenne*	3
186	2.00	3.37	22.15	P04464	Calmodulin	*T. timopheevii subsp. araraticumcum aestivum*	3
187	2.00	2.16	16.34	A0A1B2LQF0	Avena alpha amylase trypsin inhibitor	*Secale cereale x T. timopheevii subsp. araraticumcum aestivum*	2
188	2.00	2.00	19.19	M0VFD9	Uncharacterized protein	*H. vulgare subsp. vulgare*	1
189	2.00	2.00	4.74	W5DIU6	Uncharacterized protein	*T. timopheevii subsp. araraticumcum aestivum*	1
190	2.00	2.00	7.98	Q9MB31	GSH-dependent dehydroascorbate reductase 1	*O. sativa subsp. japonica*	1
191	2.00	2.00	8.67	M0VDB7	Uncharacterized protein	*H. vulgare subsp. vulgare*	1
192	2.00	2.00	2.15	W8FJ68	Starch synthase, chloroplastic/ amyloplastic	*T. timopheevii subsp. araraticumcum aestivum*	1
193	2.00	2.00	2.98	W5G105	Uncharacterized protein	*T. timopheevii subsp. araraticumcum aestivum*	1
194	2.00	2.00	3.97	W5DQS5	Uncharacterized protein	*T. timopheevii subsp. araraticumcum aestivum*	1
195	2.00	2.00	8.41	W5DDD6	Uncharacterized protein	*T. timopheevii subsp. araraticumcum aestivum*	1
196	2.00	2.00	3.53	W5BGU3	Uncharacterized protein	*T. timopheevii subsp. araraticumcum aestivum*	2
197	2.00	2.00	9.71	W5B8I7	Histone H4	*T. timopheevii subsp. araraticumcum aestivum*	1
198	2.00	2.00	15.91	W4ZN48	Uncharacterized protein	*T. timopheevii subsp. araraticumcum aestivum*	1
199	2.00	2.00	6.57	R7WDG6	40S ribosomal protein S14	*Aegilops tauschii*	1
200	2.00	2.00	12.50	R7W139	Uncharacterized protein	*Aegilops tauschii*	1
201	2.00	2.00	2.24	Q9ZWH7	Catalase	*O. sativa*	1
202	2.00	2.00	2.84	Q9FP24	Putative class III chitinase	*O. sativa subsp. japonica*	2
203	2.00	2.00	6.43	Q946Z1	Thaumatin-like protein TLP4	*H. vulgare*	1
204	2.00	2.00	7.22	Q8H4B9	Os07g0516200 protein	*O. sativa subsp. japonica*	1
205	2.00	2.00	6.07	Q84QT6	Putative uncharacterized protein	*O. sativa subsp. japonica*	1
206	2.00	2.00	8.08	Q6ZG29	Globulin-1S-like	*O. sativa subsp. japonica*	1

207	2.00	2.00	1.83	Q655Y7	Hydroxyproline-rich glycoprotein-like	*O. sativa subsp. japonica*	1
208	2.00	2.00	7.23	Q2TN84	USP family protein	*T. timopheevii subsp. araraticumcum aestivum*	1
209	2.00	2.00	5.36	Q0IR97	21 kDa protein	*O. sativa subsp. japonica*	1
210	2.00	2.00	5.77	M0XN12	Uncharacterized protein	*H. vulgare subsp. vulgare*	1
211	2.00	2.00	5.80	M0WAL0	Uncharacterized protein	*H. vulgare subsp. vulgare*	1
212	2.00	2.00	4.32	M0VF30	Uncharacterized protein	*H. vulgare subsp. vulgare*	1
213	2.00	2.00	3.44	M0UUA7	Uncharacterized protein	*H. vulgare subsp. vulgare*	1
214	2.00	2.00	2.82	K7VF10	Coiled-coil domain-containing protein 97 isoform 1	*Zea mays*	1
215	2.00	2.00	1.28	K3ZQM9	Uncharacterized protein	*Setaria italica*	1
216	2.00	2.00	7.79	F2DD34	Predicted protein	*H. vulgare subsp. vulgare*	1
217	2.00	2.00	9.15	F2D791	Predicted protein	*H. vulgare subsp. vulgare*	1
218	2.00	2.00	9.62	E7BB46	Barley trypsin inhibitor CMc	*H. vulgare subsp. spontaneum*	2
219	2.00	2.00	5.04	A0A1D6SCQ9	Uncharacterized protein	*T. timopheevii subsp. araraticumcum aestivum*	1
220	2.00	2.00	7.96	A0A1D6G2X1	S-adenosyl-L-methionine-dependent methyltransferase superfamily protein	*Zea mays*	1
221	2.00	2.00	0.90	A0A1D6DFF6	Uncharacterized protein	*T. timopheevii subsp. araraticumcum aestivum*	1
222	2.00	2.00	1.38	A0A1D5VH79	Uncharacterized protein	*T. timopheevii subsp. araraticumcum aestivum*	1
223	2.00	2.00	1.60	A0A1D5S1L8	Uncharacterized protein	*T. timopheevii subsp. araraticumcum aestivum*	1
224	2.00	2.00	2.76	A0A0E0LHV7	Transmembrane 9 superfamily member	*O. punctata*	1
225	2.00	2.00	5.44	A0A0E0KX19	Uncharacterized protein	*O. punctata*	1
226	2.00	2.00	0.63	A0A0E0EB48	Uncharacterized protein	*Leersia perrieri*	1
227	2.00	2.00	1.67	Q0ITE1	REVERSED Os11g0265600 protein	*O. sativa subsp. japonica*	1
228	1.96	2.00	6.22	M8AZZ5	Bowman-Birk type trypsin inhibitor	*Aegilops tauschii*	1
229	1.96	2.00	6.90	M0WW64	Uncharacterized protein	*H. vulgare subsp. vulgare*	1
230	1.89	2.00	4.10	K3YU83	Uncharacterized protein	*Setaria italica*	1
231	1.88	18.18	59.28	M8BV45	Alpha-amylase/trypsin inhibitor CM3	*Aegilops tauschii*	33
232	1.77	2.02	5.31	Q40025	Beta-glucosidase	*H. vulgare*	2
233	1.74	7.62	32.06	A0A1D5XMK2	Uncharacterized protein	*T. timopheevii subsp. araraticumcum aestivum*	4
234	1.68	1.99	1.23	A0A1B6QIN6	Lipoxygenase	*Sorghum bicolor*	1

235	1.68	1.86	6.82	FABPH_HUMAN			1
236	1.64	1.82	1.54	M8BPX6	Uncharacterized protein	*Aegilops tauschii*	1
237	1.57	1.88	6.88	Q0D7S0	Allergen RA5B	*O. sativa subsp. japonica*	1
238	1.43	1.61	6.20	W5HY83	Uncharacterized protein	*T. timopheevii subsp. araraticumcum aestivum*	1
239	1.40	3.15	21.85	A0A0E0BKT2	Uncharacterized protein	*Leersia perrieri*	2
240	1.34	7.37	8.66	Q5I202	High molecular weight glutenin subunit 1Dy10.1t	*Aegilops tauschii*	10
241	1.34	1.53	2.90	F2CYL7	Predicted protein	*H. vulgare subsp. vulgare*	1
242	1.33	1.63	25.35	P81713	Bowman-Birk type trypsin inhibitor	*T. timopheevii subsp. araraticumcum aestivum*	3
243	1.32	1.61	8.90	F2DJC5	Predicted protein	*H. vulgare subsp. vulgare*	2
244	1.27	1.44	2.37	F2EJ82	Predicted protein	*H. vulgare subsp. vulgare*	1
245	1.27	1.44	9.09	F2E8C1	Predicted protein	*H. vulgare subsp. vulgare*	1
246	1.23	4.01	15.34	A8QRK0	Grain softness protein	*T. timopheevii subsp. araraticumcum dicoccoides*	2
247	1.23	1.39	13.92	W5G4V0	Uncharacterized protein	*T. timopheevii subsp. araraticumcum aestivum*	1
248	1.23	1.39	15.19	A0A1D6S463	Uncharacterized protein	*T. timopheevii subsp. araraticumcum aestivum*	1
249	1.20	1.36	5.88	T1MXP3	Uncharacterized protein	*T. timopheevii subsp. araraticumcum urartu*	1
250	1.19	13.21	34.40	Q68AN2	LMW-s KS2	*T. timopheevii subsp. araraticumcum aestivum*	26
251	1.18	1.34	2.98	P93639	Actin	*Zea mays*	1
252	1.18	1.34	5.26	W5FSA9	Uncharacterized protein	*T. timopheevii subsp. araraticumcum aestivum*	1
253	1.17	1.33	1.30	Q6ZFT9	Pyrophosphate--fructose 6-phosphate 1-phosphotransferase subunit alpha	*O. sativa subsp. japonica*	1
254	1.17	1.33	3.83	K7NED3	14-3-3 protein	*Setaria italica*	1
255	1.16	1.32	5.36	M0WY94	Uncharacterized protein	*H. vulgare subsp. vulgare*	1
256	1.15	1.31	5.68	F2EF58	Reticulon-like protein	*H. vulgare subsp. vulgare*	1
257	1.13	1.28	3.70	W5AVN7	Uncharacterized protein	*T. timopheevii subsp. araraticumcum aestivum*	1
258	1.12	1.28	3.49	A0A1D6CIB8	REVERSED Uncharacterized protein	*T. timopheevii subsp. araraticumcum aestivum*	1
259	1.10	1.26	10.32	M0UNX4	Uncharacterized protein	*H. vulgare subsp. vulgare*	1

260	1.06	1.28	4.59	Q9AXH7	1-Cys peroxiredoxin	*T. timopheevii subsp. araraticumcum turgidum subsp. durum*	1
261	1.05	1.20	16.28	W5ALQ5	Uncharacterized protein	*T. timopheevii subsp. araraticumcum aestivum*	1
262	1.03	1.19	2.64	R7VYY3	Alcohol dehydrogenase 1	*Aegilops tauschii*	1
263	1.01	29.58	41.43	A0A0E3Z589	Alpha-gliadin	*T. timopheevii subsp. araraticumcum aestivum*	19
264	1.00	1.15	10.67	K3ZER9	Uncharacterized protein	*Setaria italica*	1
265	0.97	1.29	19.74	H9NAV6	Superoxide dismutase	*T. timopheevii subsp. araraticumcum aestivum*	3

Supplementary Table 5. Protein identifications in the savoury spread (SS). Trypsin digestion peptides were identified after database searching against the Poaceae subset of the Uniprot database appended with the Common Repository of Adventitious Protein (cRAP) database using a 1% global false discovery rate (FDR) threshold.

N	Unused Score	Total Score	% Cov (95%)	Accession	Name	Species	Peptides (95%)
1	38.16	38.16	61.25	M0UEE6	Uncharacterized protein	*H. vulgare subsp. vulgare*	30
3	26.83	28.95	59.62	M0Z714	Uncharacterized protein	*H. vulgare subsp. vulgare*	20
4	24.68	24.68	64.33	P11643	Alpha-amylase/trypsin inhibitor	*H. vulgare*	32
5	21.68	21.68	63.09	P32936	Alpha-amylase/trypsin inhibitor	*H. vulgare*	18
6	17.56	17.56	38.71	I6TRT5	B3 hordein	*H. vulgare*	19
7	13.36	13.36	19.67	C3W8N0	Alpha-amylase	*H. vulgare subsp. vulgare*	9
8	13.20	13.21	53.29	Q546U1	Barley dimeric alpha-amylase inhibitor (Bdai-1)	*H. vulgare*	9
9	12.29	12.30	58.97	M0V3U0	Non-specific lipid-transfer protein	*H. vulgare subsp. vulgare*	13
10	12.21	12.22	21.20	M0XUU4	Uncharacterized protein	*H. vulgare subsp. vulgare*	7
11	12.13	12.15	61.64	F2EAF6	Predicted protein	*H. vulgare subsp. vulgare*	13
12	9.83	9.83	8.80	Q9SAU8	HSP70	*T. aestivum*	6
13	9.74	9.74	46.58	P16968	Alpha-amylase inhibitor BMAI-1	*H. vulgare*	6
14	9.41	9.41	13.80	F2D094	Predicted protein	*H. vulgare subsp. vulgare*	4
15	8.82	8.88	12.96	F2EFA8	ATP synthase subunit beta	*H. vulgare subsp. vulgare*	6
17	8.24	8.25	29.73	P01086	Trypsin inhibitor	*H. vulgare*	6
18	8.04	8.04	6.74	Q84LE9	D-Hordein	*H. vulgare*	5
19	8.00	8.00	35.62	M0VFV9	Uncharacterized protein (Similar to BAKER'S ASTHMA allergen BDP)	*H. vulgare subsp. vulgare*	5
20	7.82	7.96	37.01	W5DK32	Uncharacterized protein	*T. aestivum*	4
21	7.80	7.89	22.91	Q946Y9	Thaumatin-like protein	*H. vulgare*	4
22	6.25	8.70	38.10	Q6S5B1	Alpha amylase inhibitor CM3	*T. turgidum subsp. durum*	10
23	6.02	6.02	60.00	M0WPC3	Uncharacterized protein	*H. vulgare subsp. vulgare*	5
24	5.84	5.84	23.58	A0A1D6AZ07	Uncharacterized protein	*T. aestivum*	3
25	5.76	26.60	37.03	Q43492	Serpin-Z7	*H. vulgare*	19
26	5.69	5.73	38.62	M0YS73	Uncharacterized protein	*H. vulgare subsp. vulgare*	7
27	5.20	5.26	9.49	I6TEV2	Gamma 3 hordein	*H. vulgare*	3
28	5.06	5.11	10.31	Q40069	Peroxidase	*H. vulgare*	3
29	4.82	4.98	6.28	Q7DMU0	Storage protein	*T. aestivum*	3
30	4.73	4.81	11.09	F2D525	Elongation factor 1-alpha	*H. vulgare subsp. vulgare*	4

31	4.37	4.41	4.00	F2E7K9	Predicted protein	*H. vulgare subsp. vulgare*	2
32	4.10	8.14	18.45	I6R4A7	B-hordein	*H. vulgare subsp. vulgare*	8
33	4.02	4.82	5.64	F2DUX8	Predicted protein	*H. vulgare subsp. vulgare*	3
34	4.01	4.01	21.68	P34951	Trypsin inhibitor CMc	*H. vulgare*	3
35	4.00	8.00	10.97	F2DHX6	Predicted protein	*H. vulgare subsp. vulgare*	5
36	4.00	6.10	43.15	P93180	Pathogenesis-related protein 4	*H. vulgare*	7
37	4.00	4.00	15.00	Q75XW2	Glycolytic glyceraldehyde-3-phosphate dehydrogenase	*H. vulgare*	2
38	4.00	4.00	14.08	Q8S409	Hordoindoline A-1	*H. vulgare subsp. vulgare*	2
39	4.00	4.00	5.69	A0A1E5UZF1	ADP,ATP carrier protein, mitochondrial	*Dichanthelium oligosanthes*	2
40	4.00	4.00	16.28	D2KFH1	Avenin-like a4	*T. aestivum*	3
41	3.80	3.96	10.84	W5CWZ2	Uncharacterized protein	*T. aestivum*	2
42	3.61	3.74	2.42	F2E5M4	Predicted protein	*H. vulgare subsp. vulgare*	2
43	3.45	8.02	20.16	K4MR74	Alpha-amylase 2	*H. vulgare subsp. spontaneum*	6
44	3.08	13.88	43.08	M0Z9Q3	Uncharacterized protein	*H. vulgare subsp. vulgare*	11
45	2.78	2.93	5.09	K3XX16	Alpha-amylase	*Setaria italica*	2
46	2.68	3.79	5.07	O24346	ATP synthase subunit beta	*Sorghum bicolor*	2
47	2.64	2.87	4.56	F2DBC9	ATP synthase subunit alpha	*H. vulgare subsp. vulgare*	2
48	2.35	2.37	9.52	Q5IU17	Hordoindoline b2	*H. vulgare subsp. vulgare*	1
49	2.24	2.25	3.34	T1NPU7	Glyceraldehyde-3-phosphate dehydrogenase	*T. urartu*	1
50	2.13	2.13	1.68	M0XRX1	Uncharacterized protein	*H. vulgare subsp. vulgare*	1
51	2.03	2.03	1.27	F2DTZ6	Predicted protein	*H. vulgare subsp. vulgare*	1
52	2.01	2.01	5.03	M0Z3Q4	Uncharacterized protein	*H. vulgare subsp. vulgare*	1
53	2.01	2.01	4.27	F2E0T8	Predicted protein	*H. vulgare subsp. vulgare*	1
54	2.00	6.02	62.50	F2EKE4	Predicted protein	*H. vulgare subsp. vulgare*	4
55	2.00	6.00	4.59	C5WN47	Uncharacterized protein	*Sorghum bicolor*	4
56	2.00	5.72	5.13	F2DF25	Predicted protein	*H. vulgare subsp. vulgare*	4
57	2.00	4.00	15.85	Q3BER7	Putative vacuolar defense protein	*T. aestivum*	2
58	2.00	4.00	16.67	T1WIM8	Dimeric alpha-amylase inhibitor	*Dichanthelium oligosanthes*	2
59	2.00	2.00	8.94	W5I7T8	Uncharacterized protein	*T. aestivum*	1
60	2.00	2.00	4.42	W5FE16	Uncharacterized protein	*T. aestivum*	1
61	2.00	2.00	3.86	W5B9Q2	Uncharacterized protein	*T. aestivum*	1
62	2.00	2.00	6.28	W5ADG5	GTP-binding nuclear protein	*T. aestivum*	1

63	2.00	2.00	2.24	T1NDY3	Uncharacterized protein	*T. urartu*	1
64	2.00	2.00	2.56	T1LXH3	Uncharacterized protein	*T. urartu*	1
65	2.00	2.00	4.72	Q946Y8	Thaumatin-like protein TLP8	*H. vulgare*	1
66	2.00	2.00	8.03	Q84ZJ2	Uncharacterized protein	*Oryza sativa subsp. japonica*	1
67	2.00	2.00	3.98	Q7XTH1	OSJNBb0026L04.5 protein	*Oryza sativa subsp. japonica*	1
68	2.00	2.00	8.54	Q5URW0	Grain softness protein	*H. vulgare subsp. vulgare*	1
69	2.00	2.00	6.80	Q2V8X0	Limit dextrinase inhibitor	*H. vulgare*	1
70	2.00	2.00	3.51	Q10SE7	Beta-carotene hydroxylase, putative, expressed	*Oryza sativa subsp. japonica*	1
71	2.00	2.00	1.10	M8BM42	Putative SWI/SNF-related matrix-associated actin-dependent regulator of chromatin subfamily A member 3-like protein 3	*Aegilops tauschii*	1
72	2.00	2.00	3.55	M7YUS2	Anthocyanidin reductase	*T. urartu*	1
73	2.00	2.00	25.00	M0W1K5	Uncharacterized protein (Similar to Thionin BTH7)	*H. vulgare subsp. vulgare*	1
74	2.00	2.00	3.06	I1H2N2	Uncharacterized protein	*Brachypodium distachyon*	1
75	2.00	2.00	11.21	F5CPR2	Putative uncharacterized protein	*T. aestivum*	1
76	2.00	2.00	3.08	F2EHV0	Arp2/3 complex 34 kDa subunit	*H. vulgare subsp. vulgare*	1
77	2.00	2.00	1.89	F2E4B4	Predicted protein	*H. vulgare subsp. vulgare*	1
78	2.00	2.00	3.28	F2DFC7	Glutamate dehydrogenase	*H. vulgare subsp. vulgare*	1
79	2.00	2.00	4.89	C0PC41	Uncharacterized protein	*Zea mays*	1
80	2.00	2.00	4.59	B9G0Y1	Uncharacterized protein	*Oryza sativa subsp. japonica*	1
81	2.00	2.00	5.03	B6SPZ2	Glyceraldehyde-3-phosphate dehydrogenase	*Zea mays*	3
82	2.00	2.00	5.94	B6SNL4	60S ribosomal protein L13a-2	*Zea mays*	1
83	2.00	2.00	1.94	A0A1D6B6D1	Uncharacterized protein	*T. aestivum*	1
84	2.00	2.00	1.70	A0A0D9VMJ7	Uncharacterized protein	*Leersia perrieri*	1
85	2.00	2.00	10.00	A0A0A9LE21	Uncharacterized protein	*Arundo donax*	1
86	2.00	2.00	6.98	A0A077RWY0	Uncharacterized protein	*T. aestivum*	1
87	1.89	2.00	3.97	W5DQS5	Uncharacterized protein	*T. aestivum*	1
88	1.70	3.96	6.07	W5IC42	Uncharacterized protein	*T. aestivum*	2
89	1.68	4.15	5.67	F2DEU3	Predicted protein	*H. vulgare subsp. vulgare*	2

Supplementary Table 6. Ingredients and gluten peptide number identified in each food product type.

Code	Ingredients	LC-MS[a]	Total gluten peptides[b]
BC1	Whole white rice (59%), sugar, cocoa (3%), minerals (calcium carbonate, iron, zinc oxide), salt, flavours, dextrose, **barley malt extract**, vitamins (vitamin C, niacin, thiamin, riboflavin, folate). *Contains gluten containing cereals.*	W	**177** (wheat 154, oats 21, barley 1 & rye 1)
BC2	Corn (89%), sugar, salt, **barley malt** extract, vitamins (vitamin C, vitamin E [soy], niacin, riboflavin, thiamin, folate), minerals (iron, zinc oxide).	W, O	
BC3	Whole grain cereals (66%) (**whole wheat**, **rolled oats**), sultanas (17%), sugar, **triticale** (6%), apricot pieces (3.5%)(concentrated apricot puree, concentrated apple puree, invert sugar, humectant [glycerol], sugar, **wheat fibre**, gelling agent [pectin], acidity regulator [296], natural flavour, colour [paprika, lutein]), **barley malt extract**, natural flavour, salt, honey, mineral (iron), vitamins (niacin, riboflavin, folate, thiamin).	W, O	
BC4	**Whole wheat** (38%), sultanas (26%), **wheat bran** (25%), sugar, **barley malt extract**, salt, humectant (glycerol), minerals (iron, zinc oxide), vitamins (niacin, riboflavin, vitamin B6, thiamin, folate).	W, B, R	
BC5	**Whole grain cereals** (54%), (**whole wheat**, brown rice, **rolled oats**), corn (16%), fruit (13%) (sultanas, currants, dried apple), sugar, sliced almonds (2%), **wheat germ**, invert sugar syrup, **barley malt extract**, salt, vitamins (vitamin C, niacin, vitamin B6, riboflavin, folate), mineral (iron), preservative (220). *Contains gluten containing cereals.*	W, O	
BC6	Rice (42%), **whole grains** (24%) (**whole wheat**, **wholegrain oat flour**), **wheat gluten**, sugar, minerals (calcium carbonate, iron, zinc oxide), **oat fibre**, salt, **barley malt extract**, vitamins (niacin, vitamin B6, riboflavin, thiamin, folate).	W, B, O	
BC7	**Cereals** (49%) (**wheat flour**, **oatmeal**, maize flour), sugar, **wheat protein**, maltodextrin, molasses, **oat fibre**, salt, minerals (calcium carbonate, iron), **barley malt extract**, raising agent (potassium bicarbonate), flavour, natural colour (paprika, turmeric), vitamins (vitamin C, niacin, thiamin, riboflavin, vitamin B6, folate)	W, B, R, O	
BB1	Gluten free flour (rice flour, tapioca starch, maize starch, soy flour, emulsifier [soy lecithin], thickeners [415, 412, 464]), dried fruit (apricots [5%][preservative (220)], figs [4%], raisins [3%]), sunflower oil, quinoa flakes (7%), invert sugar syrup, modified starch (1422), sugar, vegetable fibre (chicory), honey, humectant (glycerol), natural flavour, raising agent (sodium bicarbonate), salt.	ND	
BB2	**Whole grains** (48%)(**whole wheat flour** [vitamins (thiamin, folate)], **rolled oats**), **wheat flour**, vegetable oil (sunola), invert syrup, orange piece (orange peel [4%], glucose syrup, sugar, acidity regulator [citric acid]), sugar, black chia seeds (3%), honey, humectant (glycerol), natural flavour, **oat fibre**, raising agents (ammonia bicarbonate, sodium bicarbonate), salt.	B, W, O	**156** (Wheat 135, Oats 15, Barley 5, Rye 1)
BB3	**Whole grain cereals** (40%) (**wheat flour**, **wheat** (12%), raw sugar, salt, **barley malt extract**, vitamins (niacin, thiamin, riboflavin, folate), mineral (iron)), sugar, sunflower and/or canola oil, wheat flour, macadamia nuts (5%), vegetable fibre, barley malt extract, salt, flavour, raising agents (500, 503). *Contains cereals containing gluten and tree nuts. May contain peanuts, other tree nuts, sesame, egg, milk and soy.*	B, W, R	
BM1	Filtered water, skim milk powder, cane sugar, soy protein, corn maltodextrin, vegetable oils (sunflower, canola), hi-maize starch, inulin, fructose, cocoa (0.5%), flavours, mineral (calcium), food acid (332), vegetable gums (460, 466, 407), stabiliser (452), salt, vitamins (C, niacin, A, B12, B6, B2, B1, folate). contains milk and soy. *Gluten free.*	ND	

BM2	Filtered water, skim milk powder, cane sugar, **wheat maltodextrin**, soy protein, vegetable oils (sunflower, canola), vegetable fibre, hi-maize starch, corn syrup solids, fructose, cocoa (0.5%), **oat flavours**, mineral (calcium), acidity regulator (332), flavours,, vegetable gums (460, 466, 407), stabiliser (452), salt, vitamins (C, niacin, A, B12, B6, B2, B1, folate). Contains milk, soy and cereals. *Containing gluten.*	O	**20** (Barley 11, Oats 9)
BM3	Reduced fat milk (75%), water, **extract of malt barley** or **malt barley** and rice, milk solids, sugar, cocoa, stabilisers (418, 452, 331), maltodextrin (corn), minerals (calcium, iron), vitamins (C, B3, B6, B2, D, B12). *Contains milk and barley. Contains gluten.*	B	
PD1	**Extract of malt barley** (16%) or **malt barley** and rice (total extract 35%), milk solids, sugar, cocoa, minerals (calcium, iron), maltodextrin (corn), vitamins (C, B3, A, B6, D, B2, B12), emulsifier (soy lecithin). *Contains gluten, milk and soy.*	B	**76** (Wheat 56, Barley 18, Oats 2)
PD2	**Barley malt extract** (41%), sugar, milk solids, maltodextrin, cocoa (9%) (with soy lecithin), mineral salts (341, 500), vegetable oil, vitamins & minerals (A, B1, B2, D, niacin, iron). *Contains gluten, milk and soy.*	B, W	
PD3	Sugar, cocoa (17), **barley malt extract**, **wheat starch**. Minerals: calcium phosphate (calcium, ferric pyrophosphate (iron)). Vitamins: calcium ascorbate (vitamin C), nicotinamide (niacin, vitamin B3), riboflavin (vitamin B2), thiamine mononitrate (vitamin B1), retinyl acetate (vitamin A), folic acid (folate), cholecalciffrol (vitamin D3). *This product contains gluten.*	B, W, O	
PD4	Sugar, fat reduced cocoa powder 18%, **cereals** 11% (**wheat**, **barley**, **malted wheat**), banana flakes 3%, sunflower lecithin, honey 0.3%, vanilla flavouring salt. *Contains: wheat. Contains gluten.*	B, W	
SS	Yeast extract (from yeast grown on **barley and wheat**), salt, potassium chloride, malt extract (from **barley**), colour (E150d) (contains preservative (sulphur dioxide)), vegetable extract (contains onion, celery) niacin, thiamine, riboflavin, folic acid. *Allergen statement: Contains barley and wheat.*	W, B	**13** (Barley 11, Wheat 2)

a. LC-MS detected W, wheat; B, barley, O, oats; R, rye.
b. Total number of gluten-derived peptides identified in combined search of each food product type.

Supplementary Table 7. Gluten peptides detected in breakfast cereals (BC). The spectral dataset was searched against the Poaceae subset of the Uniprot database. Only peptides with ≥95% confidence in this study abiding by trypsin digestion rules are reported. The mass error (ΔM) is presented in parts per million (ppm).

Protein name	Species	Accession	Peptide Sequence	ΔM (ppm)
High molecular weight glutenin subunit Dx5	*T. aestivum*	X2JUA0	ACQQVMDQQLR	-2.31
Low-molecular-weight glutenin subunit	*T. aestivum*	R4JFB5	AIIYSIVLQEQQQVR	2.25
Gamma-gliadin	*T. aestivum*	R9XT02	APFASIIAGIGGQ	11.49
Gamma-gliadin	*T. aestivum*	B6UKP6	APFASIVASIGGQ	1.00
Gamma-gliadin	*T. aestivum*	R9XU99	APFSSVVAGIGGQ	3.59
High molecular weight glutenin subunit	*T. aestivum*	A9YSK3	AQQPATQLPTVCR	1.08
High molecular weight glutenin subunit	*T. aestivum*	W6AX70	CCQQLR	2.47
Avenin-like protein	*T. aestivum*	V5M3L1	CHAIHIVVEAIIQQQSQQQWQEPQQQAQHK	-1.28
Type-b avenin-like protein	*T. aestivum*	A0A173DQZ4	CHAIHNVVEAIMQQQSQQQR	-1.32
Type-b avenin-like protein	*T. aestivum*	A0A173DQZ7	CHAIHSVVEAIMQQQSQQQWQER	-2.60
Gamma-gliadin	*T. aestivum*	I7KM78	CPAIHNIVHAIVMQQQHVDR	-4.75
Type-b avenin-like protein	*T. aestivum*	A0A173DQZ7	CQAIHNVAEAIR	-2.83
Alpha-gliadin protein	*T. aestivum*	X2KWE1	CQAIHNVAHAIIMHQQQQQQQEQQQQLQQ QQQQQLHQQR	-5.37
Alpha-gliadin	*T. aestivum*	K7X1L1	CQAIHNVVHAIILHHHQQQQQQPSSQVSYQQPQEQYPSGQGSFQSSQQNPQAQGSV QPQQLPQFQEIR	-6.00
Alpha-gliadin	*T. aestivum*	A0A0E3Z7F7	CQAIHNVVHAIILHQQQQQQQQQQQQK	-4.23
Alpha-gliadin	*T. aestivum*	K7XE90	CQAIHNVVHAIILHQQQR	-1.69
Alpha/beta-gliadin	*T. aestivum*	A0A0K2QJY6	CQAIQNVVHAIILHQQQQR	-3.64
Avenin-like a1	*T. aestivum*	Q2A784	CQAVCSVAQIIMR	1.27
Avenin-like a4	*T. aestivum*	D2KFH1	CQAVCSVAQVIMR	1.28
Avenin-like a3	*T. aestivum*	P0CZ08	CQAVCSVSQIIMR	0.24
High molecular weight glutenin subunit	*T. aestivum*	W6AX70	CRPVAVSQVVR	-1.63
Avenin-3	*T. urartu*	M8ANS4	DALLQQCSPVADMSFLR	-0.38
High molecular weight glutenin subunit Dx5	*T. aestivum*	X2JUA0	DISPECHPVVVSPVAGQYEQQIVVPPK	-1.56
Alpha/beta-gliadin	*T. aestivum*	I0IT51	DVIVLQQHNIAHESSQVLQQSSYQVLQQLCCQQLR	-1.75
Alpha-gliadin Gli-Ts4	*T. sphaerococcum*	A5JTR3	DVIVLQQHNIVR	5.54
High-molecular-weight glutenin subunit	*T. aestivum*	T2HRF3	DVSPECQPVGGGPVAR	2.10

X-type high molecular weight glutenin subunit 1Bx23	*T. turgidum*	W8Q5H7	DVSPGCRPITVSPGTR	-1.29
Alpha-gliadin	*T. aestivum*	R9XUM8	DVVLQQHNIAHAR	-0.90
Alpha-gliadin	*T. aestivum*	A0A1K0JNE4	DVVLQQHNIAHASSQVLQQSSYQLLQQLCCQR	-2.65
Alpha-gliadin	*T. aestivum*	K7X1L1	DVVLQQHNIAHASSQVLQQSSYQQLQQLCCQQLFQIPEQSR	-3.51
Alpha-gliadin	*T. aestivum*	R9XUP7	DVVLQQHNIAHASSQVLQQSTYQLLQQLCCQQLLQIPEQSR	-0.10
Alpha-gliadin	*T. spelta*	A0A0E3Z6U5	DVVLQQHNIAHGR	1.15
Alpha-gliadin protein	*T. aestivum*	X2KVI4	DVVLQQHSIAHGSSQVLQQSTYQLVQQLCCQQLWQIPEQSR	-3.22
Alpha/beta-gliadin	*T. aestivum*	I0IT55	DVVLQQPNIAHASSK	-1.44
Gliadin/avenin-like seed protein	*T. aestivum*	D2KFH0	ECCEQFR	2.61
Gamma-gliadin	*T. aestivum*	I7KM78	EFLLQQCNPEEK	0.88
High molecular weight glutenin subunit	*T. aestivum*	A0A060MZP1	EGEASEQLQCER	-0.85
Alpha-gliadin	*T. aestivum*	K7X0N8	ELCCQHLWQIPEQSQCQAIHNVVHAIILHQQQK	-5.93
High-molecular-weight glutenin subunit	*T. aestivum*	T2HRF3	ELQEHSLK	-0.50
Avenin-like a3	*T. aestivum*	P0CZ08	FGQPQQQQGQSFGQPQQQVPVEIMR	-1.45
Gamma-gliadin	*T. aestivum*	I7KM78	GFGQPQPQQLGQEMPMQPQHQLGQHSILPQQLAQYK	-1.55
High molecular weight glutenin subunit	*T. aestivum*	A9YSK3	GHYPASLQQPGQGQPGQR	-0.26
High molecular weight glutenin subunit	*T. aestivum*	A0A060MZP1	GQQGQQSGQGQQLGQGQQGQQPGQK	-2.09
High molecular weight glutenin subunit	*T. aestivum*	A9YSK3	GQQGYYPTSLQQPGQGQQGYYPTSLQHTGQR	-1.48
Y-type HMW glutenin subunit	*T. spelta*	Q7XZI2	GQQSGQGQQSGQGHQPGQGQQSGQEK	-2.78
Alpha-gliadin	*T. aestivum*	A0A0E3Z589	GSVQPQQLPQFEEIR	-0.90
LMW-glutenin P3-6	*T. aestivum*	Q8W3V4	GTFLQPHQIAR	-5.11
High molecular weight glutenin subunit	*T. aestivum*	W6AX70	HYPASLQQPGQGQQGHYTASLQQPGQGQQGHYPASLQQVGQGQQIGQLGQR	0.90
High molecular weight glutenin subunit Dx5	*T. aestivum*	X2JUA0	IFWGIPALLK	-5.07
High molecular weight glutenin subunit 1Ay protein	*T. dicoccoides*	J9Q8Q6	IGQGQQPEK	0.31
Gamma-gliadin	*T. aestivum*	I7KM78	IHDQERPQQSFLQQQPLIQQQPYPPQEPQQPLFPQK	-3.80
LMW glutenin pGM107	*T. aestivum*	Q6QGV8	ILPTMCSVNVPLYR	1.32
Alpha-gliadin	*T. turgidum subsp. paleocolchicum*	B1PDK7	ILQQQQLIPCR	3.24
High molecular weight glutenin subunit	*T. aestivum*	A0A060MZP1	LEGGDALSASQ	-1.40
High-molecular-weight glutenin subunit Bx7.1	*T. aestivum*	G4Y3Y0	LEGSDALSTR	-1.40

LMW-glutenin P3-6	*T. aestivum*	Q8W3V4	LEVMTSIALR	3.30
LMW-GS	*T. aestivum*	H6VLQ4	LFLQQQCSPVAMPQR	-2.64
Low-molecular-weight glutenin subunit group 2 type I	*T. aestivum*	Q8W3X5	LFLQQQCSPVAMPQSLAR	2.24
High molecular weight glutenin subunit	*T. aestivum*	W6AX70	LPWSTGLQMR	0.30
Alpha-gliadin storage protein	*T. spelta*	A0A1P8DT36	LQCQAIHNVVHAIILHQQQK	-2.16
High-molecular-weight glutenin By8	*T. aestivum*	Q0Q5D8	LVAVSQVVR	-0.82
High molecular weight glutenin subunit 1Ay protein	*T. dicoccoides*	J9Q8Q6	LVVDQQLAGR	2.34
High-molecular-weight glutenin subunit	*T. aestivum*	Q7XZB4	LVVDQQLASR	1.73
Alpha-gliadin	*T. aestivum*	A0A1K0JNE4	LWQIPEQSR	-1.27
Alpha-gliadin	*T. aestivum*	K7X0N8	MDVVLQQHNIVHGR	0.22
High molecular weight glutenin subunit	*T. aestivum*	W6AX70	MEGGDALSASQ	3.21
Type-b avenin-like protein	*T. aestivum*	A0A173DQZ7	MSLQALR	0.29
Avenin-like a1	*T. aestivum*	Q2A784	MVLQTLPLMCR	0.63
Gamma-gliadin	*T. aestivum*	U5U7C7	NDCQVMQQQCCQQLAQIPR	1.32
Gamma-gliadin	*T. aestivum*	B6UKM5	NFLLQQCNHVSLVSSLVSIILPR	-2.50
Alpha-gliadin	*T. aestivum*	K7XE90	NLALQTLPR	0.83
Glutenin	*T. aestivum*	B8ZX17	QEQQDQQPGQR	0.91
High-molecular-weight glutenin subunit	*T. aestivum*	T2HRF3	QGQQGQQSGQGQPR	0.49
High molecular weight glutenin subunit	*T. aestivum*	W6AX70	QGQQLEQGQQPGQGQQTR	-0.73
High molecular weight glutenin subunit	*T. aestivum*	A9YSK3	QGSYYPGQASPQQPGQGQQPGK	1.07
HMW glutenin subunit	*T. aestivum*	Q6RX93	QGYDSPYHVSAEQQAASPMVAK	-1.95
Low molecular weight glutenin subunit	*T. turgidum subsp. durum*	Q9FEQ2	QIAQLEVMTSIALR	2.61
Gamma-gliadin	*T. aestivum*	I7KM78	QLAHISEPSR	-0.86
Avenin-like protein	*T. aestivum*	V5M3L6	QLLEQMKPCVAFLQQK	0.44
Alpha/beta-gliadin MM1	*T. urartu*	M7ZZV2	QLPQFEEIR	2.63
Low-molecular-weight glutenin subunit	*T. aestivum*	A0A0S2GJT4	QLPQIPEQSR	-3.27
High molecular weight glutenin subunit	*T. aestivum*	A0A060MZP1	QLQQPEQGQQGQQPEQGQQGQQQR	-2.30
Type-b avenin-like protein	*T. aestivum*	A0A173DQZ7	QLSQIPEQFR	-1.67
High molecular weight glutenin subunit	*T. aestivum*	A0A060MZP1	QPGYYSTSPQQLGQGQPR	-0.86

Avenin-like a3	*T. aestivum*	P0CZ08	QQCCQPLAQISEQAR	-1.14
Gamma-gliadin	*T. aestivum x T. elongatum*	B6DQD5	QQCCQQLAR	0.31
High-molecular-weight glutenin subunit	*T. aestivum*	T2HRF3	QQDQQSGQGQQPGQR	-0.15
HMW-glutenin By subunit	*T. turgidum*	Q6UJY7	QQLGQGQQTR	4.38
High molecular weight glutenin subunit	*T. aestivum*	A9YSK3	QQPGQGQHPEQGK	-0.69
High molecular weight glutenin subunit	*T. aestivum*	W6AX70	QQPGQGQQIGQGQQLGQGR	0.00
High molecular weight glutenin subunit	*T. aestivum*	A0A060MZP1	QQPGQGQQLR	-4.82
High molecular weight glutenin subunit	*T. aestivum*	W6AX70	QQPGQGQQPEQGQQPGQGQQGYYPTSPQQPGQGK	-0.94
High molecular weight glutenin subunit	*T. aestivum*	W6AX70	QQPGQGQQTR	2.38
Avenin-like a1	*T. aestivum*	Q2A784	QQQGQSFGQPQQQVPVEIMR	1.58
Avenin-like a4	*T. aestivum*	D2KFH1	QQQGQSFGQPQQQVQSFSQPQHQVPIEITR	-1.27
Type-b avenin-like protein	*T. aestivum*	A0A173DQZ7	QQQPQQQWQGMYQPQQPAQHESIR	-2.32
High molecular weight glutenin subunit 1Dy protein	*T. aestivum*	V9TRL3	QVVDQQLAGR	0.77
High-molecular-weight glutenin subunit	*T. aestivum*	T2HRF3	QVVDQQLR	-0.68
High molecular weight glutenin subunit 1Bx protein	*T. dicoccoides*	A0A142ESP3	QYEQQPVVPSK	0.84
High molecular weight glutenin subunit	*T. aestivum*	W6AX70	QYEQTVVPPK	-1.34
Gamma-gliadin	*T. aestivum*	B6UKP1	RPLFQLVQGQGIIQPQQPAQLEVIR	-7.35
Gamma-gliadin	*T. aestivum*	B6UKP1	SDCQVMQQQCCQQLAQIPQQLQCAAIHSVVHSIIMQQEQR	-1.02
Gamma-gliadin	*T. turgidum*	B6UKL5	SDCQVMQQQCCQQLAQIPR	1.33
Gamma-gliadin	*T. urartu*	B6UKS0	SDCQVMR	1.02
Gliadin/avenin-like seed protein	*T. aestivum*	D2KFH0	SDQPQQSFPQQPQQK	1.31
Gamma-gliadin	*T. aestivum*	R9XWD0	SFIQPSLQQQLNPCK	2.60
Gamma-gliadin	*T. urartu*	B6UKS0	SLVLGTLPTMCNVFVPPECSTTK	-3.00
Gamma-gliadin	*T. aestivum*	R9XU99	SLVLQTLPTMCNVYVPPECSIIK	-0.81
Gamma gliadin	*T. aestivum*	Q6EEX1	SLVLQTLPTMCNVYVPPYCSTFR	-1.83
Low-molecular-weight glutenin subunit	*T. urartu*	A0A089XB95	SQMLEQSICHVMQQQCCQQLR	0.00
LMW-GS	*T. aestivum*	H6VLQ4	SQMLQQCSCHVMQQQCCQQLPQIPQQSR	-6.18
Low-molecular-weight glutenin subunit Glu-A3	*T. aestivum*	X2JAE7	SQMLQQSICHVMQQQCCQQLR	0.45
Low molecular weight glutenin subunit	*T. turgidum subsp. durum*	Q9FEQ2	SQMLQQSICHVMQR	-0.91

LMW-s KS2	*T. aestivum*	Q68AN2	SQMLQQSSCHMMQQQCCQQLPQIPQQSR	-4.06
LMW-GS	*T. aestivum*	R9XVA5	SQMLQQSSCHVMQQQCCQQLLQIPQQSR	-0.78
Low-molecular-weight glutenin subunit	*T. aestivum*	R4JBK0	SQMLWQSSCHVMQQQCCQQLPR	-2.60
Low-molecular-weight glutenin subunit	*T. aestivum*	A0A0S2GJT4	SQMLWQSSCHVMQQQCCR	1.25
Low molecular weight glutenin	*T. aestivum subsp. tibeticum*	Q7Y074	SQMWQQSSCHVMQQQCCQQLPQIPEQSR	-1.60
Low-molecular-weight glutenin subunit	*T. aestivum*	H9XGZ9	SQMWQQSSCHVMQQQCCQQLPR	-3.77
Low molecular weight glutenin	*T. aestivum*	Q5MFP0	SQMWQQSSCHVMQQQCCQQLQQIPEQSR	0.28
LMW glutenin	*T. turgidum subsp. durum*	Q41603	SQMWQQSSCHVMQQQCCQQLSQIPEQSR	-1.88
Low-molecular-weight glutenin subunit protein 1-50	*T. aestivum x T. elongatum*	A9UID2	SQVLQQSICHVMQQQCCQQLR	13.51
Alpha/beta-gliadin	*T. aestivum*	A0A0K2QJA2	SQVLQQSTYQLLQELCCQHLWQIPEQSQCQAIHNVVHAIILR	-0.86
Alpha-gliadin	*T. aestivum*	K7X0N8	SQVLQQSTYQLLR	1.95
Avenin-3	*T. urartu*	M8ANS4	SQVVQHSSCLVMWEQCCQQLK	-2.60
High molecular weight glutenin subunit 1Dy protein	*T. aestivum*	V9TRL3	SVAVSQVAR	-0.07
Low molecular weight glutenin subunit	*T. aestivum*	Q75ZV8	TLPMMCR	0.46
LMW-GS	*T. aestivum*	R9XWE6	TLPTMCNVNVPLYR	2.77
Low-molecular-weight glutenin subunit	*T. aestivum*	Q1ZZT4	TLPTMCR	-1.74
Low molecular weight glutenin subunit	*T. aestivum*	K7WV92	TLPTMCSVNVPLYR	0.96
Low-molecular-weight glutenin subunit	*T. aestivum*	R4JAP5	TLPTMCSVNVPVYGTTTGVPFGVGTR	0.35
Low-molecular-weight glutenin subunit	*T. aestivum*	A0A0S2GJR0	TLPTMCSVNVPVYGTTTIVPFGVGTR	-0.52
High molecular weight glutenin subunit 1Ay protein	*T. dicoccoides*	J9Q8Q6	TSLQQPGQR	4.03
Gamma-gliadin	*T. aestivum*	I7KM78	TSQQNSCQLK	10.34
LMW-GS	*T. aestivum*	R9XVA5	TTTSVPFGVGTGVGSY	0.32
Gliadin/avenin-like seed protein	*T. aestivum*	D2KFH0	TVQSFFEQQLISCR	1.26
Low-molecular-weight glutenin subunit Glu-A3	*T. aestivum*	X2JAE7	VFLQQQCIPVAMQR	1.42
Low molecular weight glutenin subunit	*T. macha*	B9VUV5	VFLQQQCNPVAMPQR	-0.27
Low molecular weight glutenin subunit B3-3	*T. aestivum*	D3UAL8	VFLQQQCNPVAMPQSLAR	5.62
Low-molecular-weight glutenin subunit	*T. aestivum*	Q1ZZT4	VFLQQQCSHVAMSQR	-2.35
Low molecular weight glutenin subunit	*T. turgidum subsp. durum*	Q9XGE9	VFLQQQCSPMAMPQSLAR	-3.39
LMW glutenin	*T. turgidum subsp. durum*	Q41603	VFLQQQCSPVAIPQR	5.10

Low-molecular-weight glutenin subunit	*T. aestivum*	A0A0S2GJR0	VFLQQQCSPVAIPQSLAR	3.11
LMW-glutenin P3-6	*T. aestivum*	Q8W3V4	VFLQQQCSPVAMPQHLAR	-1.39
LMW-GS	*T. aestivum*	R9XWE6	VFLQQQCSPVAMPQR	2.12
Low-molecular-weight glutenin subunit	*T. aestivum*	R4JFB5	VFLQQQCSPVAMPQSLAR	1.42
Low-molecular-weight glutenin subunit	*T. aestivum*	R4JAP5	VFLQQQCSPVATPQILAR	0.12
Low-molecular-weight glutenin subunit	*T. aestivum*	R4JFB5	VNVPLYR	0.85
Low-molecular-weight glutenin subunit	*T. aestivum*	R4JDK6	VPFGVGTGVGGY	4.51
Alpha/beta-gliadin	*T. aestivum*	A0A0K2QJA2	VPVPQLQPK	2.37
Alpha-gliadin	*T. aestivum*	A0A0E3Z516	VPVPQLQPQNPSQQQPQK	1.80
High molecular weight glutenin subunit	*T. aestivum*	W6AX70	VQQPATQLPIMCR	0.71
High molecular weight glutenin subunit	*T. aestivum*	A0A060MZP1	YYPTSPQQPGQEQQPR	1.72
High molecular weight glutenin subunit	*T. aestivum*	A0A060MZP1	YYPTSSQQPGQLQQLAQGQQGQQPER	0.17
Gliadin-like avenin	*A. sativa*	L0L6J0	AFALQALPAMCDVYVPPHCPVATVPLSGF	1.30
Avenin	*A. sativa*	P27919	CDAIWR	0.60
Avenin	*A. sativa*	P27919	ALPVDVLANAYR	2.53
Avenin	*A. magna*	I4EP67	CPAIHSVVQAIILQK	-4.30
Gliadin-like avenin	*A. sativa*	L0L5H3	CSPVEMVPFLR	2.38
Avenin	*A. sativa*	P27919	DFPITWPWK	-2.88
Avenin	*A. sativa*	P27919	ELGGFFGTQQGLIGK	6.61
Avenoindoline	*A. sativa*	J7FKU9	GGCQELLGECCSR	1.52
Avenin	*A. sativa*	P27919	GQESGVFTPK	0.00
Avenin protein	*A. murphyi*	G8ZCW5	LEQIPEQLR	1.41
Avenoindoline	*A. sativa*	J7FKU9	LGQMPPQCR	1.24
Avenin	*A. sativa*	P27919	NECCQLLGQMPSECR	-0.06
Avenin-E	*A. sativa*	Q09114	QAICQVAR	-0.06
Gliadin-like avenin	*A. sativa*	L0L6J0	QAICQVTR	-1.32
Gliadin-like avenin	*A. sativa*	L0L6J0	QFLVQQCSPVAAVPFLR	-1.56
Gliadin-like avenin	*A. sativa*	L0L6J0	QLAQIPEQLR	-3.68
Avenin	*A. magna*	I4EP58	QLAQIPEQVR	1.76
Gliadin-like avenin	*A. sativa*	L0L5H3	QLAQIPR	-0.07

Avenin	*A. sativa*	Q2EPY2	QLEQIPEQLR	-1.27
Avenin	*A. magna*	I4EP67	QQCCQQLAQIPEQVR	-0.26
Gliadin-like avenin	*A. sativa*	L0L5H3	QSTCHVMR	1.14
Avenin	*A. magna*	I4EP67	SQILQQSSCQVMK	0.40
Avenin	*A. sativa*	Q2EPY2	SQILQQSSCQVMR	1.87
B-hordein	*H. vulgare subsp. vulgare*	Q3YAF9	VFLQQQCSPVR	7.36
Alpha-gliadin storage protein	*S. strictum subsp. africanum*	F4ZL28	SQILQENVCAVMR	1.42

Supplementary Table 8. Gluten peptides detected in breakfast bars (BB). The spectral dataset was searched against the Poaceae subset of the Uniprot database. Only peptides with ≥95% confidence in this study abiding by trypsin digestion rules are reported. The mass error (ΔM) is presented in parts per million (ppm).

Protein name	Species	Accession	Sequence	ΔM (ppm)
High molecular weight glutenin subunit	*T. aestivum*	A0A0K0KDM6	ACQQVMDQQLR	-0.18
HMW glutenin subunit 1Bx13	*T. aestivum*	A5HMG1	AGSFYPSK	2.78
High molecular weight glutenin subunit	*T. aestivum*	Q75ZV8	AIIYSIVLQEQQQVR	-2.39
Gamma-gliadin	*T. aestivum*	Q94G97	APFASIVADIGGQ	1.86
High molecular weight glutenin subunit	*T. aestivum*	A0A060MZP1	AQQLAAQLPAMCR	0.67
High molecular weight glutenin subunit	*T. aestivum*	A0A0K0KDM6	AQQPATQLPTVCR	0.91
High molecular weight glutenin subunit	*T. aestivum*	W6AX70	CCQQLR	1.77
Avenin-like b2	*T. aestivum*	P0CZ05	CHAIHIVVEAIIQQQSQQQWQEPQQQAQHK	-3.31
Type-b avenin-like protein	*T. aestivum*	A0A173DQZ4	CHAIHNVVEAIMQQQSQQQR	-3.86
Type-b avenin-like protein	*T. aestivum*	A0A173DQZ7	CHAIHSVVEAIMQQQSQQQWQER	-1.73
Gamma-gliadin	*T. aestivum*	I7KM78	CPAIHNIVHAIVMQQQHVDR	-5.57
Avenin-3	*T. urartu*	M8AQI5	CQAIHNVAESIR	-0.70
Alpha-gliadin	*T. turgidum subsp. durum*	D2X6D9	CQAIHNVAHAIIMHQQQQQQQEQQQQLQQQQQQQLHQQ	-6.07
Alpha-gliadin storage protein	*T. spelta*	A0A1P8DTA3	CQAIHNVVHAIILHQQQQQQQQQQQK	-1.85
Alpha-gliadin	*T. aestivum*	A0A0E3Z7F7	CQAIHNVVHAIILHQQQQQQQQQQQQK	-5.35
Alpha-gliadin	*T. aestivum*	K7XE90	CQAIHNVVHAIILHQQQR	-1.47
Alpha/beta-gliadin	*T. aestivum*	A0A0K2QJY6	CQAIQNVVHAIILHQQQQR	-2.35
Avenin-like a1	*T. aestivum*	Q2A784	CQAVCSVAQIIMR	2.55
Avenin-like a4	*T. aestivum*	D2KFH1	CQAVCSVAQVIMR	0.64
Avenin-like a3	*T. aestivum*	P0CZ08	CQAVCSVSQIIMR	-0.39
High molecular weight glutenin subunit	*T. aestivum*	W6AX70	CRPVAVSQVVR	0.77
High molecular weight glutenin subunit	*T. aestivum*	A0A0K0KDM6	DISPECHPVVVSPVAGQYEQQIVVPPK	-1.56
High molecular weight glutenin subunit	*T. aestivum*	A0A060MZP1	DVSPECQPVGGGPVAR	0.00
HMW glutenin subunit Gx	*T. timopheevii subsp. araraticum*	D7RT26	DVSPGCRPITVGPGMR	-15.96
Alpha-gliadin storage protein	*T. spelta*	A0A1P8DTD6	DVVLQQHNIAHAR	-2.03
Alpha-gliadin storage protein	*T. spelta*	A0A1P8DSZ1	DVVLQQHNIAHASSQVLQQSSYQLLQQLCCQR	-2.33

Alpha-gliadin	*T. aestivum*	A0A0E3Z589	DVVLQQHNIAHASSQVLQQSSYQQLQQLCCQQLFQIPEQSR	-1.91
Alpha-gliadin	*T. turgidum subsp. durum*	D2X6D9	DVVLQQHNIAHASSQVLQQSTYQLLQQLCCQQLLQIPEQSR	-0.91
Alpha-gliadin storage protein	*T. spelta*	A0A1P8DT83	DVVLQQHNIAHGR	-2.38
Alpha-gliadin	*T. aestivum*	K7XE90	DVVLQQHSIAHGSSQVLQQSTYQLVQQLCCQQLWQIPEQSR	-0.71
Alpha/beta-gliadin	*T. aestivum*	I0IT55	DVVLQQPNIAHASSK	-1.90
Gamma-gliadin	*T. aestivum*	I7KM78	EFLLQQCNPEEK	1.27
High molecular weight glutenin subunit	*T. aestivum*	A0A060MZP1	EGEASEQLQCER	0.17
High molecular weight glutenin subunit	*T. dicoccoides*	A0A142ESP3	ELEACQQVVDQQLR	-0.07
High molecular weight glutenin subunit	*T. aestivum*	W6AX70	ELQESSLEACR	-0.92
Avenin-like a3	*T. aestivum*	P0CZ08	FGQPQQQQGQSFGQPQQQVPVEIMR	-1.28
Gamma-gliadin	*T. aestivum*	I7KM78	GFGQPQPQQLGQEMPMQPQHQLGQHSILPQQLAQYK	-1.55
High molecular weight glutenin subunit	*T. aestivum*	A0A0K0KDM6	GGSFYPGETTPPQQLQQR	1.72
High molecular weight glutenin subunit	*T. aestivum*	A0A060MZP1	GQQGQQSGQGQQLGQGQQGQQPGQK	-2.00
High molecular weight glutenin subunit	*T. aestivum*	V9TRL3	GQQGYYPTSLQQPGQGQQGYYPTSLQHTGQR	-1.48
Alpha-gliadin	*T. aestivum*	A0A0E3Z589	GSVQPQQLPQFEEIR	0.14
Low molecular weight glutenin	*T. aestivum subsp. tibeticum*	Q7Y074	GTFLQPHQIAR	-0.48
Gamma-gliadin	*T. aestivum*	I7KM78	IHDQERPQQSFLQQQPLIQQQPYPPQEPQQPLFPQK	-2.91
High molecular weight glutenin subunit	*T. aestivum*	Q84U14	ILPTMCSVNVPLYR	1.47
High molecular weight glutenin subunit	*T. dicoccoides*	A0A142ESP3	LEGSDALSAR	-0.78
Low molecular weight glutenin	*T. aestivum subsp. tibeticum*	Q7Y074	LEVMTSIALR	3.19
LMW-GS	*T. aestivum*	H6VLQ4	LFLQQQCSPVAMPQR	0.34
High molecular weight glutenin subunit	*T. aestivum*	W6AX70	LPWSTGLQMR	2.33
Alpha-gliadin storage protein	*T. spelta*	A0A1P8DT72	LQCQAIHNVVHAIILHQQQK	-3.59
High-molecular-weight glutenin By8	*T. aestivum*	Q0Q5D8	LVAVSQVVR	-0.19
High molecular weight glutenin subunit	*T. dicoccoides*	A0A142ESP5	LVVDQQLAGR	3.23
Alpha-gliadin storage protein	*T. spelta*	A0A1P8DSZ1	LWQIPEQSR	0.11
Alpha-gliadin	*T. aestivum*	K7X0N8	MDVVLQQHNIVHGR	2.37
LMW-m glutenin subunit 17	*T. aestivum*	V9P7D3	METSCIPGLERPWQQQPLQQK	-0.86
Type-b avenin-like protein	*T. aestivum*	A0A173DQZ7	MSLQALR	2.71

Avenin-like a1	*T. aestivum*	Q2A784	MVLQTLPLMCR	-3.50
Gamma-gliadin	*T. aestivum*	Q94G93	NFLLQQCNHVSLVSSLVSIILPR	0.09
Gamma-gliadin	*T. aestivum x T. elongatum*	B6DQD5	NILLQQCKPASLVSSLWSIIWPQSDCQVMR	1.72
Alpha-gliadin storage protein	*T. spelta*	A0A1P8DTD6	NLALQTLPR	2.03
High molecular weight glutenin subunit	*T. aestivum*	A0A060MZP1	QGQQGQQSGQGQPR	0.33
High molecular weight glutenin subunit	*T. aestivum*	W6AX70	QGQQLEQGQQPGQGQQTR	-0.92
High molecular weight glutenin subunit	*T. aestivum*	V9TRL3	QGSYYPGQASPQQPGQGQQPGK	1.07
High molecular weight glutenin subunit	*T. aestivum*	A0A0K0KDM6	QGYDSPYHVSAEQQAASPMVAK	0.10
Gamma-gliadin	*T. aestivum*	I7KM78	QLAHISEPSR	-1.61
Avenin-like b2	*T. aestivum*	P0CZ05	QLLEQMKPCVAFLQQK	0.25
Low-molecular-weight glutenin subunit	*T. aestivum*	R4JFQ3	QLPQIPEQSR	-2.66
Type-b avenin-like protein	*T. aestivum*	A0A173DQZ7	QLSQIPEQFR	-2.16
Avenin-3	*T. urartu*	M8AQI5	QLVQIPEQTR	6.05
Gamma-gliadin	*T. aestivum*	H9BFB6	QPAQLEAIR	3.22
High molecular weight glutenin subunit	*T. aestivum*	A0A060MZP1	QPGYYSTSPQQLGQGQPR	0.00
Avenin-like a4	*T. aestivum*	D2KFH1	QQCCQPLAQISEQAR	-1.95
Gamma-gliadin	*T. aestivum x T. elongatum*	B6DQD5	QQCCQQLAR	-4.10
High molecular weight glutenin subunit	*T. aestivum*	A0A060MZP1	QQDQQSGQGQQPGQR	-0.88
HMW-glutenin By subunit	*T. turgidum*	Q6UJY7	QQLGQGQQTR	-1.82
High molecular weight glutenin subunit	*T. aestivum*	V9TRL3	QQPGQGQHPEQGK	-1.72
High molecular weight glutenin subunit	*T. aestivum*	W6AX70	QQPGQGQQIGQGQQLGQGR	-2.70
High molecular weight glutenin subunit	*T. aestivum*	A0A060MZP1	QQPGQGQQLR	-3.65
High molecular weight glutenin subunit	*T. aestivum*	W6AX70	QQPGQGQQPEQGQQPGQGQQGYYPTSPQQPGQGK	-1.75
Avenin-like a1	*T. aestivum*	Q2A784	QQQGQSFGQPQQQVPVEIMR	2.10
Avenin-like a4	*T. aestivum*	D2KFH1	QQQGQSFGQPQQQVQSFSQPQHQVPIEITR	2.19
Type-b avenin-like protein	*T. aestivum*	A0A173DQZ7	QQQPQQQWQGMYQPQQPAQHESIR	-1.24
Avenin-like b5	*T. aestivum*	A7XUQ5	QQQPQQQWQGMYQPQQPAQLESIR	2.00
High molecular weight glutenin subunit	*T. aestivum*	W6AX70	QVVDQQLAGR	-3.40
HMW glutenin subunit Ax2	*T. aestivum*	Q41553	QVVDQQLR	1.18

High-molecular-weight glutenin subunit	*T. aestivum*	A0A060MZP1	QYEQQVVVPPK	1.30
High-molecular-weight glutenin subunit	*T. aestivum*	W6AX70	QYEQTVVPPK	1.85
Avenin-like b5	*T. aestivum*	A7XUQ5	QYQQQQQPGQR	-0.44
Gamma-gliadin	*T. aestivum*	B6UKP1	RPLFQLVQGQGIIQPQQPAQLEVIR	-5.90
Gamma-gliadin	*T. aestivum*	B6UKP1	SDCQVMQQQCCQQLAQIPQQLQCAAIHSVVHSIIMQQEQR	0.71
Gamma-gliadin	*T. aestivum*	U5U9Q4	SDCQVMQQQCCQQLAQIPR	-1.64
Gamma-gliadin	*T. aestivum*	R9XU99	SFIQPSLQQQLNPCK	1.64
Gamma-gliadin	*T. aestivum x T. elongatum*	B6DQD5	SLVLQTLPSMCNVYVPPECSIMR	-1.60
LMW-GS	*T. aestivum*	H6VLQ4	SQMLQQCSCHVMQQQCCQQLPQIPQQSR	-7.43
Low-molecular-weight glutenin subunit	*T. aestivum*	A0A0S2GJQ4	SQMLQQSICHVMQQQCCQQLR	-2.90
Low-molecular-weight glutenin subunit	*T. aestivum*	M1GL80	SQMLQQSICHVTQQQCCQQLR	4.41
LMW-GS P-31	*T. aestivum*	Q00M56	SQMLQQSSCHVMQQQCCQQLLQIPQQSR	-1.62
Low-molecular-weight glutenin subunit	*T. aestivum*	R4JAP5	SQMLWQSSCHVMQQQCCQQLPQIPEQSR	-1.32
Low-molecular-weight glutenin subunit	*T. aestivum*	R4JBK0	SQMLWQSSCHVMQQQCCQQLPR	-3.03
Low-molecular-weight glutenin subunit	*T. aestivum*	R4JFQ3	SQMLWQSSCHVMQQQCCR	0.42
Low molecular weight glutenin	*T. aestivum subsp. tibeticum*	Q7Y074	SQMWQQSSCHVMQQQCCQQLPQIPEQSR	-1.60
Low molecular weight glutenin subunit	*T. aestivum*	K7XRG7	SQMWQQSSCHVMQQQCCQQLQQIPEQSR	-1.38
LMW-glutenin	*T. aestivum*	B5ANT3	SQMWQQSSCHVMQQQCCQQLSQIPEQSR	-1.04
Alpha-gliadin storage protein	*T. spelta*	A0A1P8DT72	SQVLQQSTYQLLQELCCQHLWQIPEK	-0.15
Alpha/beta-gliadin	*T. aestivum*	A0A0K2QJA2	SQVLQQSTYQLLQELCCQHLWQIPEQSQCQAIHNVVHAIILR	-0.86
Alpha-gliadin	*T. aestivum*	K7X0N8	SQVLQQSTYQLLR	2.97
High molecular weight glutenin subunit	*T. aestivum*	V9TRL3	SVAVSQVAR	0.13
Low molecular weight glutenin subunit	*T. aestivum*	Q75ZV8	TLPMMCR	1.21
Low-molecular-weight glutenin GLU-B3	*T. turgidum*	A7XDG0	TLPTMCNVNVPLYR	0.15
Low molecular weight glutenin subunit	*T. aestivum*	B2Y2S3	TLPTMCR	0.14
Low-molecular-weight glutenin subunit	*T. aestivum*	R4JAP5	TLPTMCSVNVPVYGTTTGVPFGVGTR	-0.53
Gamma-gliadin	*T. aestivum*	I7KM78	TSQQNSCQLK	2.15
Low-molecular-weight glutenin subunit	*T. aestivum*	B2Y2S3	TTTNVPFGVGTGVGSY	2.20
Gliadin/avenin-like seed protein	*T. aestivum*	D2KFH0	TVQSFFEQQLISCR	0.91

Low-molecular-weight glutenin subunit	*T. aestivum*	A0A0S2GJQ4	VFLQQQCIPVAMQR	-0.07
Low molecular weight glutenin	*T. aestivum subsp. tibeticum*	Q7Y074	VFLQQQCNPVAMPQR	2.02
LMW-m glutenin subunit 45	*T. aestivum*	V9P760	VFLQQQCSHVAMSQR	-1.54
Low-molecular-weight glutenin subunit	*T. aestivum*	A0A0S2GJR0	VFLQQQCSPVAIPQSLAR	3.11
LMW-m glutenin subunit 0154A5-M	*T. aestivum*	B2BZC7	VFLQQQCSPVAMPQHLAR	-1.39
Low-molecular-weight glutenin subunit	*T. aestivum*	Q84U14	VFLQQQCSPVAMPQR	-0.68
Low-molecular-weight glutenin subunit	*T. aestivum*	Q75ZV8	VFLQQQCSPVAMPQSLAR	-2.49
Low-molecular-weight glutenin subunit	*T. aestivum*	R4JAP5	VFLQQQCSPVATPQILAR	2.02
Low-molecular-weight glutenin subunit	*T. aestivum*	Q75ZV8	VNVPLYR	-0.36
Alpha/beta-gliadin	*T. aestivum*	A0A0K2QJA2	VPVPQLQPK	4.19
Alpha-gliadin	*T. aestivum*	K7X1I9	VPVPQLQPQNPSQQQPQK	1.80
High molecular weight glutenin subunit	*T. aestivum*	W6AX70	VQQPATQLPIMCR	2.06
High molecular weight glutenin subunit	*T. aestivum*	A0A060MZP1	YYPTSPQQPGQEQQPR	-2.76
HMW glutenin subunit Ax2	*T. aestivum*	Q41553	YYPTSSQQPGQLQQLAQGQQGQQPER	-0.41
Avenin	*A. sativa*	P27919	ALPVDVLANAYR	-0.56
Avenin	*A. magna*	I4EP67	CPAIHSVVQAIILQK	-3.06
Type-b avenin-like protein	*A. magna*	A0A173DQZ7	CQAIHNVAEAIR	-3.98
Avenin	*A. sativa*	P27919	DFPITWPWK	-1.44
Avenin	*A. sativa*	P27919	ELGGFFGTQQGLIGK	7.24
Gliadin-like avenin	*A. sativa*	L0L6J0	QAICQVTR	4.63
Gliadin-like avenin	*A. sativa*	L0L6J0	QFLVQQCSPVAAVPFLR	-0.25
Gliadin-like avenin	*A. sativa*	L0L6J0	QLAQIPEQLR	-2.45
Avenin	*A. ventricosa*	I4EP59	QLAQIPEQVR	-1.65
Avenin	*A. sativa*	Q2EPY2	QLEQIPEQLR	-0.97
Avenin protein	*A. murphyi*	G8ZCW5	LEQIPEQLR	-0.98
Avenin	*A. magna*	I4EP67	QQCCQQLAQIPEQVR	-2.01
Avenin	*A. sativa*	Q09071	QSTCHVMR	-0.42
Avenin	*A. sativa*	P27919	NECCQLLGQMPSECR	-0.97
B hordein	*H. vulgare subsp. vulgare*	Q2XQF1	IVPLAIDTR	2.94

Gamma 3 hordein	*H. vulgare*	I6TEV2	QQCCQQLANINEQSR	1.56
B hordein	*H. vulgare subsp. vulgare*	Q2XQF1	TLPTMCSVNVPLYR	1.63
B3 hordein	*H. vulgare*	I6TRT5	VFLQQQCSPVAMSQR	2.20
B3 hordein	*H. vulgare subsp. vulgare*	Q3YAF9	VFLQQQCSPVR	5.38
Alpha-gliadin storage protein	*S. strictum subsp. africanum*	F4ZL28	SQILQENVCAVMR	4.97
High molecular weight glutenin subunit	*S. cereale x T. aestivum*	S5SCQ8	DVSPGCRPITVSPGTR	-1.73
High molecular weight glutenin subunit	*S. cereale x T. aestivum*	S5SCQ8	LEGSDALSTR	-2.33
High-molecular-weight glutenin subunit	*S. cereale x T. aestivum*	S5SCQ8	QYEQQPVVPSK	0.84

Supplementary Table 9. Gluten peptides detected in milk-based breakfast drinks (BM). The spectral dataset was searched against the Poaceae subset of the Uniprot database. Only peptides with ≥95% confidence in this study abiding by trypsin digestion rules are reported. The mass error (ΔM) is presented in parts per million (ppm).

Protein name	Species	Accession	Peptide sequence	ΔM (ppm)
B3-hordein	*H. vulgare*	I6TRT5	AIVYSIVLR	6.27
D-Hordein	*H. vulgare*	Q84LE9	AQQLAAQLPAMCR	5.61
D-Hordein	*H. vulgare*	Q84LE9	DVSPECRPVALSQVVR	1.48
D-Hordein	*H. vulgare*	Q84LE9	ELQESSLEACR	2.87
B-hordein	*H. vulgare*	Q3YAF9	ILPFGIDTR	12.91
Gamma-3-hordein	*H. vulgare*	I6TEV2	QQCCQQLANINEQSR	1.24
D-Hordein	*H. vulgare*	Q84LE9	QYEQQTEVPSK	-0.82
B3-hordein	*H. vulgare*	I6TRT5	SQMLQQSSCHVLQQQCCQQLPQIPEQLR	-0.99
B3-hordein	*H. vulgare*	I6TRT5	TLPTMCSVNVPLYR	-0.37
B3-hordein	*H. vulgare*	I6TRT5	VFLQQQCSPVAMSQR	5.01
B-hordein	*H. vulgare*	Q3YAF9	VFLQQQCSPVR	9.78
Avenin	*A. sativa*	P27919	ALPVDVLANAYR	1.13
Avenin	*A. magna*	I4EP67	CPAIHSVVQAIILQK	-0.36
Avenin	*A. sativa*	P27919	ELGGFFGTQQGLIGK	3.23
Avenin	*A. sativa*	P27919	NECCQLLGQMPSECR	10.51
Gliadin-like avenin	*A. sativa*	L0L4J7	QFLVQQCSPVAAVPFLR	0.69
Avenin	*A. magna*	I4EP67	QFLVQQCSPVAEVPFLR	2.00
Avenin	*A. magna*	I4EP67	QQCCQQLAQIPEQVR	10.49
Avenin	*A. magna*	I4EP67	SQILQQSSCQVMK	2.70
Gliadin-like avenin	*A. sativa*	L0L4J7	SQILQQSSCQVMR	2.58

Supplementary Table 10. Gluten peptides detected in powdered drinks (D). The spectral dataset was searched against the Poaceae subset of the Uniprot database. Only peptides with ≥95% confidence in this study abiding by trypsin digestion rules are reported. The mass error (ΔM) is presented in parts per million (ppm).

Protein Names	Species	Accessions	Peptide Sequence	ΔM (ppm)
High molecular weight glutenin subunit 1Dy3	*T. aestivum*	A0A0K0KDM6	ACQQVMDQQLR	1.15
Low molecular weight glutenin subunit	*T. aestivum*	Q75ZV8	AIIYSIVLQEQQQVR	0.34
High molecular weight glutenin subunit 1Dy3	*T. aestivum*	A0A0K0KDM6	AQQPATQLPTVCR	0.75
Alpha-gliadin	*T. aestivum*	A0A1K0JNF2	CQAIHNVAHAIIMHQQQQQQQEQK	-2.18
Avenin-like a4	*T. aestivum*	D2KFH1	CQAVCSVAQVIMR	0.96
Avenin-like a3	*T. aestivum*	P0CZ08	CQAVCSVSQIIMR	1.02
Avenin-3	*T. urartu*	M8ANS4	DALLQQCSPVADMSFLR	2.19
High molecular weight glutenin subunit 1Dy3	*T. aestivum*	A0A0K0KDM6	DISPECHPVVVSPVAGQYEQQIVVPPK	-1.89
High molecular weight glutenin subunit	*T. aestivum*	A0A060MZP1	DVSPECQPVGGGPVAR	0.83
Alpha-gliadin	*T. aestivum*	R9XUM8	DVVLQQHNIAHAR	-0.73
High molecular weight glutenin subunit	*T. aestivum*	A0A060MZP1	EGEASEQLQCER	1.45
High molecular weight glutenin subunit 1Bx protein	*T. dicoccoides*	A0A142ESP3	ELEACQQVVDQQLR	5.13
High molecular weight glutenin subunit 1Dy3	*T. aestivum*	A0A0K0KDM6	GGSFYPGETTPPQQLQQR	5.34
Alpha-gliadin	*T. aestivum*	A0A0E3Z589	GSVQPQQLPQFEEIR	-0.77
LMW-glutenin	*T. aestivum*	B5ANT3	ILPTMCSVNVPLYR	0.88
High molecular weight glutenin subunit 1Bx protein	*T. dicoccoides*	A0A142ESP3	LEGSDALSAR	2.94
High molecular weight glutenin subunit y	*T. aestivum*	Q94IJ6	LPWSTGLQMR	-1.72
Alpha-gliadin	*T. aestivum*	K7X0N8	MDVVLQQHNIVHGR	0.73
Gamma-gliadin	*T. aestivum x Thinopyrum*	B6DQD1	NDCQVMQQQCCQQLAQIPR	2.13
Alpha-gliadin	*T. aestivum*	A5JSA7	NLALQTLPR	0.00
High molecular weight glutenin subunit	*T. aestivum*	A0A060MZP1	QGQQGQQSGQGQPR	-0.49
High molecular weight glutenin subunit y	*T. aestivum*	Q94IJ6	QGQQLEQGQQPGQGQQTR	0.37
LMW-m glutenin subunit 0877L13-M	*T. aestivum*	B2BZD2	QLPQIPEQSR	-0.10
Avenin-like a4	*T. aestivum*	D2KFH1	QQCCQPLAQISEQAR	7.46
High-molecular-weight glutenin subunit	*T. aestivum*	T2HRF3	QQDQQSGQGQQPGQR	0.95

High molecular weight glutenin subunit y	*T. aestivum*	Q94IJ6	QQPGQGQQIGQGQQLGQGR	0.31
High molecular weight glutenin subunit	*T. aestivum*	A0A060MZP1	QQPGQGQQLR	2.14
Avenin-like b2	*T. aestivum*	P0CZ05	QQQPQQQWQGMYQPQQPAQHESIR	-1.40
High molecular weight glutenin subunit y	*T. aestivum*	Q94IJ6	QVVDQQLAGR	-0.88
High molecular weight glutenin subunit	*T. aestivum*	A0A060MZP1	QYEQQVVVPPK	3.16
Gamma-gliadin	*T. aestivum*	B6UKP1	RPLFQLVQGQGIIQPQQPAQLEVIR	-1.28
Low-molecular-weight glutenin subunit	*T. urartu*	A0A089XB95	SQMLEQSICHVMQQQCCQQLR	-7.12
LMW-s KS2	*T. aestivum*	Q68AN2	SQMLQQSSCHMMQQQCCQQLPQIPQ	-6.57
Low molecular weight glutenin subunit	*T. aestivum*	Q75ZV8	SQMLQQSSCHVMQQQCCQQLPQIPQQ	0.78
Low-molecular-weight glutenin subunit	*T. aestivum*	R4JBK0	SQMLWQSSCHVMQQQCCQQLPR	2.24
Low-molecular-weight glutenin subunit	*T. aestivum*	Q1ZZT4	SQMWQQSSCHVMQQQCCQQLPQIPE	1.45
LMW-glutenin	*T. aestivum*	B5ANT3	SQMWQQSSCHVMQQQCCQQLSQIPE	-1.66
LMW-m glutenin subunit 0877L13-M	*T. aestivum*	B2BZD2	SQTLWQSSCHVMQQQCCR	1.26
Alpha-gliadin	*T. aestivum*	K7X0N8	SQVLQQSTYQLLR	-1.41
Avenin-3	*T. urartu*	M8ANS4	SQVVQHSSCLVMWEQCCQQLK	-2.87
High molecular weight glutenin subunit 1Dy protein	*T. aestivum*	V9TRL3	SVAVSQVAR	2.33
Low-molecular-weight glutenin subunit	*T. aestivum*	R4JDK6	TLPTMCNVNVPLYR	0.95
Gliadin/avenin-like seed protein	*T. aestivum*	D2KFH0	TVQSFFEQQLISCR	2.52
LMW-GS	*T. aestivum*	R9XV91	VFLQQQCIPVAMQR	-2.49
Low-molecular-weight glutenin subunit	*T. aestivum*	Q1ZZT4	VFLQQQCSHVAMSQR	-0.40
Low-molecular-weight glutenin subunit Glu-D3	*T. aestivum*	X2JBS3	VFLQQQCSPVAMPQR	2.46
Low molecular weight glutenin subunit	*T. aestivum*	Q75ZV8	VFLQQQCSPVAMPQSLAR	-1.54
Low molecular weight protein	*T. aestivum*	C5IFV2	VFLQQQCSPVVMPQR	0.94
Alpha-gliadin	*T. aestivum*	K7WV42	VPVPQLQPK	3.58
Alpha-gliadin	*T. aestivum*	A0A0E3Z7F3	VPVPQLQPQNPSQQQPQK	4.79
High molecular weight glutenin subunit y	*T. aestivum*	Q94IJ6	VQQPATQLPIMCR	2.67
High molecular weight glutenin subunit	*T. aestivum*	A0A060MZP1	YYPTSPQQPGQEQQPR	-0.77
B3 hordein	*H. vulgare*	I6TRT5	AIVYSIVLR	3.43
D-Hordein	*H. vulgare*	Q84LE9	AQQLAAQLPAMCR	0.84

D-Hordein	*H. vulgare*	Q84LE9	DVSPECRPVALSQVVR	-2.63
Gamma-hordein-3	*H. vulgare*	P80198	EFLLQQCTLDEK	-2.32
D-Hordein	*H. vulgare*	Q84LE9	ELQESSLEACR	-0.46
B hordein	*H. vulgare subsp. vulgare*	C7FB16	ILPFGIDTR	4.03
Gamma 1 hordein	*H. vulgare*	I6TMV6	ILQQSSCR	-2.83
B3 hordein	*H. vulgare*	I6TRT5	IVPLAIDTR	2.76
Gamma 3 hordein	*H. vulgare*	I6TEV2	QQCCQQLANINEQSR	1.24
B-hordein	*H. vulgare subsp. vulgare*	I6R4A7	SQMLQQSSCHVLQQQCCQQLPQIPEQF	-3.50
B hordein	*H. vulgare subsp. vulgare*	Q2XQF1	SQMLQQSSCHVLQQQCCQQLPQIPEQIR	-2.18
B3 hordein	*H. vulgare*	I6TRT5	SQMLQQSSCHVLQQQCCQQLPQIPEQL	0.71
D-Hordein	*H. vulgare*	Q84LE9	QYEQQTEVPSK	-11.70
B3 hordein	*H. vulgare*	I6TRT5	TLPTMCSVNVPLYR	-0.81
B3 hordein	*H. vulgare*	I6TRT5	VFLQQQCSPVAMSQR	-0.96
B3-hordein	*H. chilense*	Q4G3S8	VFLQQQCSPVPMPQR	-1.28
B-hordein	*H. vulgare subsp. vulgare*	I6R4A7	VFLQQQCSPVR	1.70
Gamma 1 hordein	*H. vulgare*	I6TMV6	VMQQQCCLQLAQIPEQYK	-0.97
D-Hordein	*H. vulgare*	Q84LE9	VVDQQLVGQLPWSTGLQMQCCQQLR	-2.86
High-molecular-weight glutenin subunit	*S. cereale x T. aestivu*	S5SCQ8	QYEQQPVVPSK	2.16
High-molecular-weight glutenin subunit	*S. cereale x T. aestivu*	S5SCQ8	LEGSDALSTR	1.75
High-molecular-weight glutenin subunit	*S. cereale x T. aestivu*	S5SCQ8	DVSPGCRPITVSPGTR	-1.29
Avenin	*A. strigosa*	I4EP89	CPAIHSVVQAIILQK	0.36
Avenin	*A. strigosa*	I4EP89	QFLVQQCSPVAEVPFLR	-1.94

Supplementary Table 11. Gluten peptides detected in the savoury spread (SS). The spectral dataset was searched against the Poaceae subset of the Uniprot database. Only peptides with ≥95% confidence in this study abiding by trypsin digestion rules are reported. The mass error (ΔM) is presented in parts per million (ppm).

Protein name	Species	Accession	Peptide sequence	ΔM (ppm)
B-hordein	*H. vulgare*	I6R4A7	VFLQQQCSPVR	-1.36
B-hordein	*H. vulgare*	C7FB16	VFLQQQCSPVAMSQR	-2.41
B3-hordein	*H. vulgare*	I6TRT5	IVPLAIDTR	-3.26
B3-hordein	*H. vulgare*	I6TRT5	SQMLQQSSCHVLQQQCCQQLPQIPEQLR	-0.20
B3-hordein	*H. vulgare*	I6TRT5	TLPTMCSVNVPLYR	2.66
B3-hordein	*H. vulgare*	I6TRT5	VFLQQQCSPVAMSQR	-2.41
D-Hordein	*H. vulgare*	Q84LE9	DVSPECRPVALSQVVR	-2.80
D-Hordein	*H. vulgare*	Q84LE9	ELQESSLEACR	-1.23
D-Hordein	*H. vulgare*	Q84LE9	QYEQQTEVPSK	-0.88
γ-3-hordein	*H. vulgare*	I6TEV2	EFLLQQCTLDEK	-0.47
γ-3-hordein	*H. vulgare*	I6TEV2	QQCCQQLANINEQSR	0.55
Avenin-like a4	*T. aestivum*	D2KFH1	CQAVCSVAQVIMR	-0.58
Avenin-like a4	*T. aestivum*	D2KFH1	QQCCQPLAQISEQAR	2.12

Supplementary Table 12. MRM transitions for the wheat, rye, barley and oat peptide markers assessed in food products.

Cereal	PM #	Type	Peptide Sequence	Uniprot Accession	RT (min)	Q1 m/z (z)	Q3 m/z (fragment, z)
Wheat	WPM1	α/β-gliadin	DVVLQQPNIAHASSK	I0IT53	4.02	536.290 (3+)	924.499 (y9, 1+)
							647.349 (y12, 2+)
							696.883 (y13, 2+)
	WPM2	γ-gliadin	APFASIVADIGGQ	B6UKS0	7.12	623.326 (2+)	686.387 (b7, 1+)
							757.424 (b8, 1+)
							872.451 (b9, 1+)
	WPM3	γ-gliadin	SLVLGTLPTMCNVFVPPECSTTK	B6UKS0	8.01	851.093 (3+)	919.419 (y8, 1+)
							1018.487 (y9, 1+)
							1165.556 (y10, 1+)
	WPM4	γ-gliadin	APFASIVAGIGGQ	P08453	6.90	594.325 (2+)	686.387 (b7, 1+)
							757.424 (b8, 1+)
							814.446 (b9, 1+)
	WPM5	γ-gliadin	SLVLQTLPSMCNVYVPPECSIMR	P08453	7.87	898.777 (3+)	989.454 (y8, 1+)
							1088.523 (y9, 1+)
							1251.586 (y10, 1+)
	WPM6	γ-gliadin	SLVLQTLPTMCNVYVPPECSIIR	R9XV62	8.08	897.461 (3+)	971.498 (y8, 1+)
							1070.566 (y9, 1+)
							1233.63 (y10, 1+)
	WPM7	HMW-GS	DVSPGCRPITVSPGTR	Q45R38	3.72	566.958 (3+)	927.526 (y9, 1+)
							699.37 (y13, 2+)
							742.886 (y14, 2+)
	WPM8	HMW-GS	AQQPATQLPTVCR	P10387	3.98	735.379 (2+)	745.403 (y6, 1+)
							974.509 (y8, 1+)
							1142.599 (y10,1+)
	WPM9	LMW-GS	VFLQQQCSPVAMPQR	P10386	5.11	894.947 (2+)	1045.492 (y9, 1+)
							1173.550 (y10, 1+)
							1301.609 (y11, 1+)
	WPM10	LMW-GS	VFLQQQCIPVAMQR	P10385	5.79	859.446 (2+)	974.491 (y8, 1+)
							1102.550 (y9, 1+)
							1230.608 (y10, 1+)

Cereal		Type	Peptide Sequence	Uniprot Accession	RT (min)	Q1 m/z (z)	Q3 m/z (fragment, z)
Rye	RPM1	75K-γ-secalin	NVLLQQCSPVALVSSLR	E5KZQ2	7.51	942.522 (2+)	1188.641 (y11, 1+)
							1316.699 (y12, 1+)
							1444.758 (y13, 1+)
	RPM2	75K-γ-secalin	SLVLQNLPTMCNVYVPR	E5KZQ2	7.24	1002.524 (2+)	1236.586 (y10, 1+)
							1349.670 (y11, 1+)
							1463.713 (y12, 1+)
	RPM2*	75K-γ-secalin	SLVLQNLPTM*CNVYVPR	E5KZQ2	7.51	1010.521 (2+)	1252.581 (y10, 1+)
							1365.665 (y11, 1+)
							1479.708 (y12, 1+)
	RPM3	75K-γ-secalin	QCSTIQAPFASIVTGIVGH	E5KZQ2	8.33	993.509 (2+)	1197.663 (y12, 1+)
							1268.700 (y13, 1+)
							1396.758 (y14, 1+)
	RPM3*	75K-γ-secalin	*QCSTIQAPFASIVTGIVGH	E5KZQ2	9.13	984.996 (2+)	1197.663 (y12, 1+)
							1268.700 (y13, 1+)
							1396.758 (y14, 1+)
	RPM4	75K-γ-secalin	EGVQILLPQSHK	E5KZQ5	4.84	674.883 (2+)	596.315 (y5, 1+)
							709.399 (y6, 1+)
							822.483 (y7, 1+)
	RPM5	75K-γ-secalin	QHVGQGALAQVQGIIQPQQLSQLEVVR	E5KZQ5	7.17	975.540 (3+)	1296.727 (y11, 1+)
							1424.786 (y12, 1+)
							1537.870 (y13, 1+)
	RPM5*	75K-γ-secalin	*QHVGQGALAQVQGIIQPQQLSQLEVVR	E5KZQ5	7.77	1454.294 (2+)	1296.727 (y11, 1+)
							1537.870 (y13, 1+)
							1707.975 (y15, 1+)
	RPM6	75K-γ-secalin	NVLLQQCSPVALVSSVR	E5KZQ6	6.89	935.514 (2+)	1174.625 (y11, 1+)
							1302.683 (y12, 1+)
							1430.742 (y13, 1+)
	RPM7	75K-γ-secalin	QCSTIQAPFASIETGIVGH	E5KZQ6	6.97	1008.496 (2+)	1227.637 (y12, 1+)
							1298.674 (y13, 1+)
							1426.733 (y14, 1+)
	RPM7*	75K-γ-secalin	*QCSTIQAPFASIETGIVGH	E5KZQ6	7.66	999.983 (2+)	1227.637 (y12, 1+)
							1298.674 (y13, 1+)
							1426.733 (y14, 1+)

Cereal		Type	Peptide Sequence	Uniprot Accession	RT (min)	Q1 m/z (z)	Q3 m/z (fragment, z)
Barley	BPM1	A	MVLQTLPSMCR	M0VEH1	5.50	668.323 (2+)	763.338 (y6, 1+)
							864.386 (y7, 1+)
							992.444 (y8, 1+)
	BPM2	B1	VFLQQQCSPVR	I6SJ22	4.17	681.343 (2+)	746.341 (y6, 1+)
							874.399 (y7, 1+)
							1002.458 (y8, 1+)
	BPM3	B3	VFLQQQCSPVAMSQR	I6SW30	4.81	889.927 (2+)	1035.450 (y9, 1+)
							1163.509 (y10, 1+)
							1291.567 (y11, 1+)
	BPM3*	B3	VFLQQQCSPVAM*SQR	I6SW30	4.19	897.927 (2+)	1051.450 (y9, 1+)
							1179.509 (y10, 1+)
							1307.567 (y11, 1+)
	BPM4	B3	IVPLAIDTR	I6SW30	5.26	499.306 (2+)	575.315 (y5, 1+)
							688.399 (y6, 1+)
							785.452 (y7, 1+)
	BPM5	B3	AIVYSIVLR	I6TRT5	6.13	517.324 (2+)	587.388 (y5, 1+)
							750.451 (y6, 1+)
							849.520 (y7, 1+)
	BPM6	B3	VFLQQQCSPVPMPQR	Q4G3S8	5.40	907.956 (2+)	628.324 (y5, 1+)
							1071.507 (y9, 1+)
							1199.566 (y10, 1+)
	BPM6*	B3	VFLQQQCSPVPM*PQR	Q4G3S8	4.80	915.953 (2+)	644.319 (y5, 1+)
							1087.502 (y9, 1+)
							1215.561 (y10, 1+)
	BPM7	D	DVSPECRPVALSQVVR	I6TRS8	4.86	604.646 (3+)	642.854 (y11, 2+)
							755.901 (y13, 2+)
							799.417 (y14, 2+)
	BPM8	D	QYEQQTEVPSK	I6TRS8	2.32	668.823 (2+)	788.415 (y7, 1+)
							916.474 (y8, 1+)
							1045.517 (y9, 1+)
	BPM8*	D	*QYEQQTEVPSK	I6TRS8	3.17	660.323 (2+)	788.415 (y7, 1+)
							916.474 (y8, 1+)
							1045.517 (y9, 1+)

	BPM9	G3	EFLLQQCTLDEK	I6TEV2	5.40	762.374 (2+)	893.403 (y7, 1+)
							1021.462 (y8, 1+)
							1134.546 (y9, 1+)
	BPM10	G3	QQCCQQLANINEQSR	I6TEV2	3.13	938.902 (2+)	1044.543 (y9, 1+)
							1172.602 (y10, 1+)
							1300.661 (y11, 1+)
	BPM10*	G3	*QQCCQQLANINEQSR	I6TEV2	3.84	930.402 (2+)	1044.543 (y9, 1+)
							1172.602 (y10, 1+)
							1300.661 (y11, 1+)
**Cereal**		**Type**	**Peptide Sequence**	**Uniprot Accession**	**RT (min)**	**Q1 m/z (z)**	**Q3 m/z (fragment, z)**
Oats	OPM1	Avenin	QFLVQQCSPVAEVPFLR	I4EP65	7.73	673.356 (2+)	532.324 (y4, 1+)
							760.435 (y6, 1+)
							831.472 (y7, 1+)
	OPM1*	Avenin	*QFLVQQCSPVAEVPFLR	I4EP65	8.95	667.680 (2+)	532.324 (y4, 1+)
							760.435 (y6, 1+)
							831.472 (y7, 1+)
	OPM2	Avenin	SQILQQSSCQVMK	I4EP65	3.84	768.879 (2+)	1208.576 (y10, 1+)
							839.375 (y7, 1+)
							967.434 (y8, 1+)
	OPM2*	Avenin	SQILQQSSCQVM*K	I4EP65	3.10	776.877 (2+)	1224.571 (y10, 1+)
							855.370 (y7, 1+)
							983.428 (y8, 1+)
	OPM3	Avenin	QQCCQQLAQIPEQVR	I4EP65	4.54	943.454 (2+)	628.341 (y5, 1+)
						629.305 (3+)	741.425 (y6, 1+)
						629.305 (3+)	869.484 (y7, 1+)
	OPM3*	Avenin	*QQCCQQLAQIPEQVR	I4EP65	4.54	934.941 (2+)	628.341 (y5, 1+)
						623.630 (3+)	741.425 (y6, 1+)
						623.630 (3+)	869.484 (y7, 1+)
	OPM4	Avenin	CPAIHSVVQAIILQK	I4EP65	6.34	559.655 (3+)	614.424 (y5, 1+)
							685.461 (y6, 1+)
							813.519 (y7, 1+)
	OPM5	Avenin	QLAQIPEQVR	I4EP88	4.26	591.336 (2+)	628.341 (y5, 1+)
							741.425 (y6, 1+)
							869.484 (y7, 1+)

OPM5*	Avenin	*QLAQIPEQVR	I4EP88	5.53	582.822 (2+)	628.341 (y5, 1+)
						741.425 (y6, 1+)
						869.484 (y7, 1+)
OPM6	Avenin	QFLVQQCSPVAVVPFLR	L0L5I0	8.24	994.543 (3+)	532.324 (y4, 1+)
					663.364 (4+)	532.324 (y4, 1+)
					663.364 (4+)	801.498 (y7, 1+)
OPM6*	Avenin	*QFLVQQCSPVAVVPFLR	L0L5I0	9.52	986.030 (3+)	532.324 (y4, 1+)
					657.689 (4+)	532.324 (y4, 1+)
					657.689 (4+)	801.498 (y7, 1+)
OPM7	Avenin	QFLVQQCSPVAAVPFLR	L0L6J0	7.82	980.527 (3+)	532.324 (y4, 1+)
					654.021 (4+)	532.324 (y4, 1+)
					654.021 (4+)	773.467 (y7, 1+)
OPM7*	Avenin	*QFLVQQCSPVAAVPFLR	L0L6J0	9.10	972.014 (3+)	532.324 (y4, 1+)
					648.345 (4+)	532.324 (y4, 1+)
					648.345 (4+)	773.467 (y7, 1+)
OPM8	Avenin	QAICQVTR	L0L6J0	2.34	488.256 (2+)	375.235 (y3, 1+)
						503.294 (y4, 1+)
						663.324 (y5, 1+)
OPM8*	Avenin	*QAICQVTR	L0L6J0	3.74	479.742 (2+)	375.235 (y3, 1+)
						503.294 (y4, 1+)
						663.324 (y5, 1+)
OPM9	Avenin	QLAQIPEQLR	L0L6J0	4.84	598.343 (2+)	642.357 (y5, 1+)
						755.441 (y6, 1+)
						883.500 (y7, 1+)
OPM9*	Avenin	*QLAQIPEQLR	L0L6J0	6.22	589.830 (2+)	642.357 (y5, 1+)
						755.441 (y6, 1+)
						883.500 (y7, 1+)
OPM10	Avenin	QAICQVAR	Q09114	2.39	473.250 (2+)	345.225 (y3, 1+)
						633.314 (y5, 1+)
						746.376 (y6, 1+)
OPM10*	Avenin	*QAICQVAR	Q09114	3.80	464.737 (2+)	345.225 (y3, 1+)
						633.314 (y5, 1+)
						746.376 (y6, 1+)

RT, retention time (min); Q1, precursor ion *m/z*; z, charge, z; Q3, product ion *m/z*; CE, collision energy (V). *Q refers to pyroglutamination of N-terminal Gln and M* refers to oxidation of Met.
